# Metabolite Analysis and Histology on the Exact Same Tissue: Comprehensive Metabolomic Profiling and Metabolic Classification of Prostate Cancer

**DOI:** 10.1038/srep32272

**Published:** 2016-08-31

**Authors:** Tao Huan, Dean A. Troyer, Liang Li

**Affiliations:** 1 Department of Chemistry, University of Alberta, Edmonton, Alberta, Canada; 2 Departments of Pathology and Molecular Biology and Microbiology, Eastern Virginia Medical School, Virginia, US

## Abstract

We report a method of metabolomic profiling of intact tissue based on molecular preservation by extraction and fixation (mPREF) and high-performance chemical isotope labeling (CIL) liquid chromatography mass spectrometry (LC-MS). mPREF extracts metabolites by aqueous methanol from tissue biopsies without altering tissue architecture and thus conventional histology can be performed on the same tissue. In a proof-of-principle study, we applied dansylation LC-MS to profile the amine/phenol submetabolome of prostate needle biopsies from 25 patient samples derived from 16 subjects. 2900 metabolites were consistently detected in more than 50% of the samples. This unprecedented coverage allowed us to identify significant metabolites for differentiating tumor and normal tissues. The panel of significant metabolites was refined using 36 additional samples from 18 subjects. Receiver Operating Characteristic (ROC) analysis showed area-under-the-curve (AUC) of 0.896 with sensitivity of 84.6% and specificity of 83.3% using 7 metabolites. A blind study of 24 additional validation samples gave a specificity of 90.9% at the same sensitivity of 84.6%. The mPREF extraction can be readily implemented into the existing clinical workflow. Our method of combining mPREF with CIL LC-MS offers a powerful and convenient means of performing histopathology and discovering or detecting metabolite biomarkers in the same tissue biopsy.

For over 100 years, histopathology has guided staging and classification of tumors with microscopic evaluation still remaining the gold standard for diagnosis and risk stratification. To improve diagnostic specificity, analysis of biomarkers from tissue samples can be very useful. One important class of chemical biomarkers is the metabolites. Metabolic alterations have long been associated with cancer, prominently including the Warburg effect, shifting energy production toward aerobic glycolysis and generation of lactic acid^1^. The objective of our research is to develop and apply tissue metabolomics for discovering metabolite biomarkers that can be assayed under actual conditions in the clinical setting.

Biomarker assays typically require extraction and disruption of tissue; however, successful implementation of metabolomics in the clinical setting will be more likely to occur if existing needs for histopathology are accommodated. For example, for prostate cancer, the standard of care is sampling 12 cores of different regions of the prostate using an 18 gauge core needle biopsy. This produces cores ranging from 2–5 mg in weight with a diameter of approximately 0.84 mm and 1.0–1.5 mm in length^2^. These cores are first used for histopathology, and then any remaining tissue in the paraffin blocks can be used for additional biomarker testing. In this setting, the application of metabolomics may be constrained if it demands large amounts of tissue or that separate tissue be reserved for cryopreservation as is the current standard for metabolomics. To this end, we have developed a method for extraction and quantitation of metabolite markers called molecular preservation by extraction and fixation (mPREF).

In mPREF, aqueous methanol is used to extract small molecules from tissue while acting as a fixative for preserving tissue architecture. The histology of tissue processed using mPREF is equivalent to that of formalin fixed tissues and is suitable for immunohistochemistry (IHC)^3^. In fact, alcohol fixed tissues often perform better than formalin fixed tissues for extraction of nucleic acids and for IHC, requiring less vigorous antigen retrieval methods^4^. mPREF also avoids the need for cryopreservation which is widely utilized to prepare tissues for metabolite analysis^5^. Thus, any metabolite biomarkers discovered from aqueous methanol extracts could be readily implemented into the current clinical workflow. Only the addition of an analytical step for quantifying the metabolite biomarker(s) is needed, which can be carried out using liquid chromatography multiple-reaction monitoring mass spectrometry (LC-MRM-MS), a technique routinely used for targeted metabolite quantification^6^.

However, discovery of metabolite biomarkers of diseases from aqueous methanol extracts, such as those from prostate needle biopsies, poses several pre-analytical and analytical challenges. One is related to the small sample amount available for analysis, limiting the detection of less abundant metabolites, although the small diameter of these biopsies allows for consistent and complete extraction of the methanol extractable metabolites^7^. Another challenge is normalizing the amount of different samples with varying sizes and compositions for comparative metabolite quantification^8^. Our goal is to adapt metabolomics to clinical workflows while acknowledging and addressing these limitations. We have developed and applied a high-performance chemical isotope labeling (CIL) LC-MS method for profiling the metabolomes of samples prepared by mPREF. Over 4090 metabolites could be quantified using differential ^13^C-/^12^C-dansyl labeling LC-MS, targeting the amine/phenol submetabolome from prostate tissues. We identified seven metabolites to distinguish normal and tumor samples with high sensitivity and specificity. This proof-of-principle study illustrates that the combination of mPREF and CIL LC-MS can be a powerful tool for discovery of potential metabolite biomarkers of tumors or other diseases using clinical tissue samples that also undergo conventional processing for histology.

## Results

### Clinical Characteristics of Subjects

The study protocol was reviewed and approved by the Institutional Review Board of Eastern Virginia Medical School, Norfolk, Virginia. Clinical characteristics of the subjects used in this study are provided in Table 1. All patients had chosen prostatectomy as primary treatment, and cases for inclusion were selected simply based upon whether adequate tumor and appropriate non-tumor methanol extracts were available upon review of the histologic sections that corresponded to the samples. In this study, normal tissue is defined as non-tumor bearing tissue of equivalent glandular/stromal surface area to the tumor bearing tissue. In selecting controls, no biopsies with chronic inflammation were included. When selecting normal tissues to pair with tumor bearing cores, we avoided cores comprised largely of stroma and judiciously attempted to match the cores for both total surface areas of cores and total surface area occupied by glands in a semiquantitative fashion. Core selection was done by Dr. Troyer, a pathologist.

### CIL LC-MS workflow

**Figure 1:**
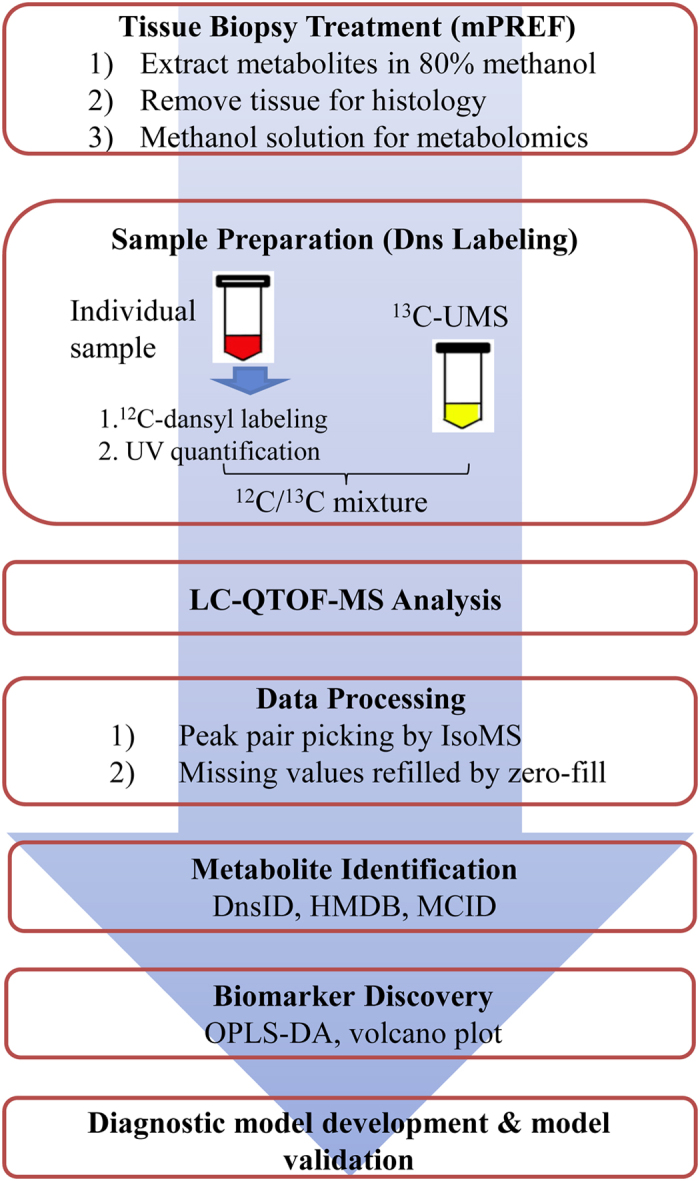
Workflow of high-performance CIL LC-MS for metabolomic profiling and diagnostic model development using mPREF extracts from prostate tumor and normal tissues.

**Figure 2:**
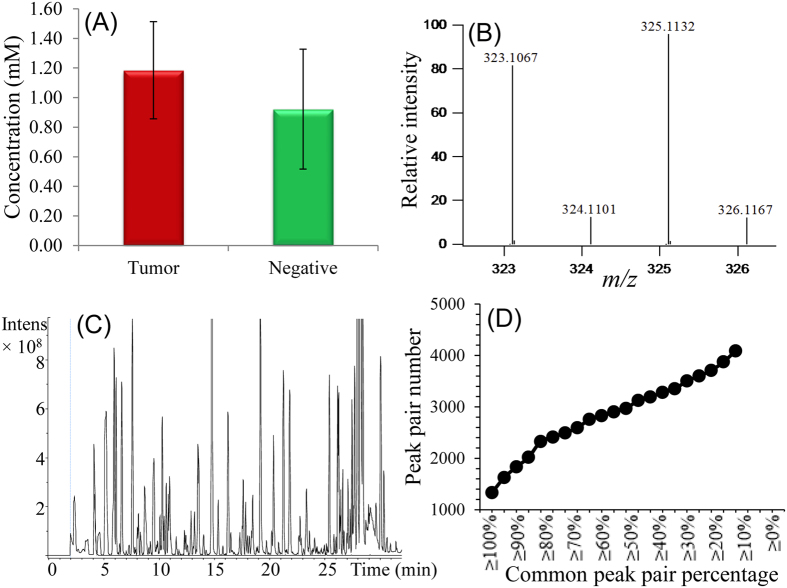
(**A**) Absolute total concentration of labeled metabolites in tumor and normal (negative) mPREF extracted metabolomic samples. (**B**) Example of base-peak ion chromatogram of Dns-labeled sample obtained by LC-MS. (**C**) Example of mass spectral peak pair of a ^13^C-/^12^C-dansyl labeled metabolite (Dns-Alanine). (**D**) Number of peak pairs as a function of common peak pair percentage across all the samples.

Figure 1 shows the overall workflow of combining mPREF and CIL LC-MS for metabolomic profiling of tissue extracts. Each individual sample was ^12^C-dansyl labeled, followed by LC-UV measurement to determine the total concentration of the labeled metabolites. Based on the total concentration information, a proper volume of a labeled sample was taken and mixed with the same molar amount of ^13^C-labeled universal metabolome standard (UMS) generated from a pooled tissue extract (see Methods). The same total molar amount of ^13^C-UMS was used for all the ^12^C-labeled individual samples for metabolome comparison. This way of sample normalization is very important, as it makes it possible to compare the concentration of a given metabolite in different tissue extracts even though the total metabolite concentration of a tumor extract varies significantly from one sample to another. Figure 2A shows the concentration variations of tumor tissues and normal tissues. The relative standard deviation of the total concentration was 27.8% and 44.0% in the tumor and normal tissue samples, respectively. The differences in total labeled metabolite concentration among the individual samples could be as high as 2.6-fold.

To quantify the concentration differences of a metabolite in different samples, we spiked the same ^13^C-UMS to all the ^12^C-labeled individual samples in the discovery and validation sample sets. The individual ^13^C-/^12^C-labeled mixtures were subjected to LC-MS analysis. Figure 2B shows a representative base-peak ion chromatogram. Many chromatographic peaks are detected, indicating the metabolite complexity of tissue extracts. Figure 2C shows an expanded mass spectrum of a ^13^C-/^12^C-labeled metabolite peak pair (Dns-alanine). The peak ratio in the mixture reflects the metabolite’s concentration in the sample referenced to that in the UMS. Since the same UMS was used for all the samples, the ratio values of this metabolite determined from LC-MS analyses of different samples could be used to measure its relative concentration differences among these samples. These ratio values were used for statistical analysis including determining the significant metabolites that differentiate different groups of tissue extracts, while the retention time and m/z values were used for metabolite identification. In this work, dansyl labeling was used to profile the amine/phenol submetabolome^9^, but the same workflow should be applicable to other labeling chemistries targeting different groups of submetabolomes.

### Tissue extract submetabolome

Our initial concern in applying dansylation LC-MS for profiling aqueous extracts was on metabolome detectability, as we expected that only a small amount of metabolites would be extracted through a simple aqueous methanol extraction process from a small needle biopsy. We optimized the workflow (e.g., concentrating the extract) as well as the sample injection amount to maximize the MS detection sensitivity. The latter was done using the ^13^C-/^12^C-UMS mixture with known concentration from the LC-UV measurement. The peak pair numbers detected by LC-MS were plotted against varying sample injection amounts (data not shown). At an injection amount of 5.7 nmol, the peak pair number reached a plateau. Thus, in subsequent experiments, 6 nmol of labeled metabolites from each mixture of ^12^C-sample and ^13^C-UMS was injected into LC-MS for metabolomic profiling.

Using the workflow shown in Fig. 1, a total of 4090 peak pairs or metabolites (not peak features) were detected from the 25 samples in the discovery sample set with an average of 2845 pairs detected per sample. To gauge the detection consistency of our method, Fig. 2D shows a plot of the number of peak pairs detected as a function of the percentage of common pairs detected in all the samples. Among the 4090 peak pairs, 1332 pairs were consistently detected in all the samples and 2900 pairs were consistently detected in 50% of the samples which were retained for statistical analysis. These results illustrate that our method could provide relative quantification information on majority of the metabolites detected across all the samples. We used the 50% inclusion threshold, instead of a higher percentage (e.g., 80%), to avoid the possibility of missing some high-performing metabolites that might have ratio values detected only in 50% to 80% of the samples. As detailed below, several steps were followed in statistical analysis in order to find the potential biomarkers while filtering out possible false findings. These steps include multivariate and binary analysis comparisons for discovering common significant metabolites, manual check of ratio values, box plot analysis of the final biomarkers and receiver operating characteristic (ROC) performance analysis of these biomarkers.

By searching the 4090 peak pairs detected against the Dns-library^10^, 88 metabolites were positively identified based on the mass and retention time matches (see Supplemental Table T1 for the list). Using MyCompoundID MS search^11^, 565 metabolites were putatively identified by accurate mass matches to the HMDB library^12^ (see Supplemental Table T2) and 1196 metabolites matched to the predicted human metabolome library with one reaction^11^ (see Supplemental Table T3). In total, 1761 metabolites were positively or putatively identified, representing about 43% of the 4090 peak pairs detected. This level of detection indicates that the CIL LC-MS method is very sensitive, allowing detecting and quantifying the amine/phenol submetabolome with unprecedented submetabolome coverage.

### Metabolomic comparison of tumor vs. normal tissue extracts

**Figure 3:**
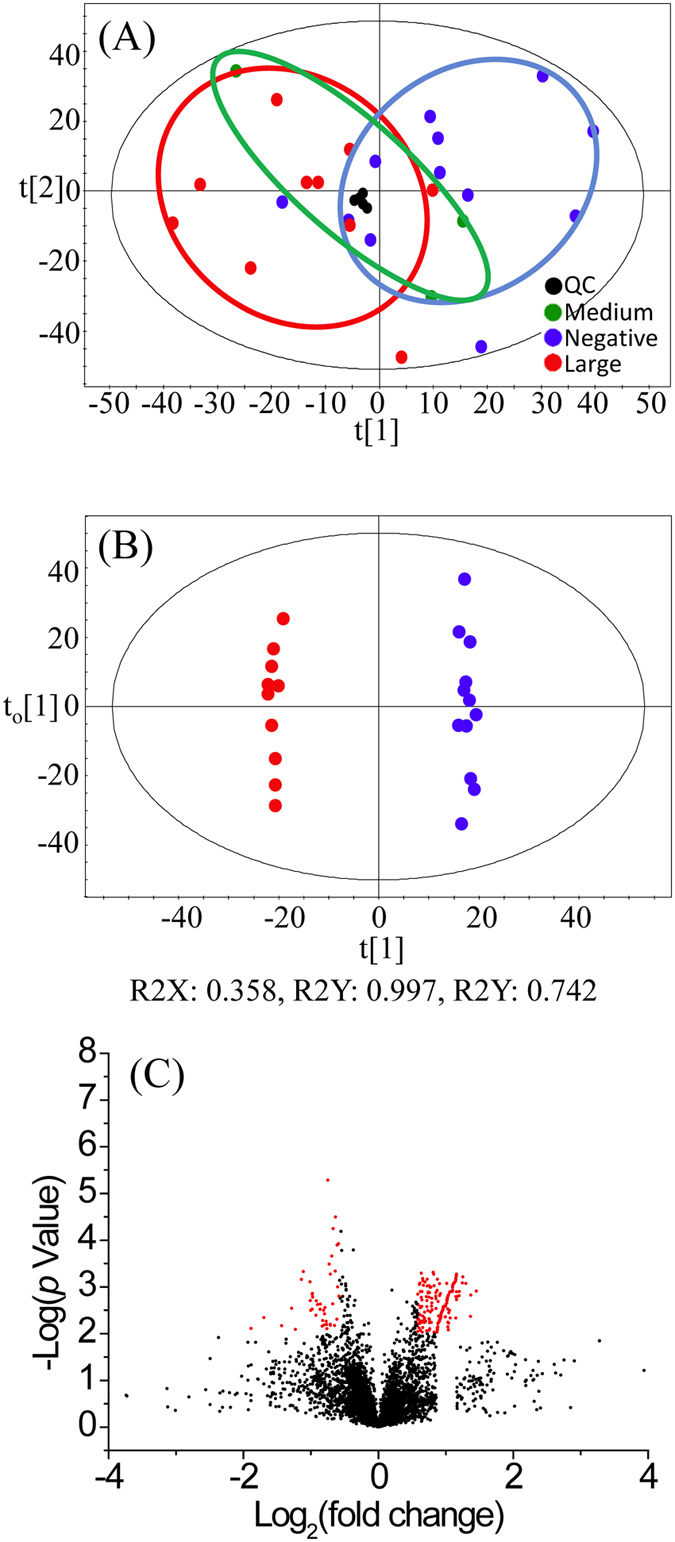
Statistical analysis results of the discovery sample set. (**A**) Score plot of PCA analysis on large tumor, medium tumor, and normal (negative) tissue samples (PC1: 27%, PC2: 13%). (**B**) Score plot of OPLS-DA analysis on large tumor tissues vs. normal tissues (R2X = 0.358, R2Y = 0.997, Q2Y = 0.743. (**C**) Volcano plot of large tumor tissues vs. normal tissues (the red dot represents a metabolite with a fold change ≥1.5, *p*-value ≤ 0.01).

Metabolomic comparison of tumor vs. normal tissue extracts was performed on the discovery sample set consisting of 12 negative controls and 13 tumor tissue extracts (3 medium tumor tissue and 10 large tumor tissue). The main purpose of this comparison was to discover potential biomarkers for tumor tissue classification. Figure 3A shows the PCA score plot of the metabolomic data including the quality control (QC) samples. The QC sample was a mixture of ^12^C-labeled and ^13^C-labeled UMS injected after every 5 individual sample runs. Method blank was prepared using 80% methanol solution incubated in the mPREF container following the same mPREF protocol but without prostate tissue. Only 9 peak pairs were detected after filtering out all the common background peak pairs from reagents and labeling reaction using IsoMS. These 9 peak pairs were excluded in all the sample data. The blank runs contained too many missing values in comparison to QC and sample runs to be included in PCA analysis. As Fig. 3A shows, the QC data are clustered together, indicating good technical reproducibility in LC-MS profiling of all the samples. There is a separation between tumor samples and negative controls. Tissue extracts are collected from patients, which have large biological variation due to genetic and environmental (e.g., life style, diet and medication) factors. Thus the first two principal components in PCA analysis only cover a small amount of variations.

A more distinct separation can be seen using the OPLS-DA model (Fig. 3B). A binary comparison of normal vs. large tumor using OPLS-DA is shown in Fig. 3B, with R^2^Y 0.997 and Q^2^ 0.742. Using a VIP score of larger than 1.5 as a cutoff to select the statistically significant metabolites, 427 metabolites were found to show differences between the normal samples and large tumor samples. The binary comparison was also performed using Volcano plot (Fig. 3C). The significant metabolites with fold change of ≥1.5 or ≤0.67 and *p*-value of ≤0.01 are shown in red dots, while the remaining metabolites are shown in black dots. There were 109 significant metabolites found to be differentially expressed in the normal vs. large tumor samples. The sample size for medium tumor was too small and thus the separation of medium tumors and negative controls was not investigated. Although in the PCA plot the medium tumors appear to be between the normal and large tumor samples which may suggest a progressive change of the metabolome from normal to medium to large tumor, the separation of medium tumors from other samples in an OPLS-DA plot could not be validated, representing an overfitting. Thus direct comparison of medium tumors vs. normal tissues or large tumors would not be meaningful. Future work of increasing the sample number of medium tumors may allow the possibility of direct comparison of this group vs. other groups.

### Diagnostic model for classification of normal and tumor

**Figure 4:**
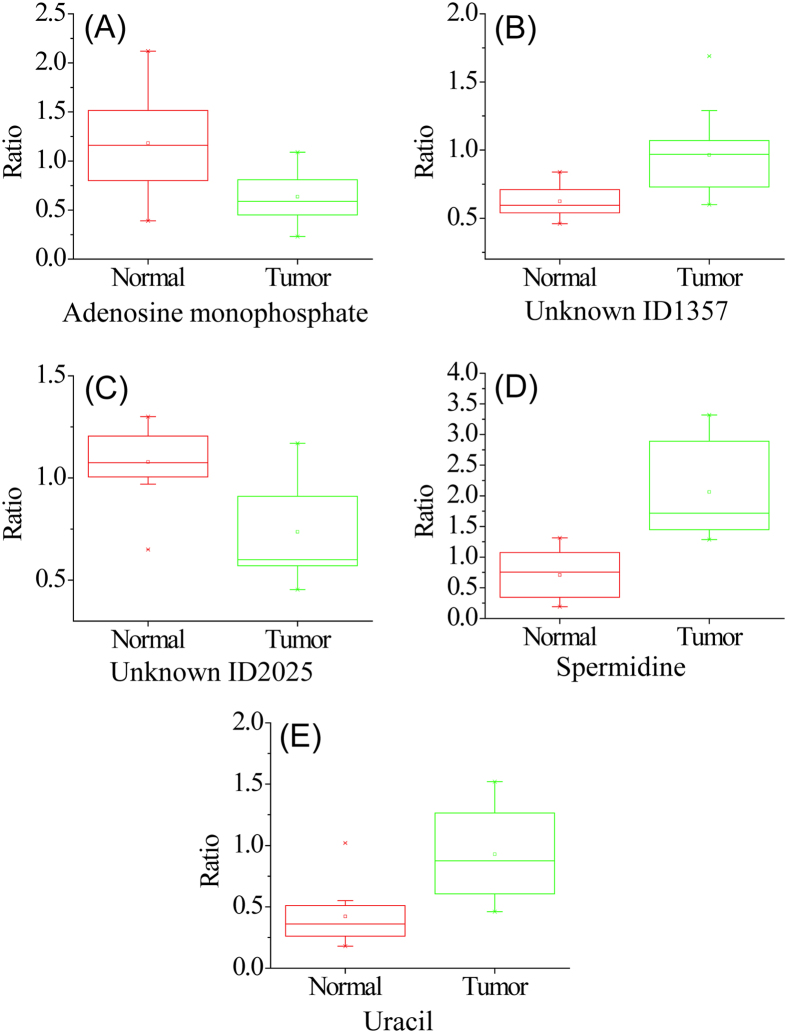
Box plots for the relative metabolic concentrations (normal vs. tumor) of putative biomarkers in the discovery sample set: (**A**) adenosine monophosphate (AMP), (**B**) unknown ID1357, (**C**) unknown ID2025, (**D**) spermidine, and (**E**) uracil.

Combining the two lists of significant metabolites found using OPLS-DA and volcano plots in the discovery sample set (i.e., the training data set), we found 52 common metabolites (Supplemental Table T4) that could be used as potential biomarkers to differentiate the tumor tissues from the normal tissues. Among them, 3 metabolites (adenosine monophosphate, spermidine and uracil) were definitively identified using the dansyl standards library. Two metabolites were putatively matched through matching mass in HMDB to be 4-hydroxyproline and 5-hydroxylysine. However, subsequent analysis of mass, retention time and MS/MS spectrum of the labeled 4-hydroxyproline and 5-hydroxylysine standards did not match with those of the two metabolites. Their structures could not be assigned and thus we designated them as unknown ID1357 and ID2025. All these 5 metabolites show significant concentration change between normal and tumor tissues and good receiver operating characteristic (ROC) performance in the training set. Figure 4 shows the box plot of these 5 metabolites. Finally, a linear vector machine (LVM)-based diagnostic model was built using these 5 potential biomarkers.

**Figure 5:**
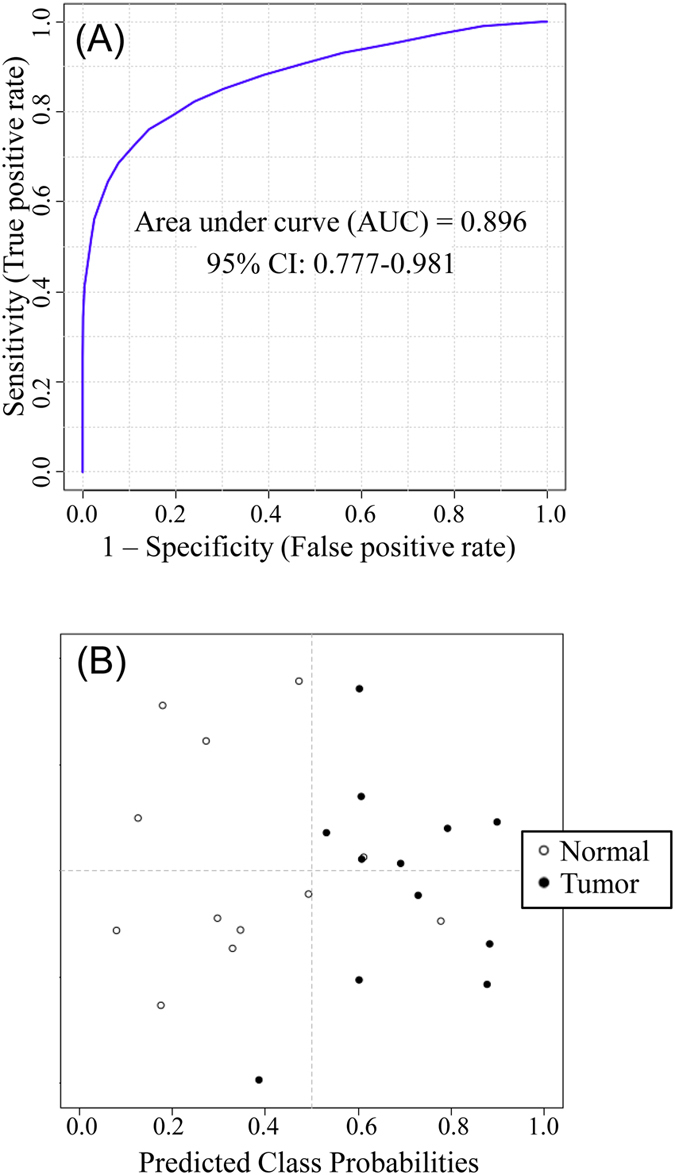
(**A**) Receiver operating characteristic (ROC) analysis with the optimized diagnostic model on the combined training and first validation data sets (a total of 22 tumor and 19 normal tissue samples). (**B**) Prediction of the second validation sample set (blind study) containing 12 tumor and 12 normal tissues (prediction sensitivity: 84.6% and specificity: 90.9%) using the same optimized diagnostic model.

The initial diagnostic model was then trained on a set of validation samples consisting of 19 tumor and 17 normal tissues derived from 18 subjects. The model was further optimized by including additional metabolites from the 52 biomarkers in the training set that were consistently discovered in the validation set. The best diagnostic model could be achieved using a combination of the 5 initially identified metabolites and another two top-ranked metabolites based on AUC values. These metabolites were putatively identified by accurate mass matching to the predicted human metabolome library using MyCompoundID MS Search. Supplemental Table T6 shows the fold change, *p*-value and AUC of these 7 potential biomarkers and Supplemental Table T7 shows the peak ratio values from all the samples. This optimized LVM model generated AUC of 0.896 with sensitivity of 0.846 and specificity of 0.833 in the combined training and validation data sets (Fig. 5A). It should be noted that during our data analysis, we found that some potential biomarkers such as ID129 in Supplemental Table T4 show significant statistical difference in the discovery data set, but not so in the validation data set. In our approach, we selected the potential biomarkers that have consistent diagnostic performance in both training and validation data sets. Thus, the metabolites even with top ranked performance in the training dataset were not included in the model development, if their validation dataset performance was not good.

This diagnostic model was then validated using a second set of validation samples containing 12 tumor and 12 normal tissues derived from 12 subjects. The tissue classification was not disclosed to the analytical lab. Applying the LVM-based diagnostic model, tissue classification was generated and the results were compared to the classification determined by histology. Detailed comparison results are shown in Supplemental Table T5. A prediction sensitivity of 84.6% and specificity of 90.9% were achieved for the second validation set (Fig. 5B). This result demonstrates the potential of the 7 metabolites for differentiating normal and tumor tissues of prostate cancer.

## Discussion

For prostate biopsies, the standard of care is sampling 12 cores of different regions of the prostate using an 18 gauge core needle biopsy. Because pairs of normal and tumor cores were obtained from each prostatectomy in this study, each subject served as their own control. This minimizes biological variability unrelated to cancer. The long term goal of using mPREF in prostate cancer is to identify metabolite biomarkers that are prognostic. The trend in clinical medicine is increasingly toward smaller biopsies to provide diagnosis and guide therapy prior to definitive treatment. Thus, the amount of available tissue often remains small and will likely decrease in the future as robotic and image guided methods of tumor localization and sampling advance, while the menu of translational and individualized diagnostic assays expands. The morphological classification of cancers often fails to fully capture biological variability, and increasingly, molecular analysis is required to identify therapeutic targets or for risk stratification. The competition for tissue between histology and quantitative methods of molecular analysis creates a technological chokepoint for biomarker discovery. The gap is therefore between the need for intact tissue for histology versus disruption and extraction of tissue for quantitative molecular analysis. The mPREF technique addresses this gap.

We note that it had previously been assumed that physical disruption of tissues was necessary to perform large-scale metabolite analysis^13^,^14^, thus making the sample unavailable for histology and consuming scarce tissue. It has been suggested that archived paraffin tissues can be used for detection of metabolites using GC-MS^15^. While metabolites may be detectable in paraffin embedded tissue, the assay of metabolites retrieved from paraffin should be carefully considered. For processing into paraffin, tissues are fixed in formalin (an aqueous solution) for variable times of up to more than 24 hrs, dehydrated in graded alcohols, immersed in xylene, impregnated with paraffin, deparaffinized in xylene and metabolites then extracted. There are many variables in this workflow which may alter the metabolite levels. The histology of tissue processed using the mPREF method is equivalent to that of formalin fixed tissues, and it avoids the need for cryopreservation which is widely utilized to prepare tissues for metabolite analysis. The aqueous alcohol used is available at the bedside, minimizing ischemia time. Technical validation studies comparing disruptive extraction to extraction using mPREF demonstrated complete extraction of extractable metabolites from small tissue biopsies within 2 hrs^7^.

As a proof-of-principle, we profiled the metabolomic changes of tissue extracts prepared by mPREF from prostate biopsy samples of tumors and negative controls. To be relevant for clinical decision making, biomarkers for prostate cancer aggressiveness must be available from biopsy samples which are obtained before definitive treatment, such as prostatectomy. A key feature of the mPREF approach is that it optimizes the use of prostate biopsy samples for discovery and validation of biomarkers. The metabolite data in the literature are based on prostatectomy specimens which incur intraoperative ischemia time and provide results after definitive therapy. The total ischemia time is even greater in robotic prostatectomies versus standard non-robotic surgery^16^. Recently developed commercial prognostic tests such as Oncotype DX® Prostate Cancer Test (GenomicHealth, Redwood Cit, CA), Prolaris® (Myriad Genetics, Salt Lake City, UT), and ProMark™ (Metamark, Cambridge, MA) all utilize paraffin embedded biopsies. By extracting metabolites while leaving DNA, RNA and proteins intact in paraffin embedded biopsies, mPREF fits well into this emerging diagnostic landscape. When implemented in the clinic, mPREF will allow both metabolite analysis and paraffin-based tests to be performed.

To perform metabolomic profiling from extracts prepared by mPREF, we developed and applied a high-performance CIL LC-MS method^9^,^17^,^18^ to overcome the technical issues related to sample normalization, metabolite quantification and metabolite detection. The total amount of metabolites in the extracts of tissues with varying sizes and compositions can vary greatly (Fig. 2A). Thus, sample amount normalization to the same total concentration of metabolites is very important in order to determine the individual metabolite concentration differences among the samples^8^. We used an LC-UV method^19^ to measure the total amount of dansyl labeled metabolites in an extract to normalize individual samples. In this approach, we extracted the metabolites from a tissue and labeled the extract using ^12^C-dansylation, followed by LC-UV quantification of the labeled metabolites with the use of a calibration curve of peak area of eluted metabolites vs. varying known concentrations of a labeled amino acid standard mixture^19^. According to the concentration of a labeled extract, we took a proper volume of an aliquot from each ^12^C-labeled extract so that the same molar amount from all individual labeled samples was taken and mixed it with the same amount of ^13^C-labeled UMS. The mixture was analyzed by LC-MS. In LC-MS, the same amount of the mixture (i.e., 6 nmol in 8 μL which was the optimal amount for detecting the largest number of peak pairs) was injected into LC-MS for analysis to ensure similar MS responses from all the samples for accurate quantification.

For metabolite quantification, in conventional LC-MS methods, achieving technical reproducibility over an extended period (and between laboratories) is challenging due to issues such as ion suppression, instrument performance drift, and aging and contamination of LC columns. We used the UMS chemical isotope labeling approach to provide accurate and precise quantification of different batches of samples^17^. The ^13^C-labeled UMS served as a global standard for relative quantification of ^12^C-labeled individual samples. Technical variations were accounted for by analyzing the ^13^C-/^12^C-labeled mixture and determining the peak intensity ratios of individual metabolite peak pairs. Finally, with rational design of the labeling groups such as dansyl for CIL, LC separation efficiency and MS detection sensitivity can be significantly improved (e.g., dansylation increases the detection sensitivity of metabolites by 10- to 1000-fold)^9^. In our work, we applied dansylation LC-MS to profile the amine/phenol submetabolome of tissue extracts and detected 4090 peak pairs or metabolites. The use of other labeling methods to target other groups of submetabolomes^20^,^21^,^22^,^23^,^24^ should further increase the overall coverage. We note that only a fraction of the tissue extract is needed to perform one labeling LC-MS experiment; one extract should be sufficient to carry out multiple labeling reactions. It is clear that CIL LC-MS analysis of samples prepared by mPREF can perform quantitative tissue metabolomics with high metabolomic coverage.

**Figure 6:**
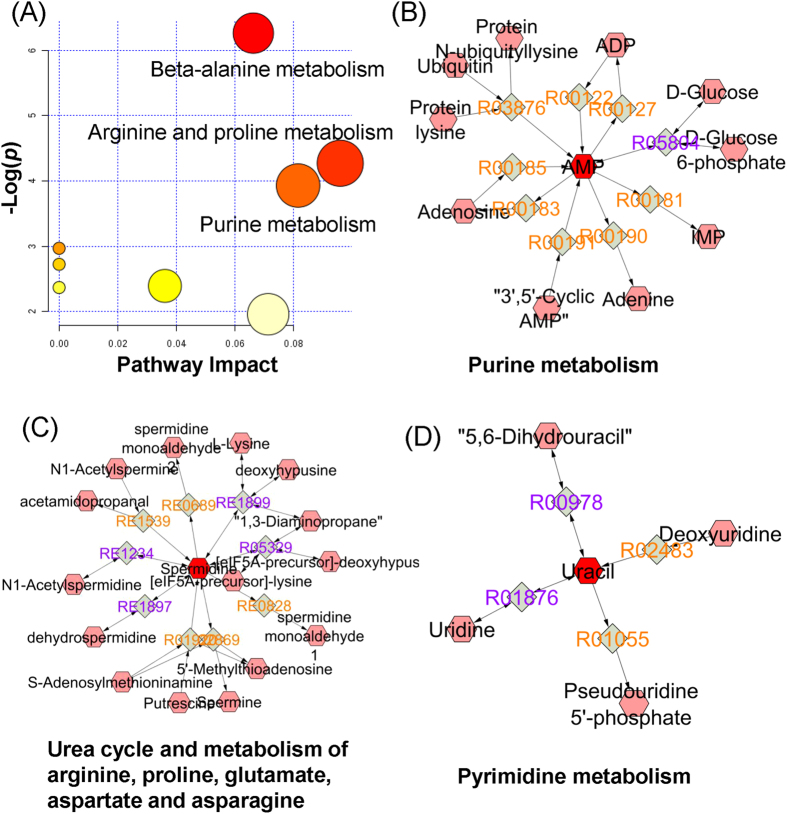
(A) Result of metabolic enrichment analysis. Metabolic activity connections involved from the three identified metabolite biomarkers (hexagon: metabolite, tetragon: metabolic reaction). (**B**) Purine metabolism. (**C**) Urea cycle and metabolism of arginine, proline, glutamate, aspartate and asparagine. (**D**) Pyrimidine metabolism.

Using a total of 85 patient samples, we detected a number of significant metabolites that could be used to separate large tumors and normal controls. Supplemental Figure S1 shows the result of metabolic network constructed with all the identified metabolites as nodes (red dots) and corresponding enzymatic reactions as edges. These metabolites are indicative of particular phenotypes or biological aberrations. Among them, three positively identified significant metabolites, adenosine monophosphate, uracil and spermidine, along with the other five unidentified metabolites, could be used to provide classification of tumors vs. normal with high sensitivity and specificity (Supplemental Table T6). Metabolic pathways enrichment analysis highlights beta-alanine metabolism, arginine and proline metabolism, as well as purine metabolism to be most affected from these potential biomarkers (Fig. 6A). More specifically, metabolic activity connections involved from the three identified biomarkers are shown in Fig. 6B–D, indicating the possible distinguishable metabolic connections in prostate cancer tissue, compared with normal tissue.

Metabolic alterations have long been associated with cancer shifting energy production toward aerobic glycolysis and generation of lactic acid^1^. Prostate cells have a unique metabolic profile and show increased production of polyamines such as spermine and myo-inositol^25^, and secrete very high levels of citrate, resulting partly from the inhibition of Krebs cycle metabolism of citrate. Androgens regulate key enzymes involved in fatty acid and cholesterol synthesis and prostate cancer cells show altered lipid synthesis, including the conversion of citrate to acetyl CoA. Metabolite profiles have been associated with localized and metastatic prostate tissues^26^. Uracil, kynurenine, glycerol-3-phosphate, leucine, and proline were increased in prostate cancer. Recent studies confirm that metabolites can distinguish aggressive versus indolent prostate cancers^27^,^28^,^29^. These studies have been conducted on prostatectomy specimens after clinical decision making is complete. mPREF would permit analysis of metabolites at the time of biopsy, before definitive therapy. Our study is not powered to address aggressive vs indolent prostate cancer. However, in the study of McDunn^27^, uracil, ADP and proline were associated with aggressive cancer, and these or related metabolites were identified in our study. The polyamine spermine was identified in the Giskeodegard study^29^, and our study identified spermidine, a related polyamine.

Adenosine monophosphate (AMP) can be produced from adenosine, adenosine diphosphate (ADP) or adenosine triphosphate (ATP). The reduced level of AMP in tumor tissue may reflect the adenosine, ADP and/or ATP metabolic activity changes. For example, decreased AMP level could be the result of altered activity of AMP-activated protein kinase (AMPK)^30^. AMP can also exist as cyclic Adenosine monophosphate (cAMP) through cAMP phosphodiesterase, a common mechanism for deactivation of cAMP-dependent pathways. Such deactivation, if not performed efficiently, can contribute to the development and/or progression of prostate cancer^31^. In our study, the metabolic concentration of AMP in tumor tissue is half of that in the normal tissue, which may suggest impaired deactivation of cAMP, leading to a higher level of cAMP in the tumor tissue. Note that after the sample was labeled using dansylation, the labeled AMP was found to be very stable.

Uracil is one of the four nucleobases in the nucleic acid of RNA. Extensive incorporation of uracil into human DNA can cause chromosomal breaks increasing the risk of most types of cancers^32^. Such incorporation normally happens if there is a deficiency of any of the micronutrients. It has been reported that intake of Vitamine B6^33^, Vitamine E^34^, and Selenium^35^ intakes are inversely associated with prostate cancer. A significantly higher level of uracil in the tumor tissue found in this work may result in the accumulation of genomic uracil.

Spermidine, one member of the polyamine metabolites, is required for mammalian cell growth and has long been associated with cancer progression^36^. In general, cancer cells produce abundant polyamines that are associated with increased cell proliferation^37^. The up-regulated levels of polyamines are achieved through increased ODC (ornithine decarboxylase) activity^38^ and reduced polyamine efflux^39^. In particular, the over-accumulation of spermidine can further induce the up-regulation of spermidine/spermine N-1-acetyl transferase (SSAT), an enzyme that present significantly higher levels in human prostate cancer tissue samples^40^. It has been recently shown that expression of SSAT in human prostate tissues is related to prostate cancer progression and metastasis^41^.

Sarcosine has previously been reported to be associated with prostate cancer^26^, but subsequent studies showed no association with aggressiveness^42^,^43^,^44^. In our study, sarcosine was detected in high abundance. However, the level differences among the samples were not statistically significant (tumor vs. normal, fold change = 1.2; p-value = 0.6), which is consistent with what was reported in another study of targeted analysis of sarcosine in prostate tissues^44^.

Because of a limited number of samples analyzed, the sensitivity and specificity shown in this work are only preliminary. Future work is needed to validate the prediction capability of these potential biomarkers using larger cohorts, ultimately including samples from multiple centers. Since mPREF is simple to perform, the sample collection protocol should be easily adapted at different sites. CIL LC-MS with UMS is well positioned to analyze different batches of samples collected from different times and sites. We note that the stability of the potential biomarkers described in the current work has not been investigated. In our sample collection, storage and processing workflow, we tried to be as consistent as we could for handling individual samples.

In summary, we have developed a new method of metabolomic profiling and detecting metabolites in methanol/water extracts of tissue samples prepared by mPREF. This method can be readily implemented in current clinical workflows with histology and other parameters assayed on the same tissue. CIL LC-MS can be used to perform quantitative and comprehensive metabolomic profiling of the extracts for discovery of metabolite biomarkers of diseases. Once the biomarkers are validated, we envisage that an LC-MS method based on multiple-reaction-monitoring (MRM) in clinically approved instruments such as triple-quadrupole tandem MS can be developed for quantification of the targeted metabolite(s).

## Materials and Methods

### Tissue sample collection

18 gauge core biopsies were obtained *ex vivo* from prostatectomy specimens and processed using mPREF under IRB approved protocols at Eastern Virginia Medical School (EVMS). Methods were carried out in accordance with guidelines of EVMS including materials transfer agreements provided by the EVMS Office of Technology Transfer and according to protocols approved by the EVMS Institutional Review Board (IRB). Specimens and associated data elements were procured by informed consent following the EVMS IRB protocol “Biospecimen and data banking for the EVMS Biorepository 15-10-FB-0195”. Specimens were utilized according to the EVMS IRB protocol “Molecular Preservation by Extraction (mPREF)13-02-EX-0029-PRIVPRAC”.

18 gauge biopsies were obtained in a fashion intended to mimic the acquisition of biopsies *in vivo*. Each prostate is sampled 12 times in 12 different sites in a systematic fashion to mimic the standard of care in human patients. This produces cores ranging from 2–5 mg in weight with a diameter of approximately 0.84 mm and 1.0–1.5 mm in length. Typically, several of the cores may contain tumor, or as few as one core may contain tumor. In any case, two cores were analyzed from each subject whose prostate was sampled, one a normal/non-tumor core, and one core containing tumor. In this way each subject serves as their own control. Biopsies were immediately placed into 80:20 methanol:water (v/v) and incubated for at least 2 hrs at room temperature. mPREF biopsies were then removed from aqueous methanol and transferred to formalin until processed. The aqueous methanol extract was retained and stored at −80 °C for metabolite analysis, and the tissue was transferred to 10% formalin and processed by standard histopathology methods. Briefly, blocks were sectioned at 4 microns, two sections per level, with three levels placed on each slide and slides stained with hematoxylin and eosin. Tissue extracts were selected for analysis if corresponding histopathology of the biopsies showed tumor occupying 30% or more of the biopsy surface area. Slides with 0–30% were designated “Medium” and those with 30–100% were designated “Large”. Controls without tumor (“Negative”) were selected from cores obtained from the same prostate. A total of 85 tissue extracts were analyzed in this study.

### Dansylation labeling of tissue extracts

360 μL of tissue extract was dried down using SpeedVac and 65 μL (50/50, v/v, H_2_O/acetonitrile (ACN)) was added to re-dissolve the sample. 50 μL of the solution was aliquoted out and mixed with 25 μL H_2_O, 25 μL NaHCO_3_/Na_2_CO_3_ buffer (250 mM, pH = 10.3) and 50 μL ^12^C-dansyl chloride (DnsCl) (18 mg/ml in ACN). The reaction was kept at 40 °C for 45 min. Then 10 μL of NaOH (250 mM) was added into the solution and another 10 min was spent at 40 °C to quench the remaining ^12^C-DnsCl. 50 μL of formic acid solution (425 mM in water) was added to adjust the final pH to ~2.5.

### Preparation of universal metabolome standard (UMS)

A total of 25 biopsy tissue extracts were used as a discovery sample set. 50 μL of re-dissolved extract from each sample was mixed to form a pooled sample which was labeled by ^13^C-dansyl chloride. This ^13^C-labeled pooled sample was stored at −80 °C and used as the universal metabolome standard (^13^C-UMS) for mixing with ^12^C-labeled individual samples for LC-MS analysis.

### LC-UV sample amount normalization

The total amount of the dansyl labeled metabolites in a given sample was quantified using LC-UV, as described previously^19^ (see Supplemental Note N1 for more detail). The total amount was used to normalize the individual samples; an equal amount of ^12^C-labeled individual sample was used and mixed with the same amount of ^13^C-UMS.

### LC-MS analysis

The ^13^C-/^12^C-mixtures were analyzed using a Bruker Maxis Impact QTOF mass spectrometer (Billerica, MA, USA) linked to an Agilent 1100 series binary HPLC system (Palo Alto, CA, USA). The sample injection amount was first optimized using the ^13^C-/^12^C-labeled pooled mixture and then the same optimal amount (6 nmol in 8 μL) was injected for all the mixtures. The detailed instrumental setups can be found in Supplemental Note N1.

### Data processing and metabolite identification

The MS peaks with S/N ≥ 3 in the raw LC-MS data was exported using Bruker Data Analyst software. IsoMS was then used to pick the peak pairs with S/N ≥ 10 from real metabolites and filter out redundant pairs to retain only the protonated molecular ion pair from one metabolite. The ^13^C-/^12^C-peak intensity ratio was calculated for each peak pair, which provides the relative intensity information. The same peak pairs across different samples were aligned together to produce a metabolite-intensity table using IsoMS-align. Missing values in the table were then refilled from the raw LC-MS data using the Zero-fill program^18^. Both training and validation datasets were processed using the same protocol. Definitive metabolite identification was performed by matching retention time (rt) and *m/z* with the dansyl standards library using the DnsID program^10^ with a m/z tolerance of 5 ppm and retention time tolerance of 10 s. Putative metabolite identification was performed by matching accurate mass of a peak pair against the human metabolome libraries (with zero or one reaction) using MyCompoundID (MCID)^11^ with a mass tolerance of 5 ppm.

### Statistical analysis

Prior to statistical analysis, missing values in the metabolite-intensity tables were replaced by peak ratio means to eliminate any potential statistical bias and all the data were preprocessed using auto scaling (i.e., each value subtracts the mean and then divided by the peak ratio standard deviation). Principle component analysis (PCA) and orthogonal partial least squares-discriminant analysis (OPLS-DA) were conducted using SIMCA-P + (Version 12.0) software. Metabolites with VIP score of ≥1.5 in the OPLS-DA analysis were considered as statistically significant. Volcano plot was constructed using Excel and OriginPro 8.5 (OriginLab). Metabolites with fold change of ≥1.5 or ≤0.67 and *p*-value of ≤0.01 were considered statistically important. Metabolites observed as statistically important in both OPLS-DA and volcano plot were further externally validated using two sets of validation samples.

### Diagnostic model development and model validation

To externally validate the metabolite biomarkers, two additional sets of samples as normal/tumor pairs from the same subjects (36 and 24 samples, respectively) were shipped to the University of Alberta for analysis. A prediction metabolite intensity table was extracted from the validation dataset containing all the biomarkers determined in the training dataset. A linear vector machine (LVM)-based diagnostic model was developed using MetaboAnalyst^45^. The LVM model was first constructed using the biomarkers in the training dataset and then applied to the 36-sample set for optimization. The optimized LVM model was further applied on 24-sample set and the predicted results were later compared with the histology derived normal/tumor information to determine the model’s diagnostic power. Notably, the disease and normal classification in the 24-sample set was unspecified and therefore this final analysis can be considered as a blind test.

## Additional Information

**How to cite this article**: Huan, T. *et al*. Metabolite Analysis and Histology on the Exact Same Tissue: Comprehensive Metabolomic Profiling and Metabolic Classification of Prostate Cancer. *Sci. Rep.* **6**, 32272; doi: 10.1038/srep32272 (2016).

## Supplementary Material

Supplementary Information

## Figures and Tables

**Table 1:** Clinical Features of Samples^*^.

	All Subjects	Discovery	Validation Set 1	Validation Set 2
Subjects	46	16	18	12
Age at Diagnosis	59.3 (49.9–70.4)	57.6 (50.4–69.0)	60.7 (49.9–70.4)	60.3 (49.9–70.4)
PSA (ng/mL)	8.39 (0.73–49.4)	7.51 (2.3–18.22)	8.63 (0.73–49.4)	6.70 (3.48–16.7)
BMI (kg/m2)	28.1 (33.5–21.8)	25.9 (19.4–32.1)	29.3 (21.8–39.1)	28.1 (21.8–33.5)
Ethnicity				
Non-Hispanic White	27	8	10	9
African American	18	7	8	3
Hispanic	1	1	0	0
Pathologic Stage at Prostatectomy				
pT2a	2	0	2	0
pT2c	33	12	10	11
pT3a	8	4	3	1
pT3b	3	0	3	0
Gleason Sum Score		6.9	7.0	6.8
3 + 3	7	1	2	4
3 + 4	30	13	12	5
4 + 3	7	2	3	2
4 + 4	1	0	0	1
3 + 5	0	0	0	0
4 + 5	0	0	0	0
5 + 5	0	0	0	0

^*^The table shows age and range of subjects at the time of prostate cancer diagnosis, PSA level and range for the assay closest to the time of prostatectomy, and BMI and range at the time of diagnosis. At prostatectomy, the number of subjects with the pathologic stage and the number of subjects with each Gleason sum score are provided.
