# Supplementary material for: Metabolite Analysis and Histology on the Exact Same Tissue: Comprehensive Metabolomic Profiling and Metabolic Classification of Prostate Cancer
Source: Sci Rep. 2016 Aug 31;6:32272. doi: 10.1038/srep32272 (PMC5006072; doi:10.1038/srep32272)
Supplement: Supplementary Information [file srep32272-s1.pdf]

Supplemental Information

Metabolite Analysis and Histology on the Exact Same Tissue: Comprehensive Metabolomic Profiling and Metabolic Classification of Prostate Cancer

Tao Huan[1], Dean A. Troyer[2*] and Liang Li[1*]

[1]Department of Chemistry, University of Alberta, Edmonton, Alberta, Canada

[2]Departments of Pathology and Molecular Biology and Microbiology, Eastern Virginia Medical School, Virginia, US

Supplemental Note N1. Experimental Section

LC-UV quantification:

Briefly, 2 µL of the labeled solution was injected onto a Waters ACQUITY BEH C18 column (2.1 mm × 5 cm, 1.7 µm particle size, 130 Å pore size). Solvent A was 0.1% (v/v) formic acid in 10% (v/v) acetonitrile, and solvent B was 0.1% (v/v) formic acid in acetonitrile. The gradient started with 0% B for 1 min and was increased to 95% within 0.01 min and hold at 95% B for 1 min. The gradient was restored to 0% B in 0.5 min and hold at this condition for 3.5 min to re-equilibrate the column. The flow rate used was 0.45 mL/min.

LC-MS method:

LC part: Reversed-phase Zorbax Eclipse C18 column (2.1 mm × 100 mm, 1.8 µm particle size, 95 Å pore size) from Agilent was used. Solvent A was 0.1% (v/v) LC-MS grade formic acid in 5% (v/v) grade ACN, and solvent B was 0.1% (v/v) LC-MS grade formic acid in LC-MS grade ACN. The gradient elution profile was as follows: t=0.0 min 20% B, t=3.5 min, 35% B, t=18.0 min, 65%B, t=24 min, 99%B, t=28 min, 99% B. The flow rate was 180 µL/min.

MS part: **QTOF instrument parameters**

1. MS instrument parameter: m/z scan range: 150 to 1000
2. Ion mode: positive ion
3. Source parameters

   End plate offset: 500 V, Capillary: 4500 V, Nebulizer: 1.8 Bar, Dry gas: 8.0 L/min, Dry temperature: 230 ºC.
4. Tune parameters

Funnel 1 RF 250.0 Vpp, Funnel 2 RF: 150.0 Vpp, Hexapole RF: 110.0 Vpp, Quadrupole ion energy: 3.0 eV, Low mass: 100.00, Collision RF 1500.0 Vpp, Transfer time: 80.0 µs. Pre pulse storage 10.0 µs.

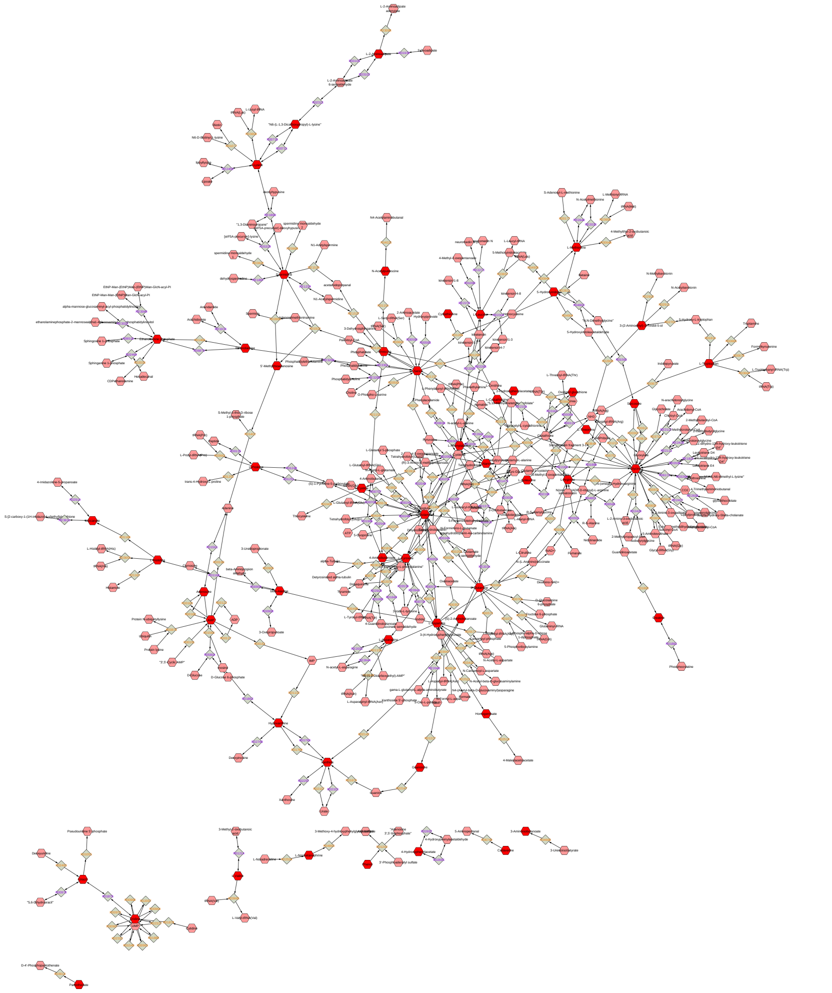

Supplemental Figure S1 (please zoom in to see the detail)

Supplemental Table T1. Metabolites positively identified using dansyl standards library with mass and retention time matches.

#	Input mass	Input rt	Calibrated RT	HMDB No.	Name	Monoisotopic molecular mass	mz_light	Library RT	Mass error (Da)	RT error (min)	HMDB link	KEGG link	Show Detail
1	581.1207	2.05	1.70	HMDB00045	Adenosine monophosphate	347.0631	581.1214	1.75	7.E-04	0.05	Link	Link	Detail
2	517.1496	2.56	2.13	HMDB00133	Guanosine	283.0917	517.1500	2.22	4.E-04	0.09	Link	Link	Detail
3	375.0774	2.58	2.15	HMDB00224	O-Phosphoethanolamine	141.0191	375.0774	2.02	0.E+00	0.13	Link	Link	Detail
4	403.1428	2.59	2.15	HMDB00001	1-Methylhistidine	169.0851	403.1434	2.17	6.E-04	0.02	Link	Link	Detail
	403.1428	2.59	2.15	HMDB00479	3-Methylhistidine	169.0851	403.1434	2.01	6.E-04	0.14	Link	Link	Detail
5	510.1901	2.64	2.19	HMDB00279	Saccharopine	276.1321	510.1905	2.26	4.E-04	0.07	Link	Link	Detail
6	408.1696	2.95	2.45	HMDB00517	L-Arginine	174.1117	408.1700	2.44	4.E-04	0.01	Link	Link	Detail
7	366.1111	3.34	2.88	HMDB00168	L-Asparagine	132.0535	366.1118	3.00	7.E-04	0.12	Link	Link	Detail
8	365.1280	3.38	2.93	HMDB00064	Creatine	131.0695	365.1278	3.02	2.E-04	0.09	Link	Link	Detail
9	380.1279	3.67	3.25	HMDB00641	L-Glutamine	146.0691	380.1275	3.32	4.E-04	0.07	Link	Link	Detail
10	359.1534	3.72	3.31	HMDB01861	3-Methylhistamine	125.0953	359.1536	3.27	2.E-04	0.04	Link	Link	Detail
11	510.1540	3.80	3.40	HMDB11737	Gamma Glutamylglutamic acid	276.0958	510.1541	3.44	1.E-04	0.04	Link	Link	Detail
12	399.1039	4.04	3.66	HMDB02005	Methionine Sulfoxide	165.0460	399.1043	3.72	4.E-04	0.06	Link	Link	Detail
13	501.1545	4.26	3.90	HMDB00050	Adenosine	267.0968	501.1551	3.94	6.E-04	0.04	Link	Link	Detail
14	353.1168	4.45	4.11	HMDB00719	L-Homoserine	119.0582	353.1166	4.05	2.E-04	0.06	Link	Link	Detail
15	399.1037	4.49	4.16	HMDB02005	Methionine Sulfoxide - Isomer	165.0460	399.1043	4.20	6.E-04	0.04	Link	Link	Detail
16	339.1017	4.75	4.44	HMDB00187	L-Serine	105.0426	339.1009	4.40	8.E-04	0.04	Link	Link	Detail
17	381.1115	5.30	5.06	HMDB00148	L-Glutamic Acid	147.0532	381.1115	5.05	0.E+00	0.01	Link	Link	Detail
18	367.0958	5.39	5.16	HMDB00191	L-Aspartic Acid	133.0375	367.0958	5.16	0.E+00	0.00	Link	Link	Detail
19	492.1805	5.75	5.57	HMDB00279	Saccharopine - H2O	276.1321	492.1799	5.65	6.E-04	0.08	Link	Link	Detail
20	353.1171	5.95	5.80	HMDB00167	L-Threonine	119.0582	353.1166	5.79	5.E-04	0.01	Link	Link	Detail
21	395.1267	6.09	5.94	HMDB00510	Aminoadipic acid	161.0688	395.1271	5.97	4.E-04	0.03	Link	Link	Detail
22	295.1114	6.22	6.07	HMDB00149	Ethanolamine	61.0528	295.1111	6.00	3.E-04	0.07	Link	Link	Detail
23	309.0906	6.72	6.57	HMDB00123	Glycine	75.0320	309.0903	6.59	3.E-04	0.02	Link	Link	Detail
24	531.1468	7.20	7.06	HMDB01173	5'-Methylthioadenosine	297.0896	531.1479	6.97	1.E-03	0.09	Link	Link	Detail
25	364.1690	7.24	7.11	HMDB02064	N-Acetylputrescine	130.1106	364.1689	7.25	1.E-04	0.14	Link	Link	Detail
26	406.1423	7.33	7.19	HMDB00721	Glycylproline	172.0848	406.1431	7.17	8.E-04	0.02	Link	Link	Detail
27	323.1065	7.40	7.27	HMDB00056	Beta-Alanine	89.0477	323.1060	7.24	5.E-04	0.03	Link	Link	Detail
28	323.1062	7.72	7.59	HMDB00161	L-Alanine	89.0477	323.1060	7.57	2.E-04	0.02	Link	Link	Detail
29	337.1223	7.94	7.80	HMDB00112	Gamma-Aminobutyric acid	103.0633	337.1216	7.79	7.E-04	0.01	Link	Link	Detail
30	478.1280	8.04	7.90	HMDB00296	Uridine	244.0695	478.1279	7.84	1.E-04	0.06	Link	Link	Detail
31	540.1347	8.28	8.12	HMDB03337	Oxidized glutathione	612.1520	540.1343	8.07	4.E-04	0.05	Link	Link	Detail
32	453.1686	8.48	8.32	HMDB00210	Pantothenic acid	219.1107	453.1690	8.37	4.E-04	0.05	Link	Link	Detail
33	337.1220	8.85	8.67	HMDB03911	3-Aminoisobutanoic acid	103.0633	337.1216	8.67	4.E-04	0.00	Link	Link	Detail
34	370.0970	8.95	8.76	HMDB00157	Hypoxanthine - multi-tags	136.0385	370.0968	8.73	2.E-04	0.03	Link	Link	Detail
35	460.1174	8.97	8.78	HMDB00296	Uridine - H2O	244.0695	460.1173	8.67	1.E-04	0.11	Link	Link	Detail
36	386.0914	9.24	9.04	HMDB00292	Xanthine	152.0334	386.0917	8.95	3.E-04	0.09	Link	Link	Detail
37	408.1585	9.29	9.08	HMDB28854	Glycyl-Valine	174.1004	408.1588	9.19	3.E-04	0.11	Link	Link	Detail
38	337.1226	9.38	9.17	HMDB00452	L-Alpha-aminobutyric acid	103.0633	337.1216	9.13	1.E-03	0.04	Link	Link	Detail
39	363.1012	9.53	9.32	HMDB00148	L-Glutamic Acid - H2O	147.0532	363.1009	9.46	3.E-04	0.14	Link	Link	Detail
40	337.1218	9.58	9.36	HMDB00650	Alpha-aminobutyric acid	103.0633	337.1216	9.23	2.E-04	0.13	Link	Link	Detail
41	323.1059	9.59	9.37	HMDB00271	Sarcosine	89.0477	323.1060	9.34	1.E-04	0.03	Link	Link	Detail
42	363.1013	9.70	9.48	HMDB00148	L-Glutamic Acid - H2O	147.0532	363.1009	9.46	4.E-04	0.02	Link	Link	Detail
43	370.0968	9.85	9.62	HMDB00157	Hypoxanthine - Isomer	136.0385	370.0968	9.65	0.E+00	0.03	Link	Link	Detail
44	370.0976	10.00	9.76	HMDB00157	Hypoxanthine - Isomer	136.0385	370.0968	9.65	8.E-04	0.11	Link	Link	Detail
45	379.2159	10.20	10.91	HMDB01257	Spermidine	145.1579	379.2159	10.8	0.E+00	0.11	Link	Link	Detail
46	514.1656	10.31	10.06	HMDB00706	L-Aspartyl-L-phenylalanine	280.1059	514.1642	10.07	1.E-03	0.01	Link	Link	Detail
47	349.1229	10.47	10.21	HMDB00162	L-Proline	115.0633	349.1216	10.18	1.E-03	0.03	Link	Link	Detail
48	466.2002	10.58	10.32	HMDB29065	Threoninyl-Leucine	232.1423	466.2006	10.18	4.E-04	0.14	Link	Link	Detail
49	351.1382	11.13	10.85	HMDB00883	L-Valine	117.0790	351.1373	10.81	9.E-04	0.04	Link	Link	Detail
50	383.1098	11.21	10.92	HMDB00696	L-Methionine	149.0510	383.1094	10.89	4.E-04	0.03	Link	Link	Detail
51	422.1743	11.48	11.19	HMDB00759	Glycyl-L-Leucine	188.1161	422.1744	11.22	1.E-04	0.03	Link	Link	Detail
52	346.0868	11.70	11.40	HMDB00300	Uracil	112.0273	346.0856	11.34	1.E-03	0.06	Link	Link	Detail
53	436.1900	11.76	11.46	HMDB28691	Alanyl-Leucine	202.1317	436.1901	11.36	1.E-04	0.10	Link	Link	Detail
54	438.1477	11.80	11.50	HMDB00929	L-Tryptophan	204.0899	438.1482	11.44	5.E-04	0.06	Link	Link	Detail
55	470.1737	12.54	12.22	HMDB28694	Alanyl-Phenylalanine	236.1161	470.1744	12.11	7.E-04	0.11	Link	Link	Detail
56	399.1372	13.09	12.77	HMDB00159	L-Phenylalanine	165.0790	399.1373	12.74	1.E-04	0.03	Link	Link	Detail
57	365.1532	13.39	13.05	HMDB00172	L-Isoleucine	131.0946	365.1529	13.06	3.E-04	0.01	Link	Link	Detail
58	365.1529	13.72	13.35	HMDB00687	L-leucine	131.0946	365.1529	13.36	0.E+00	0.01	Link	Link	Detail
59	372.1012	13.95	13.57	HMDB00301	Urocanic acid	138.0429	372.1012	13.52	0.E+00	0.05	Link	Link	Detail
60	364.6245	14.04	13.65	HMDB04987	Alpha-Aspartyl-lysine	261.1325	364.6246	13.61	1.E-04	0.04	Link	Link	Detail
61	345.0922	14.12	13.72	HMDB00099	L-Cystathionine - Isomer	222.0674	345.0920	13.69	2.E-04	0.03	Link	Link	Detail
	345.0922	14.12	13.72	HMDB00455	Allocystathionine - Isomer	222.0674	345.0920	13.61	2.E-04	0.11	Link	Link	Detail
62	364.6239	14.12	13.72	HMDB04987	Alpha-Aspartyl-lysine	261.1325	364.6246	13.61	7.E-04	0.11	Link	Link	Detail
63	315.1088	14.27	13.87	HMDB00450	5-Hydroxylysine	162.1004	315.1085	13.88	3.E-04	0.01	Link	Link	Detail
64	354.0706	14.54	14.11	HMDB00192	L-Cystine	240.0238	354.0702	14.11	4.E-04	0.00	Link	Link	Detail
65	425.1161	15.44	15.00	HMDB00763	5-Hydroxyindoleacetic acid	191.0582	425.1166	15.09	5.E-04	0.09	Link	Link	Detail
66	414.1222	15.76	15.32	HMDB01889	Theophylline	180.0647	414.1230	15.42	8.E-04	0.10	Link	Link	Detail
67	319.1110	16.62	16.17	HMDB03911	3-Aminoisobutanoic acid - H2O	103.0633	319.1110	16.29	0.E+00	0.12	Link	Link	Detail
68	385.1226	16.66	16.21	HMDB01859	Acetaminophen	151.0633	385.1216	16.35	1.E-03	0.14	Link	Link	Detail
69	386.1052	17.02	16.55	HMDB00440	3-Hydroxyphenylacetic acid	152.0473	386.1057	16.72	5.E-04	0.17	Link	Link	Detail
	386.1052	17.02	16.55	HMDB00669	Ortho-Hydroxyphenylacetic acid	152.0473	386.1057	16.42	5.E-04	0.13	Link	Link	Detail
70	386.1049	17.37	16.90	HMDB00020	p-Hydroxyphenylacetic acid	152.0473	386.1057	16.91	8.E-04	0.01	Link	Link	Detail
	386.1049	17.37	16.90	HMDB02390	3-Cresotinic acid	152.0473	386.1057	16.80	8.E-04	0.10	Link	Link	Detail

71	402.1010	17.85	17.38	HMDB00484	Vanillic acid	168.0423	402.1006	17.34	4.E-04	0.04	Link	Link	Detail
72	307.1113	17.94	17.47	HMDB00182	L-Lysine	146.1055	307.1111	17.47	2.E-04	0.00	Link	Link	Detail
73	372.0894	18.11	17.64	HMDB00500	4-Hydroxybenzoic acid	138.0317	372.0900	17.57	6.E-04	0.07	Link	Link	Detail
74	389.1281	18.60	18.13	HMDB00177	L-Histidine	155.0695	389.1278	18.09	3.E-04	0.04	Link	Link	Detail
75	393.1830	19.52	19.06	HMDB00991	2-aminooctanoic acid	159.1259	393.1842	19.20	1.E-03	0.14	Link	Link	Detail
76	395.1048	19.75	19.29	HMDB03320	Indole-3-carboxylic acid	161.0477	395.1060	19.27	1.E-03	0.02	Link	Link	Detail
77	353.1060	20.58	20.13	HMDB29105	Tyrosyl-Glycine	238.0954	353.1060	20.19	0.E+00	0.06	Link	Link	Detail
78	278.1077	21.60	21.15	HMDB01414	1_4-diaminobutane	88.1000	278.1083	21.27	6.E-04	0.12	Link	Link	Detail
79	360.1140	22.31	21.87	HMDB28699	Alanyl-Tyrosine	252.1110	360.1138	21.85	2.E-04	0.02	Link	Link	Detail
80	501.2412	22.50	22.06	HMDB01932	Metoprolol	267.1834	501.2418	22.09	6.E-04	0.03	Link	Link	Detail
81	285.1155	22.77	22.33	HMDB02322	Cadaverine	102.1157	285.1162	22.39	7.E-04	0.06	Link	Link	Detail
82	324.5957	23.10	22.66	HMDB00158	L-Tyrosine	181.0739	324.5953	22.65	4.E-04	0.01	Link	Link	Detail
83	328.1003	23.56	23.12	HMDB00228	Phenol	94.0419	328.1002	23.16	1.E-04	0.04	Link	Link	Detail
84	325.6028	23.80	23.36	HMDB00819	Normetanephrine	183.0895	325.6031	23.41	3.E-04	0.05	Link	Link	Detail
85	342.1156	24.88	24.44	HMDB02048	m-Cresol	108.0575	342.1158	24.44	2.E-04	0.00	Link	Link	Detail
86	322.1052	25.12	24.68	HMDB00259	Serotonin	176.0950	322.1058	24.65	6.E-04	0.03	Link	Link	Detail
87	318.0793	25.39	24.95	HMDB00130	Homogentisic acid	168.0423	318.0794	24.84	1.E-04	0.11	Link	Link	Detail
88	356.1308	25.98	25.54	HMDB29306	4-Ethylphenol	122.0732	356.1315	25.63	7.E-04	0.09	Link	Link	Detail

Supplemental Table T2. Metabolites putatively identified using accurate mass match against the HMDB library.

feature ID	rt (min)	mz_light	mz_heavy	Monoisotopic molecular mass	distance	int_heavy	nCharge	nTag	Matches
18	1.96	449.1137	451.1198	215.0554	2.0061	3.1E+05	1	1	1
26	2.03	722.1662	724.1704	488.1079	2.0042	6.9E+03	1	1	1
28	2.05	581.1207	583.1276	347.0623	2.0070	1.9E+04	1	1	4
37	2.12	403.0623	405.0680	169.0040	2.0056	1.4E+04	1	1	1
44	2.14	375.0775	377.0843	141.0192	2.0067	5.1E+05	1	1	1
46	2.16	389.1273	391.1339	155.0690	2.0066	2.2E+04	1	1	1
69	2.39	541.1417	543.1481	307.0834	2.0064	8.7E+03	1	1	1
78	2.49	359.0735	361.0798	125.0151	2.0063	2.3E+06	1	1	1
84	2.52	366.1002	368.1070	132.0419	2.0068	1.6E+04	1	1	7
88	2.56	517.1496	519.1562	283.0913	2.0066	1.0E+05	1	1	2
94	2.59	403.1428	405.1489	169.0845	2.0061	1.6E+04	1	1	2
98	2.60	414.1209	416.1276	180.0626	2.0067	7.8E+04	1	1	14
103	2.63	501.1533	503.1582	267.0950	2.0049	1.0E+04	1	1	1
104	2.64	510.1901	512.1963	276.1317	2.0062	1.3E+04	1	1	1
119	2.84	456.2213	458.2284	444.3259	2.0072	6.5E+03	2	2	1
123	2.85	509.1700	511.1755	275.1117	2.0055	1.7E+04	1	1	2
128	2.92	414.1213	416.1280	180.0630	2.0067	2.8E+05	1	1	14
129	2.92	424.1174	426.1237	190.0591	2.0064	1.0E+04	1	1	1
133	2.94	375.0771	377.0838	141.0188	2.0067	1.1E+04	1	1	1
135	2.95	408.1696	410.1761	174.1113	2.0065	1.1E+05	1	1	2
151	3.06	424.1167	426.1235	190.0584	2.0068	1.9E+04	1	1	1
156	3.14	518.1337	520.1397	284.0753	2.0061	1.5E+04	1	1	1
157	3.16	496.1383	498.1444	262.0800	2.0061	6.7E+03	1	1	1
161	3.19	366.1007	368.1073	132.0424	2.0066	1.6E+05	1	1	7
163	3.21	502.1391	504.1459	268.0807	2.0068	3.6E+05	1	1	4
164	3.22	422.2100	424.2171	188.1517	2.0071	9.3E+03	1	1	1
165	3.24	345.1383	347.1445	111.0800	2.0062	5.4E+04	1	1	1
171	3.28	417.0777	419.0828	183.0194	2.0050	1.4E+04	1	1	1
173	3.29	581.1214	583.1278	347.0630	2.0064	1.1E+04	1	1	4
176	3.36	515.1720	517.1761	281.1137	2.0041	9.4E+03	1	1	4
177	3.36	468.1429	470.1499	234.0846	2.0070	7.5E+03	1	1	1
182	3.38	365.1280	367.1341	131.0697	2.0061	1.6E+05	1	1	1
187	3.41	375.0767	377.0834	141.0184	2.0067	9.1E+03	1	1	1
192	3.41	424.1533	426.1590	190.0950	2.0057	1.1E+04	1	1	1
207	3.52	359.0731	361.0788	125.0148	2.0057	9.6E+03	1	1	1
237	3.67	380.1279	382.1343	146.0696	2.0065	2.5E+06	1	1	4
245	3.72	359.1534	361.1594	125.0951	2.0059	1.1E+04	1	1	2
246	3.72	436.2006	438.2069	202.1423	2.0063	9.8E+03	1	1	2
255	3.80	510.1540	512.1602	276.0957	2.0061	1.1E+04	1	1	1
264	3.88	438.1326	440.1391	204.0743	2.0064	8.3E+03	1	1	2
266	3.90	518.3183	520.3321	568.5199	2.0139	8.9E+03	2	2	1
267	3.91	409.1532	411.1595	175.0949	2.0064	2.1E+04	1	1	2
282	4.04	399.1039	401.1098	165.0456	2.0060	1.0E+04	1	1	1

304	4.26	501.1545	503.1612	267.0961	2.0067	8.7E+04	1	1	3
327	4.36	366.1007	368.1069	132.0424	2.0062	2.8E+04	1	1	7
328	4.36	414.1209	416.1268	180.0626	2.0058	1.1E+04	1	1	14
337	4.37	501.1547	503.1615	267.0964	2.0067	2.8E+05	1	1	3
350	4.44	380.1269	382.1338	146.0685	2.0069	1.2E+04	1	1	4
353	4.45	353.1168	355.1229	119.0585	2.0061	2.2E+04	1	1	3
365	4.49	399.1037	401.1102	165.0454	2.0065	1.3E+04	1	1	1
366	4.50	451.1636	453.1701	217.1053	2.0065	7.8E+03	1	1	1
404	4.78	380.1274	382.1334	146.0691	2.0060	6.0E+03	1	1	4
410	4.81	381.1113	383.1176	147.0529	2.0063	1.9E+04	1	1	6
415	4.83	478.1268	480.1336	244.0685	2.0068	1.2E+04	1	1	2
459	5.06	523.1852	525.1916	289.1269	2.0064	2.3E+04	1	1	1
494	5.30	381.1115	383.1180	147.0532	2.0065	5.6E+06	1	1	6
503	5.39	367.0958	369.1023	133.0375	2.0065	7.2E+05	1	1	3
505	5.39	414.1212	416.1270	180.0629	2.0058	1.3E+04	1	1	14
637	5.85	734.4366	736.4414	1000.7565	2.0048	4.3E+05	2	2	2
666	5.93	379.1329	381.1382	145.0745	2.0053	1.6E+04	1	1	6
671	5.95	353.1171	355.1235	119.0588	2.0064	5.8E+05	1	1	3
698	6.09	395.1267	397.1332	161.0684	2.0065	7.5E+04	1	1	1
729	6.23	852.4616	854.4656	1236.8065	2.0040	2.8E+04	2	2	2
755	6.31	582.3519	584.3532	696.5871	2.0013	2.8E+04	2	2	1
768	6.33	601.3319	603.3330	367.2736	2.0012	3.2E+04	1	1	1
891	6.48	348.1015	350.1074	114.0431	2.0059	2.4E+04	1	1	2
952	6.53	645.3575	647.3591	411.2992	2.0016	2.9E+04	1	1	1
962	6.54	389.1277	391.1333	155.0694	2.0056	4.7E+04	1	1	1
1076	6.65	505.3014	507.3061	542.4861	2.0047	6.3E+04	2	2	3
1095	6.68	457.0875	459.0938	446.0584	2.0063	1.4E+04	2	2	1
1105	6.72	309.0906	311.0971	75.0323	2.0065	4.8E+06	1	1	1
1114	6.74	704.3956	706.3980	470.3373	2.0023	6.4E+04	1	1	1
1289	6.94	505.3004	507.3048	542.4841	2.0044	8.4E+03	2	2	3
1357	6.98	365.1165	367.1222	131.0582	2.0057	1.3E+04	1	1	8
1366	6.98	308.1425	310.1486	74.0841	2.0062	7.9E+04	1	1	1
1371	6.99	335.1663	337.1723	202.2159	2.0061	1.1E+05	2	2	1
1429	7.02	480.1796	482.1854	246.1212	2.0059	9.1E+03	1	1	2
1448	7.04	669.3243	673.3381	202.2149	4.0138	3.8E+04	1	2	1
1456	7.04	326.1060	328.1122	92.0477	2.0062	8.0E+03	1	1	1
1478	7.07	367.0953	369.1023	133.0370	2.0070	2.1E+04	1	1	3
1519	7.11	616.3649	618.3776	382.3066	2.0127	6.8E+03	1	1	2
1530	7.12	464.1485	466.1540	230.0901	2.0055	9.1E+03	1	1	1
1557	7.16	669.3245	673.3377	202.2151	4.0132	2.7E+04	1	2	1
1561	7.18	637.1257	641.1429	170.0163	4.0172	9.4E+03	1	2	1
1566	7.20	531.1468	533.1537	297.0885	2.0069	2.6E+04	1	1	1
1570	7.22	348.1379	350.1439	114.0796	2.0060	2.0E+04	1	1	1
1575	7.23	478.1110	480.1172	488.1053	2.0062	8.4E+03	2	2	1
1581	7.24	344.1068	346.1131	110.0485	2.0062	1.6E+04	1	1	1
1582	7.24	364.1690	366.1752	130.1107	2.0062	5.6E+04	1	1	1
1588	7.27	335.1663	337.1685	202.2159	2.0023	9.5E+03	2	2	1

1597	7.33	406.1423	408.1496	172.0840	2.0073	1.2E+04	1	1	2
1608	7.42	398.1261	400.1298	164.0678	2.0037	1.5E+04	1	1	6
1620	7.54	366.0639	368.0702	132.0055	2.0063	9.8E+03	1	1	1
1623	7.63	353.1167	355.1229	119.0583	2.0062	6.8E+04	1	1	3
1624	7.65	517.1491	519.1555	283.0908	2.0064	1.9E+04	1	1	2
1625	7.65	366.1001	368.1067	132.0417	2.0067	3.4E+04	1	1	7
1626	7.65	408.1216	410.1284	174.0633	2.0068	2.9E+04	1	1	2
1628	7.72	323.1062	325.1127	89.0478	2.0065	1.1E+07	1	1	4
1630	7.81	592.1236	596.1369	125.0143	4.0132	1.0E+04	1	2	1
1634	7.87	502.1392	504.1457	268.0809	2.0065	6.8E+04	1	1	3
1635	7.87	366.1005	368.1074	132.0422	2.0069	1.2E+05	1	1	7
1636	7.89	460.1168	462.1235	226.0585	2.0066	6.6E+03	1	1	1
1637	7.91	350.1527	352.1595	116.0944	2.0067	7.0E+03	1	1	1
1638	7.93	466.1636	468.1706	232.1053	2.0070	7.7E+03	1	1	2
1642	7.97	385.1074	387.1136	151.0491	2.0062	4.0E+04	1	1	3
1643	7.98	406.1428	408.1489	172.0845	2.0061	8.4E+03	1	1	2
1647	8.04	348.1381	350.1447	114.0798	2.0066	1.1E+05	1	1	1
1648	8.04	366.1005	368.1068	132.0421	2.0064	5.6E+04	1	1	7
1649	8.04	478.1280	480.1345	244.0697	2.0065	3.9E+05	1	1	2
1650	8.09	315.1084	317.1156	162.1002	2.0072	7.3E+03	2	2	1
1653	8.09	396.1354	398.1411	324.1542	2.0057	7.7E+03	2	2	1
1656	8.10	477.1613	479.1664	486.2059	2.0051	1.9E+04	2	2	1
1657	8.10	735.1904	739.2044	268.0810	4.0140	2.3E+04	1	2	3
1659	8.11	599.1517	603.1653	132.0423	4.0136	2.4E+04	1	2	7
1665	8.18	409.1423	411.1482	175.0839	2.0059	2.3E+04	1	1	1
1666	8.19	381.1113	383.1182	147.0530	2.0069	5.9E+04	1	1	6
1673	8.25	396.1106	398.1167	162.0523	2.0061	1.6E+04	1	1	5
1692	8.29	386.0924	388.0983	152.0341	2.0059	1.1E+04	1	1	3
1729	8.33	367.0960	369.1022	133.0377	2.0062	2.0E+04	1	1	3
1732	8.33	322.0741	324.0805	88.0158	2.0064	8.8E+03	1	1	2
1742	8.35	381.1115	383.1179	147.0532	2.0064	3.1E+04	1	1	6
1768	8.41	586.8538	588.8557	705.5910	2.0019	7.4E+03	2	2	1
1769	8.42	422.1740	424.1808	188.1156	2.0068	8.6E+03	1	1	3
1779	8.44	494.1943	496.2004	260.1360	2.0061	9.3E+03	1	1	2
1795	8.45	335.1659	337.1717	202.2151	2.0058	6.2E+03	2	2	1
1802	8.47	396.1107	398.1167	162.0524	2.0060	1.3E+04	1	1	5
1814	8.48	453.1686	455.1752	219.1103	2.0066	3.4E+04	1	1	1
1819	8.50	381.1116	383.1183	147.0533	2.0067	5.2E+04	1	1	6
1849	8.54	669.3254	673.3375	202.2161	4.0121	8.8E+03	1	2	1
1867	8.56	395.1264	397.1333	161.0680	2.0069	2.1E+04	1	1	1
1885	8.59	309.1267	311.1331	75.0684	2.0064	3.2E+04	1	1	2
1903	8.63	366.1003	368.1074	132.0420	2.0071	1.2E+04	1	1	7
1912	8.68	402.0859	404.0926	168.0276	2.0067	1.6E+04	1	1	1
1917	8.74	385.1078	387.1141	151.0495	2.0062	1.7E+05	1	1	3
1922	8.81	395.1273	397.1341	161.0690	2.0068	3.3E+05	1	1	1
1926	8.85	337.1220	339.1286	103.0637	2.0065	3.3E+04	1	1	8
1939	8.94	407.5895	409.5963	347.0624	2.0068	1.6E+04	2	2	4

1941	8.95	370.0970	372.1038	136.0387	2.0067	1.9E+05	1	1	1
1943	8.97	460.1174	462.1240	226.0591	2.0065	5.3E+05	1	1	1
1946	9.02	397.0885	399.0949	163.0302	2.0064	1.0E+04	1	1	1
1950	9.10	309.0907	311.0978	75.0324	2.0071	8.5E+03	1	1	1
1951	9.12	422.1735	424.1816	188.1152	2.0081	8.1E+03	1	1	3
1952	9.19	494.1951	496.2022	260.1368	2.0071	9.8E+03	1	1	2
1954	9.24	398.1265	400.1328	164.0682	2.0064	1.5E+04	1	1	6
1957	9.25	386.0920	388.0983	152.0336	2.0064	9.3E+04	1	1	3
1962	9.29	408.1585	410.1649	174.1002	2.0064	9.1E+03	1	1	1
1967	9.42	307.1113	309.1176	73.0530	2.0063	2.3E+04	1	1	3
1968	9.43	321.0905	323.0972	87.0322	2.0067	2.9E+04	1	1	1
1970	9.45	422.1735	424.1805	188.1152	2.0070	7.3E+03	1	1	3
1972	9.50	425.1198	427.1266	191.0615	2.0068	1.3E+04	1	1	1
1973	9.50	351.1013	353.1072	117.0430	2.0059	1.5E+04	1	1	3
1974	9.53	363.1012	365.1077	129.0429	2.0064	5.5E+04	1	1	5
1977	9.57	376.0961	378.1026	142.0377	2.0065	1.6E+04	1	1	1
1979	9.58	337.1218	339.1283	103.0634	2.0065	9.1E+04	1	1	8
1985	9.59	323.1059	325.1129	89.0476	2.0070	3.5E+04	1	1	4
1986	9.59	321.1268	323.1321	87.0685	2.0053	2.8E+04	1	1	1
1991	9.60	409.1061	411.1132	175.0478	2.0071	4.0E+04	1	1	2
2000	9.70	363.1013	365.1078	129.0430	2.0065	6.4E+06	1	1	5
2002	9.78	341.5866	343.5934	215.0566	2.0068	2.1E+04	2	2	1
2008	9.81	369.0944	371.1001	135.0361	2.0057	2.3E+04	1	1	2
2012	9.85	370.0968	372.1031	136.0385	2.0063	9.2E+04	1	1	1
2015	9.88	321.1269	323.1334	87.0686	2.0064	8.6E+04	1	1	1
2020	9.93	422.1741	424.1801	188.1158	2.0060	2.2E+04	1	1	3
2021	9.94	480.1796	482.1861	246.1213	2.0064	1.1E+04	1	1	2
2025	9.97	315.1086	317.1155	162.1006	2.0069	9.9E+03	2	2	1
2038	10.13	377.1170	379.1233	143.0586	2.0063	2.3E+04	1	1	1
2065	10.35	494.1955	496.2010	260.1372	2.0055	1.1E+04	1	1	2
2068	10.37	660.1463	662.1501	426.0880	2.0038	6.4E+03	1	1	1
2070	10.38	447.1028	449.1094	426.0889	2.0066	8.4E+05	2	2	1
2076	10.39	356.1067	358.1130	122.0484	2.0063	1.0E+04	1	1	1
2095	10.56	323.1063	325.1129	89.0479	2.0066	6.1E+04	1	1	4
2098	10.58	711.3358	715.3492	244.2265	4.0134	1.0E+04	1	2	1
2104	10.62	455.0948	457.1021	221.0365	2.0074	1.7E+04	1	1	1
2124	10.79	348.1378	350.1436	114.0795	2.0057	5.8E+04	1	1	1
2126	10.82	369.1129	371.1194	135.0546	2.0065	9.5E+04	1	1	1
2137	10.91	409.1425	411.1492	175.0842	2.0066	1.7E+04	1	1	1
2139	10.94	349.1219	351.1317	115.0636	2.0098	9.0E+03	1	1	2
2144	11.00	370.0967	372.1030	136.0383	2.0064	4.3E+04	1	1	1
2148	11.02	460.1175	462.1230	226.0592	2.0055	7.6E+03	1	1	1
2174	11.20	379.2159	381.2219	145.1576	2.0060	1.6E+04	1	1	1
2175	11.20	612.2667	616.2804	145.1574	4.0137	3.2E+04	1	2	1
2178	11.21	383.1098	385.1159	149.0515	2.0060	3.9E+05	1	1	1
2185	11.22	306.6375	308.6439	145.1584	2.0064	2.1E+05	2	2	1
2195	11.28	448.1891	450.1956	214.1308	2.0065	1.1E+04	1	1	1

2207	11.31	735.1907	739.2032	268.0814	4.0125	1.7E+04	1	2	3
2234	11.38	612.2668	616.2806	145.1574	4.0139	1.5E+04	1	2	1
2244	11.48	422.1743	424.1803	188.1160	2.0060	2.6E+04	1	1	3
2248	11.50	408.1694	410.1748	174.1111	2.0054	7.3E+03	1	1	2
2249	11.51	321.1142	323.1209	174.1118	2.0067	3.1E+04	2	2	2
2256	11.57	760.3451	762.3469	526.2867	2.0019	1.5E+04	1	1	2
2388	11.75	400.0851	402.0910	166.0267	2.0059	1.5E+04	1	1	3
2390	11.76	612.2673	616.2813	145.1579	4.0140	7.5E+03	1	2	1
2411	11.77	324.0903	326.0968	90.0319	2.0066	1.4E+04	1	1	5
2443	11.80	438.1477	440.1542	204.0894	2.0065	2.2E+05	1	1	1
2465	11.82	337.0856	339.0929	103.0273	2.0073	1.2E+05	1	1	2
2466	11.82	322.1109	324.1169	88.0526	2.0060	1.1E+04	1	1	3
2481	11.83	371.6323	373.6395	275.1481	2.0072	9.4E+03	2	2	2
2562	11.89	521.2875	523.2992	574.4584	2.0116	1.0E+04	2	2	2
2597	11.92	939.5171	941.5196	705.4588	2.0025	2.9E+04	1	1	4
2700	12.21	364.6250	366.6318	261.1334	2.0068	1.3E+04	2	2	3
2706	12.28	337.1219	339.1283	103.0635	2.0065	1.8E+05	1	1	8
2717	12.43	500.2211	502.2245	266.1628	2.0034	1.8E+04	1	1	1
2719	12.50	336.0901	338.0960	102.0318	2.0059	8.5E+03	1	1	6
2723	12.59	578.1894	582.2027	111.0801	4.0132	1.1E+04	1	2	1
2724	12.61	364.0850	366.0902	130.0267	2.0052	9.9E+03	1	1	4
2729	12.72	390.1113	392.1180	156.0530	2.0067	1.3E+04	1	1	3
2733	12.75	592.1245	596.1374	125.0151	4.0129	8.0E+03	1	2	1
2735	12.75	364.6245	366.6311	261.1323	2.0066	1.8E+04	2	2	3
2747	12.88	582.1052	584.1121	348.0469	2.0069	1.2E+04	1	1	3
2750	12.93	396.1103	398.1183	162.0520	2.0080	8.9E+03	1	1	5
2752	12.94	324.0898	326.0967	180.0629	2.0069	2.7E+04	2	2	14
2754	12.95	599.1520	603.1645	132.0427	4.0125	1.3E+04	1	2	7
2755	12.96	735.1910	739.2041	268.0817	4.0130	1.1E+04	1	2	3
2758	12.98	363.1368	365.1433	129.0785	2.0065	2.8E+04	1	1	4
2761	13.00	371.6318	373.6388	275.1471	2.0070	9.1E+03	2	2	2
2762	13.01	355.6010	357.6087	243.0853	2.0077	8.4E+03	2	2	1
2763	13.01	382.5811	384.5876	297.0456	2.0064	2.6E+04	2	2	1
2766	13.05	597.1151	599.1205	363.0568	2.0054	9.9E+03	1	1	2
2769	13.06	368.0985	370.1044	268.0803	2.0059	1.2E+04	2	2	4
2773	13.09	399.1372	401.1437	165.0789	2.0065	1.7E+06	1	1	4
2775	13.11	462.2047	464.2109	228.1464	2.0062	1.3E+04	1	1	2
2778	13.15	296.5655	298.5726	125.0144	2.0071	1.9E+05	2	2	1
2779	13.16	364.6242	366.6312	261.1317	2.0070	7.8E+03	2	2	3
2780	13.27	372.1245	374.1303	276.1323	2.0058	1.6E+04	2	2	1
2797	13.39	365.1532	367.1597	131.0949	2.0065	2.0E+06	1	1	6
2801	13.50	319.1111	321.1176	85.0528	2.0065	2.1E+05	1	1	1
2802	13.50	421.1425	423.1488	187.0841	2.0064	1.3E+04	1	1	1
2803	13.51	462.2046	464.2126	228.1463	2.0080	9.7E+03	1	1	2
2806	13.56	379.1318	381.1381	145.0734	2.0064	2.1E+04	1	1	6
2810	13.58	348.1013	350.1070	114.0429	2.0057	9.8E+03	1	1	2
2826	13.72	365.1529	367.1593	131.0946	2.0064	4.8E+06	1	1	6

2828	13.78	429.1117	431.1178	195.0534	2.0061	2.4E+04	1	1	6
2829	13.79	371.6317	373.6386	275.1468	2.0069	9.1E+03	2	2	2
2834	13.92	367.6061	369.6125	267.0956	2.0063	1.9E+04	2	2	3
2841	13.95	372.1012	374.1068	138.0429	2.0056	8.0E+03	1	1	2
2846	13.96	324.0900	326.0963	180.0634	2.0063	4.0E+04	2	2	14
2862	14.05	300.0796	302.0866	132.0426	2.0070	1.1E+04	2	2	7
2863	14.05	375.6039	377.6106	283.0912	2.0067	1.3E+05	2	2	2
2864	14.05	307.1109	309.1171	73.0526	2.0061	2.2E+04	1	1	3
2867	14.06	423.1215	425.1280	189.0632	2.0065	9.4E+03	1	1	2
2874	14.10	495.1903	497.1965	261.1320	2.0062	6.8E+03	1	1	3
2880	14.10	364.6247	366.6314	261.1327	2.0068	5.7E+04	2	2	3
2882	14.12	345.0922	347.0986	222.0678	2.0064	1.5E+04	2	2	3
2905	14.21	371.6327	373.6391	275.1488	2.0063	1.8E+04	2	2	2
2908	14.24	371.1059	373.1122	137.0476	2.0063	1.3E+04	1	1	5
2909	14.24	416.1154	418.1220	182.0571	2.0066	7.8E+03	1	1	6
2919	14.27	315.1088	317.1158	162.1009	2.0070	7.3E+03	2	2	1
2927	14.32	342.0790	344.0853	108.0207	2.0062	1.1E+04	1	1	2
2930	14.34	333.1270	335.1363	99.0687	2.0093	1.4E+04	1	1	1
2963	14.50	305.0951	307.1018	71.0368	2.0067	9.3E+04	1	1	1
2965	14.51	300.0853	302.0927	132.0540	2.0074	7.5E+03	2	2	4
2968	14.54	354.0706	356.0771	240.0246	2.0065	2.6E+05	2	2	1
2997	14.79	376.0965	378.1030	284.0764	2.0065	9.5E+03	2	2	1
3007	15.02	360.1127	362.1190	126.0544	2.0063	1.1E+04	1	1	2
3013	15.03	366.1006	368.1074	132.0423	2.0069	2.6E+05	1	1	7
3022	15.12	402.0999	404.1058	168.0416	2.0059	1.4E+05	1	1	7
3032	15.29	366.1004	368.1071	132.0421	2.0067	3.0E+04	1	1	7
3037	15.32	776.3307	778.3353	542.2723	2.0046	1.1E+04	1	1	1
3048	15.38	365.1163	367.1230	131.0580	2.0067	1.6E+04	1	1	8
3052	15.44	425.1161	427.1217	191.0578	2.0055	2.0E+04	1	1	1
3069	15.65	368.0987	370.1055	268.0807	2.0068	2.6E+05	2	2	4
3075	15.69	599.1515	603.1653	132.0421	4.0138	2.8E+04	1	2	7
3087	15.76	414.1222	416.1289	180.0639	2.0067	9.3E+03	1	1	17
3102	15.95	389.1274	391.1342	155.0691	2.0067	7.3E+03	1	1	1
3105	15.98	741.3908	743.3936	507.3325	2.0028	7.6E+03	1	1	2
3106	15.98	660.1826	664.1960	193.0732	4.0134	4.1E+04	1	2	3
3110	16.03	342.6297	344.6365	217.1427	2.0069	9.0E+03	2	2	1
3120	16.23	741.3913	743.3961	507.3330	2.0048	8.2E+03	1	1	2
3131	16.30	451.2000	453.2038	217.1417	2.0038	7.2E+03	1	1	1
3132	16.37	397.1243	399.1302	163.0660	2.0059	1.5E+04	1	1	1
3136	16.47	741.3896	743.3916	507.3313	2.0019	9.6E+03	1	1	2
3146	16.62	319.1110	321.1179	85.0527	2.0068	7.8E+04	1	1	1
3147	16.62	368.0990	370.1061	268.0814	2.0071	8.3E+03	2	2	3
3155	16.77	411.1040	413.1104	177.0457	2.0064	8.4E+03	1	1	1
3156	16.78	366.1479	368.1548	132.0895	2.0070	6.2E+03	1	1	2
3158	16.80	300.1035	302.1103	132.0903	2.0068	1.1E+06	2	2	2
3163	16.86	528.1619	532.1754	61.0526	4.0135	7.3E+03	1	2	1
3170	17.02	386.1052	388.1119	152.0469	2.0067	8.2E+03	1	1	9

3171	17.03	425.1157	427.1216	191.0574	2.0060	2.2E+04	1	1	1
3174	17.07	379.1318	381.1386	145.0734	2.0068	1.3E+04	1	1	6
3179	17.13	324.0898	326.0963	180.0629	2.0065	7.8E+03	2	2	14
3184	17.24	741.3895	743.3944	507.3312	2.0049	1.2E+04	1	1	2
3206	17.36	266.0845	268.0911	32.0262	2.0066	1.1E+04	1	1	1
3211	17.45	266.0844	268.0912	32.0261	2.0067	1.4E+04	1	1	1
3212	17.47	423.1000	425.1090	189.0417	2.0091	7.5E+03	1	1	1
3214	17.58	452.1630	454.1687	218.1046	2.0057	1.1E+04	1	1	1
3221	17.66	266.0845	268.0909	32.0262	2.0063	1.6E+04	1	1	1
3227	17.68	308.0948	310.1017	74.0364	2.0069	1.5E+04	1	1	5
3229	17.69	599.1516	603.1650	132.0423	4.0134	3.3E+04	1	2	7
3234	17.71	367.6067	369.6136	267.0968	2.0069	4.3E+05	2	2	2
3244	17.76	356.0931	358.0996	244.0696	2.0065	2.9E+04	2	2	2
3245	17.76	460.1167	462.1230	226.0584	2.0063	1.4E+04	1	1	1
3246	17.76	474.1795	476.1862	240.1212	2.0067	2.5E+04	1	1	3
3249	17.77	354.1198	356.1225	240.1230	2.0027	2.7E+04	2	2	3
3251	17.78	307.1111	309.1179	73.0528	2.0068	1.8E+04	1	1	3
3256	17.81	741.3888	743.3960	507.3305	2.0072	1.4E+04	1	1	2
3260	17.85	711.1777	715.1930	244.0684	4.0152	7.5E+03	1	2	2
3261	17.85	402.1010	404.1066	168.0426	2.0056	1.1E+04	1	1	7
3262	17.85	460.1643	462.1706	226.1060	2.0062	3.8E+04	1	1	1
3264	17.86	347.1115	349.1187	226.1063	2.0072	1.9E+04	2	2	1
3265	17.87	380.1639	382.1704	146.1056	2.0065	1.4E+04	1	1	4
3286	17.94	307.1113	309.1179	146.1060	2.0066	3.0E+06	2	2	4
3290	17.96	407.1629	409.1701	173.1046	2.0072	9.2E+03	1	1	4
3298	18.11	372.0894	374.0959	138.0311	2.0065	6.3E+04	1	1	4
3312	18.34	356.0931	358.0997	244.0696	2.0066	1.2E+04	2	2	2
3320	18.38	427.1313	429.1383	193.0730	2.0070	2.3E+04	1	1	3
3321	18.38	421.2260	423.2319	187.1677	2.0059	9.5E+03	1	1	2
3323	18.39	327.6423	329.6491	187.1680	2.0067	7.0E+04	2	2	2
3326	18.41	308.1424	310.1487	74.0841	2.0063	5.0E+04	1	1	1
3331	18.44	271.1003	273.1061	74.0840	2.0058	1.8E+04	2	2	1
3334	18.44	741.3900	743.3962	507.3317	2.0062	1.6E+04	1	1	2
3337	18.45	436.2731	438.2788	202.2147	2.0057	3.9E+04	1	1	1
3342	18.47	335.1662	337.1730	202.2159	2.0067	3.4E+05	2	2	1
3366	18.58	622.1789	626.1922	155.0696	4.0133	2.7E+05	1	2	1
3369	18.60	389.1281	391.1344	155.0697	2.0063	7.7E+05	1	1	1
3373	18.60	280.1000	282.1065	46.0417	2.0065	1.3E+05	1	1	1
3380	18.67	735.1900	739.2035	268.0807	4.0135	7.0E+03	1	2	4
3381	18.70	462.2048	464.2104	228.1464	2.0056	1.6E+04	1	1	2
3395	18.76	399.1732	401.1794	165.1149	2.0061	5.0E+04	1	1	2
3401	18.81	366.1010	368.1074	132.0427	2.0064	3.2E+04	1	1	7
3402	18.81	750.2008	754.2139	283.0915	4.0131	2.9E+04	1	2	2
3404	18.84	385.1070	387.1138	151.0487	2.0067	3.1E+04	1	1	3
3407	18.84	517.1502	519.1558	283.0919	2.0055	9.3E+03	1	1	2
3414	18.92	750.2011	754.2137	283.0918	4.0126	2.2E+04	1	2	2
3419	18.94	389.1171	391.1256	310.1176	2.0085	9.4E+03	2	2	1

3426	19.02	307.1109	309.1164	146.1052	2.0054	6.8E+03	2	2	4
3434	19.10	393.1472	395.1542	159.0889	2.0070	7.6E+04	1	1	6
3436	19.11	741.3892	743.3950	507.3308	2.0059	2.1E+04	1	1	2
3449	19.17	266.0844	268.0908	32.0261	2.0063	1.0E+04	1	1	1
3457	19.21	379.1684	381.1749	145.1101	2.0064	3.8E+04	1	1	1
3459	19.21	366.1008	368.1074	132.0425	2.0066	7.6E+05	1	1	7
3465	19.23	502.1388	504.1454	268.0804	2.0067	1.3E+04	1	1	4
3468	19.26	357.0900	359.0968	123.0316	2.0068	2.4E+04	1	1	2
3484	19.41	735.1898	739.2035	268.0805	4.0136	4.0E+04	1	2	4
3497	19.54	358.6008	360.6076	249.0850	2.0068	3.2E+04	2	2	2
3505	19.66	336.0902	338.0966	102.0319	2.0063	8.8E+03	1	1	6
3509	19.70	741.3898	743.3940	507.3315	2.0042	1.5E+04	1	1	2
3513	19.74	699.3688	701.3795	465.3104	2.0107	1.3E+04	1	1	2
3523	19.81	379.1683	381.1746	145.1100	2.0063	9.8E+04	1	1	1
3525	19.83	356.0927	358.0996	244.0687	2.0069	1.1E+04	2	2	2
3533	20.03	598.0994	600.1054	364.0411	2.0060	3.0E+04	1	1	1
3545	20.20	711.1779	715.1922	244.0686	4.0143	1.2E+04	1	2	2
3548	20.23	460.1177	462.1226	226.0594	2.0049	9.4E+03	1	1	1
3557	20.27	298.0877	300.0943	128.0587	2.0067	3.7E+05	2	2	1
3558	20.27	356.0931	358.0997	244.0695	2.0066	2.1E+04	2	2	2
3560	20.28	362.1169	364.1227	128.0586	2.0057	8.9E+03	1	1	1
3564	20.32	741.3893	743.3939	507.3310	2.0047	2.3E+04	1	1	2
3568	20.35	346.0746	348.0823	112.0162	2.0077	1.4E+04	1	1	2
3574	20.42	488.1957	490.2019	254.1373	2.0062	8.4E+03	1	1	1
3586	20.54	741.3906	743.3956	507.3323	2.0050	1.3E+04	1	1	2
3588	20.56	294.1161	296.1193	60.0578	2.0032	1.7E+04	1	1	2
3602	20.76	741.3908	743.3947	507.3325	2.0039	1.3E+04	1	1	2
3614	20.87	321.0664	323.0733	174.0162	2.0069	1.9E+04	2	2	3
3621	20.91	300.1033	302.1096	132.0900	2.0062	7.4E+03	2	2	2
3625	20.93	396.0929	398.0989	162.0346	2.0059	8.1E+03	1	1	1
3634	20.98	699.3690	701.3704	465.3106	2.0014	1.3E+04	1	1	2
3642	21.05	423.1404	425.1462	189.0821	2.0058	1.4E+04	1	1	1
3646	21.09	711.1792	715.1927	244.0699	4.0135	8.9E+03	1	2	2
3649	21.12	366.1005	368.1063	132.0422	2.0058	1.5E+04	1	1	7
3651	21.13	478.1267	480.1331	244.0684	2.0064	9.8E+03	1	1	2
3652	21.13	346.0860	348.0925	112.0277	2.0065	8.3E+03	1	1	1
3654	21.17	336.0900	338.0968	102.0317	2.0067	1.1E+04	1	1	6
3662	21.54	321.1082	323.1151	174.0997	2.0069	6.6E+04	2	2	1
3671	22.04	370.0973	372.1031	136.0390	2.0058	1.3E+05	1	1	1
3672	22.05	735.1901	739.2036	268.0808	4.0135	1.4E+05	1	2	4
3675	22.12	297.0855	299.0921	126.0543	2.0066	2.6E+04	2	2	2
3680	22.27	741.3894	743.3952	507.3311	2.0058	1.6E+04	1	1	2
3696	22.37	396.0935	398.0993	162.0351	2.0058	1.3E+04	1	1	1
3702	22.44	382.1587	384.1652	148.1004	2.0064	1.2E+05	1	1	1
3704	22.44	737.3578	739.3658	503.2994	2.0081	6.3E+03	1	1	6
3710	22.48	365.1315	367.1346	131.0731	2.0031	3.9E+04	1	1	1
3712	22.49	715.3755	717.3804	481.3172	2.0049	6.2E+03	1	1	3

3716	22.50	501.2412	503.2476	267.1828	2.0065	2.6E+04	1	1	1
3719	22.51	741.3901	743.3965	507.3318	2.0064	1.2E+04	1	1	2
3723	22.51	314.1193	316.1259	160.1219	2.0066	4.0E+04	2	2	2
3729	22.53	375.0773	377.0829	141.0190	2.0056	9.2E+03	1	1	1
3743	22.65	687.3443	689.3494	453.2860	2.0051	3.4E+05	1	1	2
3744	22.66	715.3770	717.3821	481.3187	2.0051	6.5E+03	1	1	3
3749	22.70	741.3898	743.3969	507.3315	2.0071	1.1E+04	1	1	2
3755	22.72	370.0961	372.1025	136.0378	2.0064	5.5E+04	1	1	2
3762	22.77	352.0980	354.1041	236.0793	2.0062	1.1E+04	2	2	1
3770	22.78	330.0904	332.0946	192.0642	2.0042	7.3E+03	2	2	1
3771	22.79	382.1102	384.1159	148.0519	2.0057	1.0E+04	1	1	2
3785	22.83	598.0997	600.1057	364.0414	2.0059	7.0E+03	1	1	1
3790	22.89	741.3892	743.3956	507.3309	2.0064	1.3E+04	1	1	2
3802	23.03	370.0975	372.1030	136.0392	2.0055	1.4E+04	1	1	1
3807	23.04	310.0747	312.0824	76.0163	2.0077	7.8E+03	1	1	1
3814	23.06	620.1738	624.1877	153.0644	4.0139	9.1E+03	1	2	1
3815	23.06	741.3917	743.3995	507.3334	2.0079	8.8E+03	1	1	2
3820	23.07	735.1909	739.2075	268.0816	4.0165	7.7E+03	1	2	3
3821	23.07	578.1891	582.2022	111.0798	4.0131	1.1E+05	1	2	1
3828	23.10	636.1943	640.2073	169.0850	4.0130	1.7E+04	1	2	2
3832	23.11	403.1433	405.1496	169.0850	2.0063	1.0E+04	1	1	2
3840	23.11	318.5950	320.6019	169.0734	2.0069	7.1E+03	2	2	4
3841	23.11	325.5961	327.6026	183.0756	2.0065	2.0E+05	2	2	1
3843	23.12	559.1567	563.1697	92.0473	4.0130	7.9E+03	1	2	1
3859	23.15	715.3764	717.3801	481.3181	2.0037	7.1E+03	1	1	3
3865	23.17	289.5981	291.6046	111.0795	2.0066	2.6E+05	2	2	1
3899	23.32	741.3901	743.3973	507.3317	2.0073	9.0E+03	1	1	2
3902	23.33	422.1745	424.1806	188.1162	2.0061	6.6E+03	1	1	3
3912	23.41	715.3743	717.3820	481.3160	2.0076	8.2E+03	1	1	3
3919	23.43	699.3694	701.3801	465.3111	2.0107	7.4E+03	1	1	2
3922	23.47	315.0844	317.0913	162.0522	2.0068	1.8E+04	2	2	5
3927	23.47	741.3909	743.3972	507.3326	2.0063	2.4E+04	1	1	2
3942	23.56	328.1003	330.1065	94.0420	2.0063	5.2E+04	1	1	1
3943	23.56	715.3741	717.3820	481.3158	2.0078	7.7E+03	1	1	3
3945	23.57	741.3909	743.3975	507.3326	2.0066	9.8E+03	1	1	2
3966	23.80	325.6028	327.6103	183.0889	2.0075	1.1E+04	2	2	3
3967	23.80	741.3886	743.3972	507.3302	2.0086	8.5E+03	1	1	2
3971	23.85	366.1001	368.1068	132.0418	2.0067	1.1E+04	1	1	7
3977	23.88	360.1123	362.1199	126.0540	2.0077	7.8E+03	1	1	2
3997	24.01	687.3451	689.3504	453.2868	2.0053	1.4E+06	1	1	2
4005	24.05	370.0969	372.1029	136.0386	2.0061	1.9E+04	1	1	1
4025	24.16	741.3915	743.3984	507.3332	2.0069	9.1E+03	1	1	2
4036	24.22	687.3439	689.3496	453.2855	2.0058	7.5E+05	1	1	2
4043	24.26	715.3742	717.3797	481.3159	2.0055	7.1E+03	1	1	3
4053	24.30	619.1561	623.1618	152.0467	4.0057	1.2E+04	1	2	9
4056	24.31	310.0752	312.0815	152.0337	2.0063	3.8E+04	2	2	3
4057	24.32	618.1589	622.1718	151.0495	4.0129	4.6E+04	1	2	3

4058	24.32	385.1072	387.1137	151.0489	2.0065	7.2E+04	1	1	3
4059	24.33	316.0923	318.0998	164.0680	2.0075	1.4E+04	2	2	6
4070	24.46	741.3906	743.3948	507.3323	2.0042	1.3E+04	1	1	2
4072	24.47	370.0967	372.1017	136.0383	2.0051	6.6E+03	1	1	1
4078	24.51	384.1265	386.1332	150.0682	2.0068	9.5E+03	1	1	6
4086	24.56	741.3909	743.3948	507.3326	2.0039	1.4E+04	1	1	2
4090	24.61	292.1055	294.1119	116.0945	2.0064	7.9E+03	2	2	1
4095	24.67	680.2517	682.2586	446.1934	2.0070	7.6E+03	1	1	1
4120	24.76	985.3646	989.3855	518.2553	4.0209	1.2E+04	1	2	1
4125	24.78	299.0715	301.0780	130.0264	2.0065	1.1E+04	2	2	4
4126	24.79	741.3916	743.3977	507.3333	2.0061	1.3E+04	1	1	2
4145	24.83	309.5830	311.5896	151.0495	2.0066	3.1E+05	2	2	3
4154	24.88	342.1156	344.1223	108.0572	2.0068	8.1E+03	1	1	4
4156	24.89	370.0963	372.1027	136.0380	2.0064	7.8E+04	1	1	1
4158	24.89	302.0778	304.0844	136.0389	2.0066	6.1E+05	2	2	1
4163	24.94	564.3343	566.3400	330.2759	2.0057	9.9E+03	1	1	2
4165	24.96	741.3905	743.3964	507.3322	2.0059	9.3E+03	1	1	2
4166	24.96	564.3356	566.3429	330.2773	2.0073	6.9E+03	1	1	2
4167	24.97	354.1157	356.1225	120.0574	2.0068	9.8E+04	1	1	2
4173	25.01	324.5956	326.6020	181.0745	2.0064	8.4E+03	2	2	5
4174	25.01	338.5925	340.6000	209.0684	2.0075	5.7E+04	2	2	1
4178	25.05	741.3905	743.3970	507.3322	2.0065	9.9E+03	1	1	2
4179	25.05	297.0855	299.0922	126.0544	2.0067	9.5E+03	2	2	2
4183	25.07	687.3434	689.3495	453.2851	2.0061	6.1E+05	1	1	2
4184	25.08	345.6005	347.6066	223.0844	2.0060	7.1E+03	2	2	1
4199	25.13	278.1025	280.1121	88.0884	2.0096	8.2E+03	2	2	1
4205	25.15	354.1159	356.1224	120.0576	2.0065	3.8E+05	1	1	2
4216	25.21	687.3440	689.3502	453.2857	2.0062	5.4E+05	1	1	2
4219	25.25	741.3898	743.3945	507.3315	2.0047	7.2E+03	1	1	2
4226	25.30	564.3340	566.3405	330.2757	2.0065	1.5E+04	1	1	2
4236	25.37	356.1710	358.1780	244.2254	2.0069	1.1E+04	2	2	1
4248	25.39	262.0533	264.0598	27.9950	2.0065	1.2E+04	1	1	1
4253	25.39	358.6017	360.6082	249.0868	2.0065	4.9E+04	2	2	2
4278	25.41	564.3356	566.3398	330.2773	2.0042	1.5E+04	1	1	2
4287	25.42	687.3438	689.3505	453.2855	2.0066	2.5E+05	1	1	2
4318	25.55	564.3340	566.3392	330.2757	2.0052	1.8E+04	1	1	2
4323	25.58	375.0773	377.0837	141.0190	2.0064	1.2E+04	1	1	1
4325	25.58	687.3442	689.3508	453.2859	2.0066	2.7E+05	1	1	2
4329	25.61	373.1230	375.1277	278.1294	2.0047	8.8E+03	2	2	1
4345	25.77	687.3430	689.3494	453.2847	2.0064	3.5E+05	1	1	2
4353	25.82	735.3427	737.3477	501.2844	2.0050	1.7E+04	1	1	4
4371	25.96	287.0792	289.0854	106.0417	2.0063	4.5E+04	2	2	1
4379	25.99	713.3601	715.3649	479.3018	2.0047	4.8E+05	1	1	4
4381	26.00	564.3356	566.3412	330.2773	2.0056	2.3E+04	1	1	2
4382	26.01	375.0771	377.0829	141.0188	2.0058	1.4E+04	1	1	1
4384	26.02	370.0961	372.1027	136.0378	2.0065	3.9E+04	1	1	2
4407	26.10	603.1482	607.1612	136.0389	4.0130	1.4E+04	1	2	1

4416	26.12	323.1137	325.1204	178.1108	2.0067	8.8E+03	2	2	3
4419	26.14	805.3386	807.3426	571.2803	2.0040	1.2E+04	1	1	1
4420	26.14	412.1578	414.1645	178.0995	2.0067	7.3E+03	1	1	1
4427	26.15	687.3439	689.3505	453.2856	2.0066	2.1E+05	1	1	2
4429	26.19	564.3345	566.3390	330.2762	2.0045	3.4E+04	1	1	2
4436	26.26	735.3420	737.3468	501.2837	2.0048	2.7E+04	1	1	4
4440	26.27	344.1065	346.1129	110.0482	2.0064	1.1E+05	1	1	1
4442	26.28	564.3340	566.3397	330.2756	2.0058	2.0E+04	1	1	2
4456	26.41	687.3431	689.3497	453.2848	2.0066	1.0E+05	1	1	2
4457	26.41	713.3581	715.3644	479.2998	2.0062	5.9E+04	1	1	4
4460	26.42	564.3350	566.3405	330.2766	2.0056	2.1E+04	1	1	2
4471	26.52	347.0878	349.0948	226.0590	2.0070	1.0E+04	2	2	1
4475	26.55	370.0963	372.1033	136.0379	2.0070	1.2E+04	1	1	1
4481	26.56	302.0778	304.0844	136.0389	2.0067	1.9E+06	2	2	1
4484	26.57	564.3357	566.3408	330.2774	2.0051	2.9E+04	1	1	2
4488	26.58	713.3598	715.3654	479.3015	2.0057	3.8E+05	1	1	4
4491	26.63	344.1064	346.1132	110.0480	2.0068	2.8E+04	1	1	1
4510	26.69	564.3359	566.3407	330.2776	2.0048	2.6E+04	1	1	2
4515	26.72	687.3437	689.3507	453.2854	2.0070	9.8E+04	1	1	2
4538	26.84	713.3604	715.3654	479.3021	2.0050	1.1E+06	1	1	4
4549	26.88	753.3891	755.3971	519.3308	2.0080	8.7E+03	1	1	1
4564	26.91	385.1071	387.1139	151.0488	2.0068	1.7E+04	1	1	3
4565	26.91	750.2004	754.2138	283.0911	4.0134	1.2E+04	1	2	2
4567	26.91	300.0792	302.0843	132.0418	2.0051	2.0E+04	2	2	7
4568	26.91	564.3350	566.3409	330.2767	2.0059	1.0E+04	1	1	2
4583	26.99	599.1520	603.1638	132.0426	4.0118	1.1E+04	1	2	7
4588	26.99	711.1787	715.1922	244.0693	4.0135	1.8E+04	1	2	2
4589	26.99	460.1166	462.1228	226.0583	2.0062	2.3E+04	1	1	1
4600	27.04	370.0972	372.1027	136.0388	2.0055	2.3E+04	1	1	1
4601	27.05	300.0794	302.0854	132.0422	2.0060	3.9E+04	2	2	7
4604	27.06	735.1907	739.2026	268.0813	4.0120	1.0E+04	1	2	3
4626	27.12	687.3435	689.3501	453.2852	2.0066	1.1E+05	1	1	2
4659	27.26	384.1622	386.1683	150.1039	2.0061	1.2E+04	1	1	3
4661	27.27	323.6055	325.6122	179.0943	2.0068	1.8E+04	2	2	2
4663	27.28	687.3437	689.3491	453.2854	2.0054	2.5E+04	1	1	2
4689	27.40	384.1623	386.1688	150.1039	2.0065	7.5E+04	1	1	3
4695	27.41	300.0797	302.0843	132.0428	2.0046	7.5E+03	2	2	7
4702	27.44	687.3431	689.3496	453.2848	2.0065	3.0E+04	1	1	2
4720	27.52	735.1904	739.2050	268.0811	4.0146	7.1E+03	1	2	3
4736	27.57	687.3447	689.3526	453.2864	2.0079	1.6E+04	1	1	2
4742	27.59	709.3263	711.3294	475.2680	2.0031	2.2E+04	1	1	4
4758	27.61	370.0968	372.1033	136.0385	2.0065	3.3E+05	1	1	1
4775	27.67	735.1908	739.2037	268.0814	4.0129	2.8E+05	1	2	3
4785	27.71	687.3450	689.3506	453.2867	2.0056	2.9E+04	1	1	2
4822	27.79	713.3602	715.3655	479.3019	2.0053	1.3E+06	1	1	4
4858	27.91	729.3894	731.3960	495.3311	2.0066	3.2E+04	1	1	1
4860	27.95	687.3452	689.3510	453.2869	2.0058	1.2E+04	1	1	2

4871	28.08	729.3892	731.3955	495.3308	2.0064	6.4E+04	1	1	1
4873	28.11	687.3443	689.3507	453.2860	2.0063	1.8E+04	1	1	2
4878	28.14	603.1621	607.1741	136.0528	4.0120	1.2E+05	1	2	1
4881	28.16	370.0975	372.1029	136.0391	2.0054	7.9E+04	1	1	1
4886	28.17	735.1897	739.2027	268.0804	4.0130	6.3E+04	1	2	4
4896	28.25	599.1512	603.1624	132.0418	4.0112	2.3E+04	1	2	7
4900	28.26	687.3439	689.3498	453.2856	2.0059	2.8E+04	1	1	2
4912	28.29	735.1876	739.2009	268.0782	4.0134	5.6E+04	1	2	1
4919	28.48	652.3076	654.3143	418.2493	2.0067	2.5E+04	1	1	2
4930	28.60	729.3901	731.3962	495.3318	2.0061	1.0E+05	1	1	1
4935	28.64	687.3440	689.3496	453.2857	2.0055	2.5E+04	1	1	2
4938	28.66	755.4057	757.4120	521.3473	2.0063	2.1E+04	1	1	2
4943	28.69	713.3597	715.3653	479.3014	2.0056	8.3E+05	1	1	4
4952	28.75	797.3171	799.3188	563.2588	2.0017	1.4E+04	1	1	1
4955	28.76	751.3729	753.3769	517.3145	2.0041	8.1E+03	1	1	2
4958	28.81	305.0952	307.1044	71.0369	2.0092	1.4E+04	1	1	1
4959	28.81	687.3441	689.3498	453.2857	2.0058	1.3E+04	1	1	2
4968	28.86	713.3596	715.3653	479.3013	2.0058	6.8E+05	1	1	4
4976	28.91	570.2897	572.2928	336.2314	2.0031	1.1E+04	1	1	22
4996	28.98	713.3596	715.3657	479.3013	2.0061	3.6E+05	1	1	4
4998	28.98	687.3442	689.3500	453.2859	2.0058	1.5E+04	1	1	2
5017	29.05	755.4057	757.4112	521.3474	2.0055	4.7E+04	1	1	2
5079	29.24	403.0625	405.0659	169.0042	2.0033	5.0E+04	1	1	1
5080	29.24	713.3598	715.3659	479.3015	2.0061	2.0E+05	1	1	4
5084	29.25	755.4060	757.4128	521.3477	2.0068	1.1E+04	1	1	2
5086	29.27	763.3728	765.3813	529.3145	2.0085	6.4E+03	1	1	2
5094	29.31	687.3430	689.3493	453.2847	2.0063	1.7E+04	1	1	2
5107	29.36	584.3052	586.3090	350.2469	2.0039	1.3E+04	1	1	5
5110	29.37	713.3595	715.3652	479.3012	2.0057	6.2E+05	1	1	4
5134	29.51	687.3451	689.3502	453.2868	2.0051	1.4E+04	1	1	2
5137	29.56	713.3597	715.3655	479.3014	2.0057	6.3E+05	1	1	4
5144	29.60	735.3421	737.3479	501.2838	2.0058	7.6E+03	1	1	4
5155	29.68	713.3588	715.3650	479.3005	2.0062	2.3E+05	1	1	4
5156	29.69	755.4072	757.4137	521.3489	2.0065	2.9E+04	1	1	2
5160	29.71	687.3446	689.3502	453.2862	2.0056	1.5E+04	1	1	2
5176	29.80	755.4062	757.4134	521.3479	2.0072	2.4E+04	1	1	2
5195	29.83	733.3305	735.3397	499.2722	2.0092	1.2E+04	1	1	2
5239	29.93	549.2991	551.3051	315.2408	2.0060	9.5E+03	1	1	1
5250	29.99	682.2661	684.2704	448.2078	2.0043	8.5E+03	1	1	5
5254	30.01	713.3598	715.3658	479.3014	2.0060	3.5E+05	1	1	4
5256	30.02	687.3441	689.3502	453.2858	2.0061	1.5E+04	1	1	2
5283	30.12	687.3447	689.3499	453.2863	2.0052	1.3E+04	1	1	2
5305	30.22	687.3459	689.3504	453.2876	2.0045	9.1E+03	1	1	2
5311	30.26	713.3591	715.3649	479.3007	2.0059	3.8E+05	1	1	4
5325	30.31	687.3438	689.3513	453.2855	2.0074	8.1E+03	1	1	2
5334	30.35	317.1077	319.1146	166.0987	2.0069	7.3E+03	2	2	1
5348	30.53	687.3442	689.3499	453.2859	2.0057	6.6E+03	1	1	2

5378	30.83	713.3593	715.3652	479.3010	2.0058	3.0E+05	1	1	4
5403	31.07	595.3196	597.3252	361.2613	2.0056	7.8E+03	1	1	1
5411	31.25	659.3115	661.3157	425.2532	2.0042	8.1E+03	1	1	2
5444	31.57	713.3595	715.3659	479.3012	2.0064	1.6E+05	1	1	4
5457	31.66	370.0958	372.1011	136.0375	2.0054	1.3E+04	1	1	2

Supplemental Table T3. Metabolites putatively identified using accurate mass search against the predicted human m

feature ID	rt (min)	mz_light	mz_heavy	Monoisotopic molecular mass	distance	int_heavy	nCharge	nTag	Matches
3	1.72	778.1809	780.1876	544.1226	2.0067	3.8E+04	1	1	2
6	1.84	585.2889	587.2900	351.2306	2.0011	3.9E+04	1	1	1
7	1.85	804.1741	806.1754	570.1158	2.0013	2.3E+04	1	1	1
8	1.85	357.0671	359.0732	123.0088	2.0061	2.2E+04	1	1	2
10	1.89	549.1002	551.1069	315.0419	2.0066	3.0E+04	1	1	3
11	1.90	782.1922	784.1942	548.1338	2.0021	2.9E+04	1	1	1
12	1.90	493.0774	495.0831	259.0191	2.0056	1.3E+04	1	1	2
15	1.93	629.3149	631.3163	395.2566	2.0015	2.5E+04	1	1	2
19	1.99	573.1306	575.1383	339.0723	2.0076	2.3E+04	1	1	4
20	2.00	589.1277	591.1322	355.0694	2.0046	9.6E+03	1	1	5
21	2.01	575.1385	577.1414	341.0802	2.0029	1.3E+04	1	1	4
22	2.02	387.0676	389.0740	153.0093	2.0064	1.1E+04	1	1	8
24	2.02	778.1803	780.1870	544.1220	2.0066	2.2E+04	1	1	2
27	2.04	717.3671	719.3694	483.3088	2.0023	4.4E+04	1	1	4
29	2.09	630.1514	632.1531	396.0931	2.0017	1.9E+04	1	1	3
30	2.10	652.1337	654.1440	418.0754	2.0103	1.1E+04	1	1	2
31	2.10	881.2130	883.2216	647.1546	2.0086	8.7E+03	1	1	4
33	2.10	782.1928	784.1931	548.1345	2.0003	1.3E+04	1	1	1
34	2.11	628.1451	630.1518	394.0867	2.0067	1.9E+04	1	1	10
35	2.11	419.0408	421.0475	184.9825	2.0067	1.3E+04	1	1	2
36	2.11	903.1953	905.2042	669.1370	2.0089	8.4E+03	1	1	2
38	2.12	672.1102	674.1159	438.0519	2.0058	7.3E+03	1	1	2
40	2.12	650.1275	652.1349	416.0692	2.0074	1.1E+04	1	1	2
42	2.14	474.1433	476.1500	240.0850	2.0067	9.2E+03	1	1	25
43	2.14	879.2079	881.2147	645.1496	2.0068	1.3E+04	1	1	1
45	2.16	667.1905	669.1959	433.1321	2.0055	6.3E+03	1	1	9
54	2.26	805.4190	807.4211	571.3607	2.0022	1.4E+04	1	1	1
56	2.27	595.1364	597.1424	361.0781	2.0060	1.0E+04	1	1	17
60	2.33	761.3941	763.3951	527.3358	2.0010	1.5E+04	1	1	10
61	2.33	389.1819	391.1928	310.2471	2.0109	6.5E+03	2	2	1
63	2.36	389.0918	391.0983	155.0334	2.0065	1.4E+04	1	1	4
64	2.36	565.1300	567.1335	331.0717	2.0036	1.6E+04	1	1	4
65	2.37	543.1487	545.1479	309.0904	1.9991	3.8E+04	1	1	1
68	2.39	645.0878	647.0868	411.0294	1.9990	2.2E+04	1	1	1
74	2.43	573.1304	575.1385	339.0721	2.0081	6.9E+03	1	1	4
75	2.45	403.0356	405.0423	168.9772	2.0068	2.0E+04	1	1	1
83	2.51	759.3982	761.3973	525.3399	1.9990	7.7E+03	1	1	5
86	2.53	803.4230	805.4201	569.3647	1.9971	6.4E+03	1	1	1
90	2.58	641.1757	643.1802	407.1174	2.0045	7.4E+03	1	1	9
93	2.59	388.1076	390.1137	154.0493	2.0062	3.4E+05	1	1	10
95	2.59	610.1322	612.1400	376.0739	2.0077	8.1E+03	1	1	1
99	2.61	721.1499	723.1497	487.0916	1.9997	6.2E+04	1	1	1
100	2.61	431.1029	433.1100	197.0445	2.0071	1.2E+04	1	1	12

101	2.62	748.1806	750.1844	514.1223	2.0038	1.1E+04	1	1	5
105	2.65	403.0356	405.0424	168.9773	2.0068	1.4E+04	1	1	1
109	2.70	719.1451	721.1512	485.0867	2.0062	1.8E+04	1	1	2
110	2.70	460.1638	462.1695	226.1054	2.0057	2.2E+04	1	1	1
125	2.87	667.1894	669.1976	433.1310	2.0082	7.8E+03	1	1	4
139	2.99	724.1908	726.1992	490.1325	2.0084	7.0E+03	1	1	3
147	3.02	470.1196	472.1220	236.0613	2.0024	1.8E+04	1	1	3
166	3.24	713.2567	715.2630	479.1984	2.0063	1.5E+04	1	1	5
172	3.29	711.2498	713.2557	477.1915	2.0058	8.8E+03	1	1	4
179	3.36	543.2734	545.2846	618.4301	2.0112	6.8E+03	2	2	4
180	3.36	535.1729	537.1784	301.1146	2.0054	7.6E+03	1	1	1
184	3.38	521.2618	523.2654	574.4070	2.0036	9.6E+03	2	2	2
189	3.41	347.0555	349.0631	225.9943	2.0076	8.7E+03	2	2	2
190	3.41	693.1028	697.1155	225.9935	4.0127	3.3E+04	1	2	2
191	3.41	693.1031	695.1040	459.0448	2.0008	4.8E+04	1	1	1
199	3.44	696.1121	698.1126	462.0538	2.0006	2.8E+04	1	1	1
206	3.51	514.3059	516.3106	560.4951	2.0048	2.0E+05	2	2	4
208	3.52	471.1428	473.1495	237.0845	2.0068	9.3E+03	1	1	26
211	3.54	383.0699	385.0785	149.0116	2.0085	7.4E+03	1	1	1
216	3.59	633.1944	635.2004	399.1361	2.0060	8.2E+03	1	1	20
225	3.62	514.3058	516.3093	560.4950	2.0035	1.7E+05	2	2	4
242	3.70	547.1493	549.1585	313.0910	2.0092	1.6E+04	1	1	6
243	3.71	547.1545	549.1599	313.0962	2.0055	4.1E+04	1	1	1
253	3.79	633.1942	635.2019	399.1358	2.0077	7.7E+03	1	1	20
271	3.94	544.2767	546.2819	620.4367	2.0052	6.5E+03	2	2	1
280	4.03	699.3768	701.3922	465.3185	2.0154	7.3E+03	1	1	3
286	4.15	536.3186	538.3255	302.2603	2.0069	1.3E+05	1	1	10
293	4.18	388.1073	390.1136	154.0490	2.0063	3.8E+04	1	1	10
294	4.18	743.4024	745.4174	509.3440	2.0151	8.9E+03	1	1	3
305	4.26	780.3936	782.3997	546.3353	2.0061	2.7E+04	1	1	2
316	4.31	737.2035	739.2105	503.1452	2.0070	1.7E+04	1	1	5
322	4.33	802.4063	804.4132	568.3480	2.0068	1.6E+04	1	1	3
329	4.36	780.3935	782.3999	546.3352	2.0064	1.2E+04	1	1	2
335	4.37	566.2896	568.2946	664.4627	2.0050	8.1E+03	2	2	1
340	4.39	741.2144	743.2177	507.1561	2.0033	1.5E+04	1	1	1
354	4.46	802.4062	804.4130	568.3479	2.0068	1.1E+04	1	1	3
356	4.46	514.3061	516.3124	560.4955	2.0063	6.7E+03	2	2	4
357	4.46	737.2032	739.2092	503.1449	2.0059	1.1E+04	1	1	5
367	4.51	735.1977	737.2035	501.1394	2.0057	8.4E+03	1	1	2
384	4.65	562.3443	564.3594	656.5719	2.0151	2.4E+04	2	2	45
386	4.67	580.3452	582.3507	692.5737	2.0055	5.2E+05	2	2	4
398	4.75	580.3454	582.3476	692.5742	2.0022	6.5E+05	2	2	4
401	4.77	553.1413	555.1473	319.0830	2.0060	1.4E+04	1	1	4
419	4.86	553.1420	555.1480	319.0836	2.0060	1.5E+04	1	1	4
422	4.86	580.3443	582.3511	346.2860	2.0068	2.8E+05	1	1	15
425	4.89	602.3577	604.3614	736.5989	2.0036	7.6E+05	2	2	8
433	4.96	569.3138	571.3299	335.2555	2.0160	2.6E+04	1	1	1
441	4.99	610.3151	612.3201	752.5137	2.0050	1.9E+04	2	2	37

443	5.00	549.3179	551.3345	315.2596	2.0165	2.3E+04	1	1	1
446	5.01	631.3254	633.3417	397.2670	2.0164	2.3E+04	1	1	1
450	5.02	602.3584	604.3632	736.6001	2.0049	7.2E+05	2	2	8
477	5.17	653.3380	655.3484	419.2797	2.0104	1.1E+04	1	1	1
481	5.19	632.3283	634.3328	796.5400	2.0044	2.3E+04	2	2	35
482	5.19	624.3711	626.3752	780.6256	2.0041	6.6E+05	2	2	7
491	5.29	654.3417	656.3465	840.5668	2.0047	2.3E+04	2	2	21
500	5.37	654.3411	656.3458	840.5656	2.0047	2.5E+04	2	2	21
510	5.41	749.2066	751.2144	515.1483	2.0077	1.6E+04	1	1	2
513	5.42	675.3512	677.3646	441.2928	2.0134	2.3E+04	1	1	1
517	5.44	676.3540	678.3594	884.5913	2.0054	2.9E+04	2	2	44
522	5.49	668.3972	670.3999	868.6778	2.0026	5.8E+05	2	2	5
526	5.50	593.3609	595.3604	359.3026	1.9995	2.5E+04	1	1	1
527	5.50	549.3313	551.3337	315.2730	2.0024	2.0E+04	1	1	1
532	5.51	701.3922	703.4045	467.3339	2.0122	1.7E+04	1	1	1
540	5.54	668.3976	670.4033	868.6786	2.0057	4.8E+05	2	2	6
553	5.58	698.3676	700.3716	928.6187	2.0040	3.1E+04	2	2	28
568	5.63	690.4103	692.4143	912.7039	2.0040	5.2E+05	2	2	1
584	5.68	637.3883	639.3908	403.3300	2.0025	2.9E+04	1	1	1
588	5.68	694.4245	696.4378	920.7323	2.0134	5.4E+04	2	2	3
591	5.70	720.3814	722.3856	972.6461	2.0042	2.9E+04	2	2	1
594	5.71	712.4232	714.4263	956.7298	2.0031	4.6E+05	2	2	1
607	5.75	492.1805	494.1858	258.1221	2.0053	6.1E+03	1	1	8
614	5.78	529.3210	531.3317	295.2627	2.0107	3.1E+04	1	1	1
615	5.78	593.3606	595.3626	359.3023	2.0020	2.5E+04	1	1	1
616	5.79	637.3884	639.3891	403.3301	2.0007	3.5E+04	1	1	1
626	5.82	742.3947	744.3983	1016.6727	2.0036	2.7E+04	2	2	14
634	5.85	738.4503	740.4641	1008.7840	2.0138	2.2E+04	2	2	5
638	5.85	513.2791	515.2807	279.2207	2.0016	8.8E+03	1	1	6
640	5.86	701.3975	703.4092	467.3392	2.0118	1.2E+04	1	1	1
647	5.88	764.4073	766.4101	1060.6979	2.0028	3.6E+04	2	2	27
651	5.90	509.2704	511.2738	275.2121	2.0034	7.7E+04	1	1	1
654	5.91	362.1644	364.1708	128.1061	2.0064	2.0E+04	1	1	2
657	5.92	764.9095	766.9136	1061.7023	2.0041	1.5E+04	2	2	19
670	5.94	756.4492	758.4508	1044.7818	2.0015	3.1E+05	2	2	4
675	5.97	937.0201	939.0358	1405.9235	2.0157	1.6E+04	2	2	1
676	5.97	778.4618	780.4675	1088.8070	2.0056	2.7E+05	2	2	3
678	5.98	786.4215	788.4246	1104.7264	2.0031	2.0E+04	2	2	17
692	6.07	808.4342	810.4403	1148.7518	2.0061	2.9E+04	2	2	2
696	6.08	337.1585	339.1651	103.1002	2.0066	5.3E+05	1	1	5
701	6.11	800.4760	802.4825	566.4177	2.0065	2.2E+05	1	1	1
705	6.13	830.4483	832.4510	1192.7799	2.0027	2.6E+04	2	2	5
712	6.16	553.2965	555.3000	319.2382	2.0034	5.9E+04	1	1	1
713	6.16	548.3264	550.3312	314.2681	2.0048	1.8E+05	1	1	1
722	6.21	563.3354	565.3422	658.5541	2.0068	1.4E+05	2	2	43
747	6.29	560.3395	562.3499	652.5623	2.0104	3.9E+04	2	2	12
760	6.31	874.4753	876.4795	1280.8339	2.0043	2.4E+04	2	2	6
774	6.34	896.4889	898.4898	662.4306	2.0009	2.5E+04	1	1	1

775	6.34	593.3550	595.3578	718.5933	2.0028	4.6E+04	2	2	2
839	6.41	918.5021	920.5087	1368.8877	2.0066	2.4E+04	2	2	3
846	6.42	593.3532	595.3632	718.5898	2.0100	2.3E+04	2	2	9
874	6.46	613.0373	615.0429	378.9789	2.0057	4.0E+04	1	1	1
876	6.46	941.0218	943.0215	1413.9269	1.9997	7.7E+03	2	2	4
897	6.48	940.5148	942.5179	1412.9130	2.0031	2.2E+04	2	2	4
916	6.50	932.5559	934.5579	698.4975	2.0021	3.1E+05	1	1	2
929	6.51	618.3781	620.3859	768.6396	2.0077	7.3E+03	2	2	3
933	6.52	622.0374	624.0429	387.9790	2.0055	1.6E+05	1	1	1
943	6.53	334.1222	336.1287	100.0639	2.0064	1.6E+05	1	1	3
955	6.54	641.3491	643.3532	814.5816	2.0041	4.3E+04	2	2	18
959	6.54	962.5279	964.5318	728.4696	2.0039	2.1E+04	1	1	1
963	6.54	638.0335	642.0523	170.9242	4.0188	1.8E+05	1	2	1
974	6.56	954.5680	956.5649	720.5097	1.9969	2.1E+05	1	1	16
976	6.56	616.0285	618.0391	381.9702	2.0106	2.8E+04	1	1	1
984	6.56	954.5687	956.5722	720.5104	2.0035	2.9E+05	1	1	17
1006	6.58	667.3817	669.3947	433.3234	2.0130	1.1E+04	1	1	2
1010	6.59	495.2765	497.2795	261.2181	2.0030	1.5E+04	1	1	1
1013	6.59	660.0333	662.0351	425.9750	2.0018	4.1E+04	1	1	1
1020	6.59	984.5423	986.5459	750.4839	2.0036	1.4E+04	1	1	11
1057	6.63	627.3821	629.3857	786.6476	2.0035	2.2E+04	2	2	12
1061	6.63	703.4121	705.4254	469.3538	2.0133	6.8E+03	1	1	3
1062	6.63	495.2895	497.2999	261.2311	2.0105	3.6E+04	1	1	2
1081	6.66	976.5822	978.5846	742.5239	2.0024	1.5E+05	1	1	28
1083	6.66	657.0639	659.0701	423.0056	2.0062	4.9E+04	1	1	1
1086	6.67	508.0597	510.0646	274.0013	2.0050	1.7E+04	1	1	2
1125	6.78	695.0798	697.0835	461.0215	2.0037	1.7E+05	1	1	1
1126	6.78	701.0896	703.0930	467.0313	2.0034	8.1E+04	1	1	1
1130	6.78	671.4079	673.4116	874.6991	2.0038	1.8E+04	2	2	1
1131	6.78	627.3808	629.3848	786.6450	2.0040	1.5E+04	2	2	12
1136	6.79	530.0732	532.0729	296.0149	1.9996	2.3E+04	1	1	2
1143	6.79	681.4018	683.4137	894.6870	2.0118	1.4E+04	2	2	8
1148	6.80	735.4104	737.4138	1002.7042	2.0034	1.0E+04	2	2	24
1149	6.81	637.3747	639.3860	806.6328	2.0113	1.1E+04	2	2	2
1159	6.82	733.4110	735.4138	499.3527	2.0028	5.7E+04	1	1	6
1165	6.83	556.0574	558.0611	643.9981	2.0037	2.1E+04	2	2	1
1167	6.83	541.0804	543.0779	307.0220	1.9975	4.6E+04	1	1	2
1193	6.85	734.4298	736.4427	500.3715	2.0129	1.7E+04	1	1	2
1198	6.86	733.4236	735.4228	499.3653	1.9992	4.5E+04	1	1	5
1205	6.87	552.0875	554.0832	636.0584	1.9957	5.8E+04	2	2	1
1206	6.87	552.0864	554.0870	318.0280	2.0006	3.8E+04	1	1	5
1227	6.89	527.3148	529.3205	586.5129	2.0057	6.3E+03	2	2	1
1229	6.89	748.4218	750.4329	514.3635	2.0111	3.5E+04	1	1	8
1232	6.90	560.0789	562.0816	326.0206	2.0027	9.4E+04	1	1	1
1233	6.90	811.4902	813.4947	577.4319	2.0045	2.9E+04	1	1	2
1243	6.90	763.0947	767.1041	295.9854	4.0093	1.6E+04	1	2	1
1250	6.91	730.1073	732.1085	496.0490	2.0012	2.6E+04	1	1	1
1253	6.91	682.0759	686.0832	214.9666	4.0073	7.3E+03	1	2	1

1270	6.92	747.4384	749.4388	1026.7601	2.0004	3.2E+04	2	2	1
1275	6.93	715.4347	717.4366	962.7527	2.0020	9.1E+03	2	2	3
1280	6.93	763.0976	765.1082	529.0393	2.0106	3.4E+04	1	1	1
1283	6.94	467.0563	469.0669	232.9980	2.0106	7.0E+03	1	1	1
1284	6.94	627.1792	629.1837	393.1208	2.0045	8.1E+03	1	1	16
1287	6.94	756.1120	760.1251	289.0026	4.0131	1.6E+04	1	2	7
1299	6.94	381.1478	383.1543	147.0895	2.0064	1.1E+04	1	1	39
1310	6.95	570.0948	572.0968	672.0730	2.0020	5.5E+04	2	2	1
1319	6.96	551.3295	553.3400	634.5425	2.0104	6.2E+03	2	2	1
1323	6.96	582.0900	586.1082	114.9807	4.0182	6.0E+04	1	2	1
1324	6.96	593.3474	595.3549	718.5782	2.0075	2.1E+04	2	2	16
1328	6.96	582.0923	584.0962	348.0340	2.0039	5.1E+04	1	1	1
1333	6.96	777.4406	779.4389	543.3823	1.9983	2.3E+04	1	1	1
1335	6.97	563.0942	565.0969	329.0359	2.0027	1.6E+04	1	1	1
1343	6.97	777.4380	779.4434	543.3797	2.0054	2.7E+04	1	1	2
1346	6.97	778.1062	780.1060	544.0479	1.9998	1.6E+04	1	1	2
1352	6.97	777.4400	779.4533	543.3817	2.0133	1.7E+04	1	1	1
1358	6.98	582.0958	584.0993	348.0374	2.0035	4.4E+04	1	1	7
1370	6.98	723.4384	725.4362	489.3800	1.9978	3.2E+04	1	1	1
1375	6.99	748.1086	752.1288	280.9993	4.0202	6.4E+03	1	2	1
1378	6.99	600.3376	602.3463	732.5585	2.0088	9.8E+03	2	2	6
1381	6.99	778.1073	780.1204	544.0490	2.0131	1.8E+04	1	1	2
1395	7.00	768.4601	770.4626	534.4018	2.0026	1.9E+04	1	1	2
1397	7.00	574.1007	576.1025	340.0424	2.0018	1.7E+04	1	1	1
1404	7.01	783.1342	787.1553	316.0249	4.0211	7.4E+03	1	2	2
1410	7.01	783.1317	785.1343	549.0734	2.0026	7.9E+03	1	1	4
1411	7.01	670.3278	672.3318	436.2695	2.0040	2.0E+04	1	1	1
1414	7.01	592.1078	594.1098	716.0989	2.0020	3.0E+04	2	2	1
1415	7.01	783.4664	785.4708	549.4081	2.0044	1.2E+04	1	1	2
1426	7.02	792.4474	794.4526	558.3891	2.0052	1.4E+04	1	1	6
1435	7.03	679.4118	681.4124	445.3535	2.0007	1.6E+04	1	1	2
1441	7.03	593.1014	595.1081	359.0431	2.0066	3.3E+04	1	1	1
1442	7.03	584.0978	586.1095	700.0789	2.0118	9.8E+03	2	2	1
1447	7.03	695.3193	697.3236	461.2610	2.0043	1.4E+04	1	1	1
1454	7.04	450.1682	452.1797	216.1099	2.0114	1.1E+04	1	1	15
1459	7.04	785.1385	789.1420	318.0291	4.0036	2.1E+04	1	2	5
1462	7.05	770.1286	774.1353	303.0193	4.0067	1.2E+04	1	2	1
1470	7.06	611.0915	613.0954	754.0663	2.0039	1.8E+04	2	2	3
1483	7.07	595.1036	597.1131	722.0905	2.0095	7.8E+03	2	2	2
1494	7.08	392.1636	394.1699	158.1053	2.0063	9.3E+03	1	1	6
1496	7.08	596.1128	598.1144	362.0545	2.0016	9.4E+03	1	1	5
1497	7.08	603.1148	605.1168	738.1130	2.0020	1.6E+04	2	2	2
1512	7.10	427.0983	429.1052	193.0400	2.0070	8.7E+03	1	1	18
1515	7.11	615.1205	617.1301	381.0621	2.0096	1.1E+04	1	1	1
1518	7.11	615.1136	619.1277	148.0043	4.0141	1.3E+04	1	2	1
1526	7.11	625.1997	627.2036	391.1413	2.0039	7.3E+03	1	1	5
1527	7.12	619.8721	621.8729	771.6275	2.0009	8.0E+03	2	2	2
1529	7.12	614.1206	616.1232	760.1245	2.0026	9.0E+03	2	2	5

1533	7.13	553.3304	555.3331	319.2721	2.0026	7.3E+03	1	1	2
1535	7.13	626.1173	630.1366	159.0080	4.0192	1.4E+04	1	2	1
1544	7.14	626.1296	628.1403	392.0712	2.0107	9.0E+03	1	1	37
1546	7.15	383.0730	385.0794	149.0147	2.0064	9.6E+03	1	1	6
1547	7.15	616.6156	618.6276	765.1146	2.0119	7.2E+03	2	2	4
1552	7.16	630.3735	632.3743	396.3152	2.0008	9.0E+03	1	1	1
1564	7.18	633.3827	635.3870	798.6488	2.0043	1.1E+04	2	2	29
1571	7.22	367.1317	369.1375	133.0733	2.0059	2.1E+04	1	1	40
1576	7.23	605.1589	607.1651	371.1006	2.0062	7.6E+03	1	1	5
1578	7.24	669.3228	671.3261	435.2645	2.0033	1.2E+04	1	1	15
1579	7.24	478.1634	480.1699	244.1051	2.0065	9.1E+03	1	1	12
1590	7.30	606.3574	608.3628	372.2991	2.0055	7.2E+03	1	1	1
1596	7.31	677.4094	679.4118	886.7021	2.0025	8.4E+03	2	2	9
1599	7.35	358.0416	360.0477	123.9833	2.0061	1.9E+04	1	1	3
1611	7.44	378.1483	380.1540	144.0900	2.0057	8.0E+03	1	1	17
1613	7.48	625.1625	627.1667	391.1042	2.0042	1.5E+04	1	1	13
1614	7.48	359.1059	361.1125	125.0476	2.0066	1.4E+04	1	1	15
1615	7.50	437.0943	439.0987	203.0360	2.0044	1.1E+04	1	1	2
1622	7.61	587.1299	589.1339	353.0716	2.0040	8.1E+03	1	1	2
1631	7.82	452.1842	454.1903	218.1259	2.0061	1.7E+04	1	1	4
1632	7.83	453.1317	455.1387	219.0734	2.0070	1.0E+04	1	1	17
1633	7.86	623.1555	625.1617	389.0972	2.0062	1.2E+04	1	1	1
1645	8.02	355.0922	357.0987	242.0678	2.0065	1.6E+04	2	2	2
1651	8.09	737.1916	739.2033	503.1333	2.0117	6.8E+03	1	1	13
1654	8.10	657.3943	659.3960	846.6720	2.0017	6.3E+03	2	2	5
1655	8.10	739.2036	741.2042	505.1453	2.0007	1.4E+04	1	1	4
1658	8.10	736.1933	740.2058	269.0839	4.0125	9.6E+03	1	2	1
1671	8.24	420.1582	422.1650	186.0998	2.0069	9.5E+03	1	1	15
1678	8.28	635.2398	637.2413	401.1815	2.0015	8.6E+03	1	1	4
1684	8.28	451.1049	453.1094	434.0931	2.0045	1.4E+04	2	2	8
1685	8.28	489.1214	491.1263	255.0630	2.0049	6.2E+03	1	1	3
1694	8.29	601.1473	603.1514	367.0889	2.0041	8.8E+03	1	1	7
1697	8.29	559.1093	561.1137	325.0510	2.0044	7.2E+03	1	1	1
1708	8.31	540.1349	542.1414	306.0765	2.0065	6.1E+05	1	1	1
1717	8.32	639.3851	641.3879	405.3268	2.0028	7.2E+03	1	1	1
1719	8.32	567.0953	569.0987	333.0370	2.0034	1.3E+04	1	1	5
1723	8.32	661.1576	663.1561	427.0992	1.9985	1.7E+05	1	1	4
1727	8.33	695.3338	697.3415	461.2755	2.0077	1.2E+04	1	1	3
1730	8.33	645.2454	647.2466	411.1871	2.0012	9.7E+03	1	1	6
1733	8.34	659.1510	661.1576	425.0927	2.0066	8.5E+04	1	1	4
1735	8.34	584.1187	586.1249	350.0604	2.0062	7.7E+03	1	1	2
1745	8.36	664.2584	666.2596	430.2000	2.0012	1.1E+04	1	1	6
1749	8.37	571.2038	575.2181	104.0945	4.0143	7.1E+03	1	2	2
1750	8.38	583.6037	585.6047	699.0907	2.0010	7.4E+03	2	2	4
1751	8.38	664.1144	666.1165	430.0561	2.0021	1.1E+04	1	1	6
1752	8.38	667.1172	669.1177	433.0589	2.0005	6.2E+03	1	1	1
1756	8.39	654.3956	656.3960	420.3373	2.0003	6.4E+03	1	1	6
1758	8.39	673.1207	675.1227	439.0624	2.0019	9.0E+03	1	1	7

1759	8.39	677.2653	679.2669	443.2070	2.0016	6.2E+03	1	1	3
1760	8.40	664.4003	666.4025	430.3420	2.0022	8.6E+03	1	1	4
1765	8.40	682.3286	684.3307	896.5405	2.0022	9.2E+03	2	2	12
1767	8.40	658.2537	660.2551	424.1954	2.0014	7.3E+03	1	1	8
1776	8.43	589.1053	591.1077	710.0940	2.0023	7.8E+03	2	2	3
1782	8.44	677.1225	681.1303	210.0131	4.0078	9.5E+03	1	2	1
1784	8.44	683.4108	685.4131	449.3525	2.0022	9.6E+03	1	1	9
1789	8.45	605.7412	607.7418	743.3657	2.0007	8.0E+03	2	2	1
1793	8.45	685.3384	687.3374	451.2801	1.9990	2.8E+04	1	1	1
1796	8.45	597.7340	599.7389	727.3513	2.0050	6.7E+03	2	2	3
1798	8.46	683.2688	685.2703	449.2104	2.0015	9.1E+03	1	1	2
1800	8.47	692.4175	694.4191	916.7183	2.0016	6.5E+03	2	2	9
1805	8.47	677.1225	679.1228	443.0641	2.0003	6.7E+03	1	1	2
1806	8.48	600.1137	602.1156	732.1108	2.0019	6.3E+03	2	2	3
1812	8.48	614.2448	616.2480	380.1865	2.0031	7.2E+03	1	1	2
1817	8.49	702.1375	704.1398	468.0792	2.0023	7.7E+03	1	1	4
1820	8.50	616.7491	618.7502	765.3816	2.0011	7.1E+03	2	2	2
1822	8.50	512.1249	514.1312	278.0665	2.0064	1.8E+04	1	1	1
1823	8.50	512.6239	514.6302	557.1311	2.0063	1.7E+04	2	2	2
1827	8.50	609.1169	611.1193	750.1172	2.0023	6.1E+03	2	2	1
1832	8.51	708.4283	710.4297	474.3699	2.0015	9.8E+03	1	1	5
1837	8.52	616.6213	618.6247	765.1260	2.0034	8.7E+03	2	2	1
1847	8.53	633.3814	635.3846	798.6461	2.0032	7.0E+03	2	2	12
1854	8.54	622.2522	624.2544	776.3877	2.0022	6.1E+03	2	2	4
1870	8.56	630.8808	632.8809	793.6449	2.0001	9.3E+03	2	2	14
1875	8.56	644.2632	646.2647	820.4099	2.0014	6.2E+03	2	2	3
1876	8.56	644.3912	646.3886	820.6658	1.9974	7.0E+03	2	2	8
1899	8.62	652.8917	654.8929	837.6669	2.0011	9.7E+03	2	2	14
1900	8.62	493.2112	495.2167	259.1529	2.0055	7.3E+03	1	1	4
1904	8.64	320.1063	322.1115	86.0480	2.0052	3.0E+04	1	1	5
1914	8.69	674.9066	676.9125	881.6966	2.0059	6.3E+03	2	2	2
1915	8.69	376.1696	378.1754	142.1113	2.0057	9.4E+03	1	1	5
1918	8.76	680.0716	682.0747	446.0133	2.0031	7.0E+03	1	1	3
1923	8.81	366.1017	368.1080	132.0434	2.0063	7.7E+03	1	1	4
1931	8.88	969.2027	971.2069	735.1443	2.0043	8.8E+03	1	1	1
1932	8.88	466.1995	468.2071	232.1412	2.0076	8.1E+03	1	1	9
1933	8.89	420.1582	422.1651	186.0999	2.0068	1.0E+04	1	1	15
1934	8.89	947.2202	949.2222	713.1619	2.0020	4.6E+04	1	1	1
1936	8.91	496.0956	498.0927	262.0373	1.9971	6.2E+04	1	1	1
1938	8.93	434.1151	436.1217	400.1135	2.0066	6.6E+03	2	2	15
1942	8.96	924.2434	926.2435	690.1851	2.0001	1.9E+04	1	1	1
1947	9.05	367.1320	369.1381	133.0737	2.0061	2.7E+04	1	1	40
1958	9.25	489.1326	491.1347	255.0743	2.0022	2.1E+04	1	1	9
1959	9.27	278.0956	280.1032	44.0373	2.0076	9.1E+03	1	1	1
1960	9.28	602.1301	604.1351	368.0718	2.0049	9.8E+03	1	1	3
1961	9.28	543.2092	545.2108	309.1509	2.0016	7.6E+03	1	1	1
1963	9.33	485.1208	487.1252	251.0624	2.0045	1.1E+04	1	1	6
1964	9.35	440.1306	442.1343	206.0722	2.0038	1.1E+04	1	1	19

1966	9.41	466.2000	468.2091	232.1417	2.0091	1.1E+04	1	1	9
1971	9.49	357.0889	359.0955	123.0305	2.0066	1.2E+04	1	1	1
1980	9.58	531.1298	533.1354	594.1429	2.0056	1.8E+04	2	2	1
1981	9.58	341.5870	343.5934	215.0574	2.0064	9.8E+03	2	2	2
1992	9.60	476.1150	478.1211	242.0567	2.0061	1.6E+04	1	1	6
1998	9.63	476.6130	478.6185	485.1093	2.0055	1.2E+04	2	2	7
2001	9.76	414.5979	416.6043	361.0792	2.0064	1.3E+04	2	2	16
2004	9.80	785.1847	787.1843	551.1264	1.9996	3.0E+04	1	1	9
2005	9.80	783.1785	785.1838	549.1202	2.0054	4.1E+04	1	1	1
2006	9.81	711.1326	713.1352	477.0742	2.0026	1.5E+04	1	1	1
2007	9.81	355.0637	357.0693	242.0108	2.0055	7.1E+03	2	2	7
2011	9.85	714.3312	716.3408	480.2729	2.0096	6.4E+03	1	1	22
2016	9.88	367.1070	369.1106	133.0487	2.0036	1.4E+04	1	1	1
2022	9.97	795.2759	797.2787	561.2176	2.0029	1.6E+04	1	1	1
2024	9.97	292.1112	294.1162	58.0528	2.0051	2.9E+04	1	1	1
2030	9.99	711.1323	713.1363	477.0740	2.0040	8.9E+03	1	1	1
2032	10.02	397.1348	399.1418	326.1529	2.0070	7.7E+03	2	2	3
2034	10.07	399.5879	401.5950	331.0592	2.0070	6.3E+03	2	2	2
2035	10.07	436.1897	438.1950	202.1314	2.0053	1.1E+04	1	1	14
2037	10.10	379.5836	381.5902	291.0506	2.0066	8.1E+03	2	2	3
2040	10.15	296.0953	298.1014	62.0370	2.0061	4.7E+04	1	1	19
2041	10.16	439.0989	441.1043	205.0406	2.0054	1.5E+04	1	1	18
2044	10.24	340.1215	342.1282	106.0632	2.0067	2.0E+04	1	1	19
2045	10.25	362.1532	364.1595	128.0948	2.0063	5.7E+04	1	1	8
2046	10.26	486.1697	488.1760	252.1114	2.0062	1.0E+04	1	1	9
2049	10.29	458.1191	460.1213	224.0608	2.0022	2.4E+04	1	1	1
2051	10.31	514.1656	516.1768	280.1073	2.0112	6.8E+03	1	1	4
2052	10.31	446.0936	448.1017	424.0705	2.0081	6.4E+03	2	2	3
2055	10.33	381.1474	383.1545	147.0891	2.0071	9.1E+03	1	1	39
2059	10.34	424.0994	426.1057	190.0411	2.0063	6.3E+04	1	1	2
2060	10.34	727.2555	729.2611	493.1972	2.0056	6.9E+03	1	1	2
2063	10.34	581.1188	583.1212	347.0605	2.0024	1.2E+04	1	1	9
2069	10.38	899.2100	901.2090	665.1517	1.9990	2.7E+04	1	1	1
2071	10.38	410.5865	412.5933	353.0564	2.0067	1.2E+04	2	2	1
2073	10.39	409.5867	411.5932	351.0567	2.0065	3.6E+04	2	2	1
2075	10.39	448.1026	450.1090	428.0885	2.0064	2.6E+05	2	2	1
2077	10.39	384.1478	386.1543	150.0894	2.0065	3.3E+04	1	1	16
2078	10.41	897.2111	899.2110	663.1528	1.9998	4.1E+04	1	1	2
2081	10.47	554.1311	556.1381	320.0728	2.0071	2.6E+04	1	1	4
2096	10.56	697.3207	701.3337	230.2114	4.0130	1.2E+04	1	2	2
2097	10.57	530.1084	532.1133	296.0501	2.0049	9.8E+03	1	1	6
2099	10.58	466.2002	468.2058	232.1418	2.0057	1.5E+04	1	1	9
2105	10.65	711.3360	713.3382	477.2776	2.0022	2.5E+04	1	1	5
2108	10.68	420.1117	422.1182	372.1067	2.0065	6.3E+03	2	2	5
2110	10.68	658.2377	660.2365	424.1794	1.9988	8.6E+03	1	1	1
2121	10.75	436.1894	438.1951	202.1310	2.0057	1.1E+04	1	1	14
2127	10.83	785.1850	787.1851	551.1267	2.0001	2.4E+04	1	1	9
2128	10.83	783.1785	785.1837	549.1201	2.0053	2.8E+04	1	1	1

2131	10.87	495.0531	497.0557	260.9947	2.0026	2.4E+04	1	1	2
2132	10.88	318.1271	320.1329	84.0688	2.0057	4.4E+04	1	1	2
2135	10.90	441.1145	443.1209	207.0562	2.0063	1.8E+04	1	1	22
2145	11.00	418.5910	420.5979	369.0653	2.0070	7.9E+04	2	2	3
2153	11.03	419.5908	421.5980	371.0650	2.0072	1.2E+04	2	2	2
2157	11.06	886.3586	888.3650	652.3003	2.0064	1.7E+04	1	1	1
2160	11.16	565.2695	567.2751	331.2112	2.0056	2.1E+04	1	1	3
2169	11.18	563.2623	565.2693	329.2040	2.0070	1.2E+04	1	1	6
2182	11.21	335.1428	337.1495	101.0845	2.0067	1.2E+04	1	1	20
2190	11.24	626.2628	628.2677	392.2045	2.0049	6.3E+03	1	1	4
2192	11.26	606.2857	608.2889	372.2274	2.0032	5.0E+04	1	1	1
2196	11.29	736.1932	740.2054	269.0839	4.0122	9.7E+03	1	2	1
2208	11.31	427.1197	429.1267	386.1227	2.0070	7.4E+03	2	2	5
2210	11.31	388.1376	390.1448	308.1585	2.0072	9.1E+03	2	2	12
2213	11.31	426.1176	428.1254	384.1186	2.0077	2.9E+04	2	2	13
2227	11.35	340.1217	342.1272	106.0633	2.0055	1.8E+04	1	1	19
2231	11.37	665.3315	667.3379	431.2732	2.0064	4.3E+04	1	1	1
2235	11.38	711.3657	713.3679	477.3073	2.0022	1.1E+05	1	1	12
2238	11.42	753.3836	755.3878	519.3253	2.0042	6.1E+05	1	1	2
2243	11.47	709.3571	711.3637	475.2987	2.0067	8.6E+04	1	1	4
2250	11.53	892.4242	894.4270	658.3659	2.0027	9.0E+04	1	1	2
2253	11.55	335.1427	337.1501	101.0844	2.0074	8.7E+03	1	1	20
2255	11.56	887.4700	889.4733	653.4116	2.0033	1.1E+05	1	1	2
2258	11.58	560.2521	562.2561	326.1938	2.0040	5.1E+04	1	1	3
2261	11.61	384.1469	386.1512	150.0885	2.0043	2.3E+04	1	1	16
2268	11.62	621.1583	625.1717	154.0490	4.0134	4.4E+04	1	2	10
2271	11.63	311.0827	313.0894	154.0487	2.0068	5.9E+04	2	2	10
2282	11.65	846.3887	848.3944	612.3304	2.0057	1.8E+04	1	1	1
2284	11.65	499.2551	501.2621	530.3935	2.0071	7.2E+03	2	2	2
2323	11.70	587.3060	589.3134	706.4954	2.0074	9.7E+03	2	2	58
2324	11.70	346.0868	348.0931	112.0285	2.0063	8.1E+05	1	1	1
2330	11.70	565.2947	567.3030	662.4728	2.0083	1.1E+04	2	2	1
2344	11.71	621.1584	625.1708	154.0490	4.0124	3.4E+04	1	2	10
2356	11.72	609.3179	611.3264	750.5191	2.0085	1.2E+04	2	2	94
2359	11.72	631.3322	633.3409	397.2738	2.0088	1.3E+04	1	1	3
2369	11.74	653.3462	655.3537	838.5759	2.0075	1.9E+04	2	2	5
2377	11.74	524.2802	526.2910	290.2219	2.0108	2.4E+04	1	1	2
2392	11.76	697.3718	699.3795	463.3135	2.0077	2.9E+04	1	1	1
2394	11.76	436.1900	438.1959	202.1317	2.0059	1.1E+04	1	1	14
2417	11.77	764.4144	766.4218	530.3561	2.0074	1.4E+04	1	1	4
2421	11.78	786.4278	788.4341	552.3694	2.0063	1.5E+04	1	1	2
2422	11.78	808.4404	810.4472	574.3821	2.0068	1.6E+04	1	1	3
2430	11.79	697.8737	699.8812	927.6308	2.0075	2.0E+04	2	2	12
2431	11.79	785.4241	787.4294	551.3658	2.0053	4.1E+04	1	1	2
2444	11.80	852.4669	854.4727	618.4085	2.0058	1.7E+04	1	1	1
2446	11.80	829.9516	831.9581	595.8933	2.0065	3.0E+04	1	1	1
2447	11.81	914.4685	916.4822	680.4102	2.0137	1.1E+04	1	1	2
2449	11.81	452.2467	454.2533	218.1884	2.0066	2.6E+04	1	1	4

2458	11.81	830.4532	832.4595	596.3948	2.0064	1.5E+04	1	1	7
2459	11.81	719.3858	721.3927	485.3275	2.0069	3.4E+04	1	1	18
2467	11.82	874.4789	876.4842	640.4205	2.0053	1.7E+04	1	1	1
2473	11.82	958.4948	960.5044	724.4364	2.0096	1.3E+04	1	1	7
2480	11.83	741.3985	743.4056	507.3402	2.0072	3.8E+04	1	1	6
2482	11.83	895.9915	897.9957	661.9332	2.0042	2.9E+04	1	1	1
2483	11.83	896.4924	898.4971	662.4341	2.0047	1.6E+04	1	1	1
2489	11.84	918.5057	920.5097	684.4474	2.0040	1.5E+04	1	1	3
2490	11.84	763.4115	765.4181	529.3532	2.0065	3.9E+04	1	1	10
2494	11.84	563.2621	565.2679	329.2038	2.0057	1.3E+04	1	1	6
2497	11.85	940.5190	942.5186	706.4607	1.9996	1.4E+04	1	1	4
2501	11.85	744.4099	746.4223	1020.7031	2.0124	1.4E+04	2	2	10
2507	11.86	962.5311	964.5345	1456.9456	2.0034	1.4E+04	2	2	1
2510	11.86	496.2724	498.2817	262.2141	2.0093	6.8E+04	1	1	3
2513	11.86	807.4372	809.4441	573.3789	2.0069	4.1E+04	1	1	7
2521	11.87	607.2887	609.2989	373.2304	2.0101	1.1E+04	1	1	5
2522	11.87	956.4882	958.4945	722.4298	2.0064	1.9E+04	1	1	1
2532	11.87	870.4427	872.4463	636.3844	2.0037	2.1E+04	1	1	2
2535	11.87	912.4624	914.4693	678.4041	2.0069	2.5E+04	1	1	1
2537	11.87	851.4641	853.4688	617.4057	2.0048	3.8E+04	1	1	2
2538	11.88	514.2862	516.2977	280.2279	2.0115	1.4E+04	1	1	1
2561	11.89	983.5444	985.5476	749.4861	2.0032	2.1E+04	1	1	13
2570	11.90	603.3383	605.3439	738.5600	2.0056	8.7E+03	2	2	46
2572	11.91	520.2943	522.2985	572.4720	2.0041	2.3E+04	2	2	1
2573	11.91	895.4908	897.4968	661.4325	2.0060	3.5E+04	1	1	10
2585	11.91	384.1470	386.1536	150.0887	2.0066	1.8E+04	1	1	16
2612	11.94	577.3115	579.3204	343.2532	2.0089	1.9E+04	1	1	1
2613	11.94	592.5871	594.5997	717.0575	2.0126	8.9E+03	2	2	3
2625	11.95	549.3133	551.3142	630.5100	2.0008	1.2E+04	2	2	1
2632	11.96	564.3203	566.3258	660.5239	2.0055	9.7E+03	2	2	3
2634	11.96	505.2860	507.2875	542.4553	2.0016	1.8E+04	2	2	5
2637	11.96	788.4350	790.4456	1108.7533	2.0106	7.0E+03	2	2	15
2645	11.96	722.3998	724.4023	976.6830	2.0025	1.4E+04	2	2	3
2656	11.98	562.3107	564.3207	656.5047	2.0100	1.5E+04	2	2	66
2659	11.98	729.0721	731.0778	495.0138	2.0058	1.2E+04	1	1	1
2665	11.98	661.3720	663.3749	427.3137	2.0029	1.0E+04	1	1	1
2675	11.99	720.0715	722.0780	486.0132	2.0064	9.3E+03	1	1	1
2679	11.99	760.4296	762.4422	526.3713	2.0126	7.6E+03	1	1	1
2694	12.03	562.0678	564.0708	328.0095	2.0029	9.5E+03	1	1	2
2698	12.16	456.1581	458.1637	222.0997	2.0056	8.4E+03	1	1	23
2699	12.20	332.1428	334.1497	98.0845	2.0069	3.1E+04	1	1	1
2705	12.25	420.1582	422.1648	186.0999	2.0066	9.6E+03	1	1	15
2708	12.29	355.1636	357.1700	242.2106	2.0064	2.4E+04	2	2	1
2709	12.30	371.1405	373.1462	274.1643	2.0057	7.7E+03	2	2	2
2715	12.42	569.2171	571.2218	335.1588	2.0047	5.9E+04	1	1	16
2720	12.54	470.1737	472.1812	236.1154	2.0075	9.8E+03	1	1	11
2721	12.57	367.0785	369.0849	133.0202	2.0063	2.4E+06	1	1	4
2739	12.75	473.0861	475.0915	239.0278	2.0054	5.0E+04	1	1	2

2746	12.86	371.1405	373.1473	274.1644	2.0068	9.5E+03	2	2	2
2753	12.95	400.1398	402.1486	332.1630	2.0088	1.4E+04	2	2	6
2767	13.05	369.0976	371.1071	270.0786	2.0095	7.1E+03	2	2	1
2770	13.07	736.1929	740.2068	269.0835	4.0139	6.2E+03	1	2	1
2774	13.10	847.1922	851.2059	380.0829	4.0137	1.4E+04	1	2	2
2783	13.31	461.0989	463.1055	454.0812	2.0066	2.6E+04	2	2	3
2786	13.33	425.1071	427.1129	382.0975	2.0058	7.7E+03	2	2	2
2788	13.33	462.0995	464.1059	456.0824	2.0064	9.4E+03	2	2	1
2791	13.34	393.1348	395.1408	318.1530	2.0060	8.3E+03	2	2	5
2804	13.53	683.3213	685.3228	449.2629	2.0016	1.3E+04	1	1	2
2807	13.56	681.3102	683.3221	447.2519	2.0118	1.5E+04	1	1	1
2816	13.62	351.1015	353.1085	117.0432	2.0070	2.7E+05	1	1	1
2820	13.65	312.6370	314.6439	157.1573	2.0070	3.8E+04	2	2	1
2824	13.68	434.1734	436.1794	200.1151	2.0060	1.1E+04	1	1	11
2825	13.70	491.1120	493.1176	257.0537	2.0055	2.1E+04	1	1	2
2830	13.85	596.0822	598.0883	362.0239	2.0061	2.6E+04	1	1	3
2843	13.96	391.6002	393.6063	315.0838	2.0061	6.9E+03	2	2	7
2844	13.96	390.6004	392.6071	313.0841	2.0068	2.6E+04	2	2	11
2847	13.97	350.6272	352.6343	233.1378	2.0071	2.9E+04	2	2	1
2861	14.04	751.2039	755.2170	284.0945	4.0132	1.3E+04	1	2	1
2869	14.08	346.0926	348.0972	224.0686	2.0046	1.2E+04	2	2	12
2870	14.09	729.2445	733.2589	262.1351	4.0144	9.0E+03	1	2	2
2895	14.17	365.6235	367.6305	263.1305	2.0069	7.2E+03	2	2	6
2897	14.18	464.2202	466.2274	230.1619	2.0071	7.9E+03	1	1	4
2899	14.19	363.6174	365.6245	259.1181	2.0072	6.3E+03	2	2	11
2902	14.21	439.0986	441.1004	205.0403	2.0018	2.3E+04	1	1	18
2916	14.25	622.1612	626.1754	155.0518	4.0143	7.0E+03	1	2	1
2925	14.31	360.0902	362.0966	126.0319	2.0064	1.8E+04	1	1	15
2933	14.36	311.0826	313.0892	154.0486	2.0065	8.3E+04	2	2	10
2935	14.36	457.0645	459.0710	446.0124	2.0065	1.2E+04	2	2	3
2938	14.37	357.6353	359.6421	247.1540	2.0067	8.7E+03	2	2	2
2950	14.44	711.1454	713.1463	477.0871	2.0009	1.8E+04	1	1	6
2960	14.49	913.1219	917.1354	446.0125	4.0135	6.5E+03	1	2	3
2961	14.49	621.1575	625.1712	154.0481	4.0137	2.8E+04	1	2	2
2971	14.56	381.0937	383.1003	147.0354	2.0067	1.8E+04	1	1	4
2975	14.58	621.1581	625.1711	154.0487	4.0131	1.1E+04	1	2	10
2979	14.62	804.3203	806.3264	570.2620	2.0061	1.8E+04	1	1	1
2984	14.72	457.0654	459.0699	446.0142	2.0045	1.4E+04	2	2	3
2987	14.73	703.1674	705.1690	469.1091	2.0015	9.8E+03	1	1	4
2992	14.75	708.1878	710.1878	474.1295	2.0000	1.3E+04	1	1	2
2994	14.75	599.1208	601.1219	365.0625	2.0011	1.3E+04	1	1	7
2995	14.77	352.0895	354.0936	236.0623	2.0042	8.8E+03	2	2	3
2998	14.80	597.1139	599.1196	363.0556	2.0056	1.8E+04	1	1	1
2999	14.81	357.6350	359.6414	247.1534	2.0064	8.7E+03	2	2	2
3003	15.00	754.2036	758.2168	287.0943	4.0131	1.1E+05	1	2	4
3005	15.01	725.2059	729.2197	258.0966	4.0138	1.1E+04	1	2	15
3011	15.03	388.1074	390.1141	154.0491	2.0067	3.0E+05	1	1	10
3012	15.03	377.1038	379.1102	286.0910	2.0064	2.4E+04	2	2	8

3014	15.04	378.1068	380.1106	288.0969	2.0038	8.2E+03	2	2	30
3016	15.06	754.2026	756.2077	520.1443	2.0051	5.1E+04	1	1	5
3019	15.06	318.0794	320.0862	84.0211	2.0068	4.4E+04	1	1	13
3020	15.10	349.1584	351.1648	115.1000	2.0065	8.1E+03	1	1	21
3023	15.13	498.2054	500.2101	264.1471	2.0047	7.1E+03	1	1	9
3024	15.16	335.6220	337.6291	203.1273	2.0072	6.2E+03	2	2	11
3025	15.16	754.2031	758.2175	287.0938	4.0144	2.5E+04	1	2	4
3031	15.28	377.1031	379.1099	286.0896	2.0068	7.2E+03	2	2	1
3034	15.30	377.1775	379.1839	286.2384	2.0064	5.7E+04	2	2	1
3040	15.33	776.3294	780.3436	309.2200	4.0142	2.7E+04	1	2	1
3046	15.35	439.0987	441.1053	205.0404	2.0066	1.1E+04	1	1	18
3047	15.38	349.1582	351.1646	115.0999	2.0064	7.2E+03	1	1	21
3049	15.40	377.1761	379.1825	143.1178	2.0063	3.2E+05	1	1	1
3050	15.42	779.3409	781.3416	545.2826	2.0007	1.8E+04	1	1	1
3056	15.50	776.3289	780.3418	309.2195	4.0129	3.6E+04	1	2	1
3060	15.55	384.1484	386.1552	150.0900	2.0069	1.6E+04	1	1	1
3062	15.59	478.2359	480.2427	244.1775	2.0068	1.7E+04	1	1	1
3064	15.63	318.0796	320.0867	84.0213	2.0070	1.1E+04	1	1	13
3066	15.63	736.1923	740.2051	269.0829	4.0128	5.6E+04	1	2	1
3067	15.64	349.1579	351.1650	115.0995	2.0071	8.9E+03	1	1	21
3085	15.75	501.2054	503.2105	267.1471	2.0051	1.1E+04	1	1	9
3086	15.76	736.1924	740.2059	269.0831	4.0135	1.2E+04	1	2	1
3089	15.76	335.6221	337.6287	203.1276	2.0066	5.3E+04	2	2	11
3090	15.77	349.1583	351.1647	115.1000	2.0064	7.0E+03	1	1	21
3091	15.79	336.1193	338.1259	204.1219	2.0067	1.8E+04	2	2	4
3099	15.93	311.0822	313.0896	154.0479	2.0074	9.6E+03	2	2	2
3101	15.95	664.1955	666.1961	430.1372	2.0006	2.0E+04	1	1	3
3103	15.96	370.0567	372.0635	271.9967	2.0068	7.8E+03	2	2	1
3107	16.01	604.2216	606.2281	370.1633	2.0065	9.0E+03	1	1	3
3109	16.03	334.0741	336.0806	100.0158	2.0065	3.4E+04	1	1	16
3118	16.22	604.2195	606.2257	370.1612	2.0062	8.4E+03	1	1	2
3121	16.24	393.1499	395.1530	159.0916	2.0031	1.1E+04	1	1	1
3122	16.25	478.2366	480.2401	244.1782	2.0036	1.6E+04	1	1	1
3135	16.43	466.1429	468.1493	232.0846	2.0064	6.9E+03	1	1	9
3137	16.48	679.1411	681.1403	445.0827	1.9993	1.6E+04	1	1	2
3145	16.62	346.0660	348.0725	224.0154	2.0065	1.3E+04	2	2	3
3159	16.81	977.4105	981.4264	510.3012	4.0158	1.0E+04	1	2	2
3162	16.85	363.6537	365.6606	259.1908	2.0069	1.1E+04	2	2	3
3165	16.90	346.5960	348.6028	225.0753	2.0068	8.2E+03	2	2	20
3169	16.97	307.1476	309.1536	73.0892	2.0060	1.4E+04	1	1	8
3175	17.08	343.1214	345.1286	218.1262	2.0071	9.3E+03	2	2	4
3176	17.08	738.3205	740.3278	504.2622	2.0072	8.9E+03	1	1	4
3177	17.13	440.1737	442.1789	206.1154	2.0052	3.7E+04	1	1	5
3178	17.13	509.1776	511.1824	275.1193	2.0048	9.4E+03	1	1	4
3218	17.65	644.2236	646.2239	410.1652	2.0004	1.7E+04	1	1	1
3222	17.66	300.0799	302.0867	132.0432	2.0067	2.6E+04	2	2	4
3228	17.69	735.2087	739.2218	268.0994	4.0131	1.7E+05	1	2	2
3230	17.69	735.2076	737.2134	501.1493	2.0059	4.8E+04	1	1	5

3232	17.70	318.0794	320.0861	84.0210	2.0068	1.6E+04	1	1	13
3236	17.72	388.1070	390.1134	154.0487	2.0064	1.0E+04	1	1	10
3237	17.73	438.1573	440.1646	408.1981	2.0073	6.3E+03	2	2	3
3241	17.75	792.2607	796.2731	325.1514	4.0124	6.3E+03	1	2	9
3242	17.76	348.0904	350.0968	114.0321	2.0064	1.4E+04	1	1	28
3243	17.76	712.1818	716.1980	245.0724	4.0162	6.9E+03	1	2	6
3254	17.80	735.2061	737.2142	501.1478	2.0080	8.6E+03	1	1	3
3294	18.04	350.1300	352.1367	232.1433	2.0067	1.5E+04	2	2	9
3300	18.11	355.6370	357.6435	243.1575	2.0065	1.2E+04	2	2	2
3301	18.12	741.3880	743.3929	507.3296	2.0049	1.5E+04	1	1	5
3305	18.15	334.1582	336.1643	100.0999	2.0061	1.9E+04	1	1	2
3307	18.27	334.1583	336.1619	100.0999	2.0037	4.6E+04	1	1	2
3309	18.29	904.3773	908.3955	437.2680	4.0181	1.5E+05	1	2	2
3310	18.31	320.6344	322.6413	173.1523	2.0069	8.5E+03	2	2	5
3314	18.34	362.1893	364.1953	128.1310	2.0059	3.1E+04	1	1	1
3318	18.36	355.6372	357.6444	243.1579	2.0071	1.1E+04	2	2	2
3328	18.43	452.6916	454.7009	437.2666	2.0093	1.7E+05	2	2	2
3344	18.47	369.0672	371.0729	270.0178	2.0056	1.1E+04	2	2	1
3345	18.48	453.1911	455.2020	438.2655	2.0109	6.7E+03	2	2	2
3346	18.48	924.3568	926.3613	690.2984	2.0046	1.9E+04	1	1	4
3352	18.52	368.0675	370.0742	268.0184	2.0067	1.9E+04	2	2	1
3361	18.57	362.1895	364.1956	128.1312	2.0060	3.2E+04	1	1	1
3364	18.58	381.0938	383.0999	147.0355	2.0060	1.8E+04	1	1	4
3365	18.58	453.1909	455.2018	438.2651	2.0110	1.1E+04	2	2	5
3371	18.60	356.6459	358.6522	245.1751	2.0063	2.0E+04	2	2	6
3378	18.65	324.1049	326.1112	90.0466	2.0063	1.3E+04	1	1	5
3388	18.73	311.0832	313.0898	154.0497	2.0066	7.0E+04	2	2	10
3403	18.83	389.1245	391.1325	310.1324	2.0080	1.5E+04	2	2	1
3405	18.84	751.2035	755.2162	284.0941	4.0127	1.9E+04	1	2	1
3410	18.89	389.1201	391.1257	310.1237	2.0056	7.7E+03	2	2	1
3420	18.94	387.1137	389.1188	153.0554	2.0051	1.1E+04	1	1	1
3425	18.97	409.1245	411.1303	175.0662	2.0058	1.7E+04	1	1	5
3431	19.07	908.3958	910.3975	674.3375	2.0017	1.8E+04	1	1	3
3435	19.11	354.5653	356.5715	241.0140	2.0062	6.2E+03	2	2	5
3442	19.13	690.1395	692.1403	456.0812	2.0007	1.4E+04	1	1	2
3443	19.14	711.1324	715.1505	244.0230	4.0182	8.5E+03	1	2	2
3444	19.15	409.1245	411.1311	175.0662	2.0066	2.7E+04	1	1	5
3447	19.16	347.0887	349.0953	113.0304	2.0066	2.4E+05	1	1	1
3452	19.19	904.3773	908.3951	437.2680	4.0178	1.1E+04	1	2	2
3453	19.19	354.0792	356.0876	120.0209	2.0084	6.4E+03	1	1	10
3454	19.20	736.1931	738.1982	502.1348	2.0051	1.3E+05	1	1	3
3460	19.22	318.0793	320.0863	84.0210	2.0069	1.4E+05	1	1	13
3461	19.22	276.0689	278.0754	42.0105	2.0066	1.3E+05	1	1	4
3464	19.23	736.1940	740.2074	269.0847	4.0134	3.6E+05	1	2	1
3477	19.33	736.1929	740.2061	269.0836	4.0131	1.3E+05	1	2	1
3478	19.33	908.3964	910.3987	674.3380	2.0023	1.5E+04	1	1	3
3479	19.33	904.3767	908.3928	437.2673	4.0161	1.0E+04	1	2	2
3483	19.40	428.6061	430.6128	389.0955	2.0068	7.4E+03	2	2	1

3485	19.42	307.1473	309.1539	73.0889	2.0067	1.5E+05	1	1	8
3488	19.45	359.1018	361.1114	250.0870	2.0096	7.7E+03	2	2	4
3489	19.46	697.3646	699.3695	463.3063	2.0049	1.0E+04	1	1	19
3498	19.55	717.1974	721.2095	250.0881	4.0121	8.4E+03	1	2	4
3501	19.56	421.1579	423.1653	374.1992	2.0074	6.8E+03	2	2	1
3504	19.66	303.0795	305.0859	69.0211	2.0064	2.0E+04	1	1	3
3507	19.67	375.1186	377.1247	282.1205	2.0061	6.5E+03	2	2	13
3508	19.69	697.3643	699.3702	463.3060	2.0059	1.4E+04	1	1	19
3512	19.72	721.2976	723.2978	487.2393	2.0001	3.1E+04	1	1	1
3514	19.75	718.2892	720.2942	484.2309	2.0050	9.2E+03	1	1	1
3517	19.76	699.3185	701.3196	465.2602	2.0011	2.5E+04	1	1	4
3526	19.84	757.3709	759.3729	523.3125	2.0020	7.1E+03	1	1	2
3529	19.86	476.2203	478.2265	242.1620	2.0062	7.8E+03	1	1	4
3534	20.03	363.6531	365.6603	259.1896	2.0072	7.3E+03	2	2	3
3536	20.11	311.0827	313.0893	154.0488	2.0065	8.4E+03	2	2	10
3541	20.18	697.3635	699.3702	463.3052	2.0068	1.6E+04	1	1	19
3550	20.25	421.1791	423.1853	187.1208	2.0062	8.8E+03	1	1	22
3551	20.25	363.6527	365.6601	259.1887	2.0075	9.8E+03	2	2	3
3552	20.26	712.1815	716.1955	245.0722	4.0140	8.5E+03	1	2	6
3554	20.26	600.1055	602.1077	366.0472	2.0022	1.8E+04	1	1	9
3556	20.27	715.1922	717.1939	481.1339	2.0017	1.9E+04	1	1	1
3562	20.28	593.1626	595.1671	359.1043	2.0044	1.5E+04	1	1	1
3566	20.34	699.3715	701.3729	465.3131	2.0015	1.2E+04	1	1	2
3567	20.34	697.3632	699.3704	463.3048	2.0072	1.6E+04	1	1	19
3570	20.35	759.3673	761.3714	525.3090	2.0041	7.3E+03	1	1	5
3571	20.36	368.0994	370.1068	268.0822	2.0074	7.6E+03	2	2	3
3575	20.42	361.1284	363.1328	254.1401	2.0044	8.9E+03	2	2	1
3576	20.44	759.3656	761.3689	525.3073	2.0033	8.3E+03	1	1	9
3580	20.47	590.1352	592.1421	356.0769	2.0069	7.6E+03	1	1	2
3584	20.49	669.3058	671.3085	435.2475	2.0027	3.6E+04	1	1	3
3590	20.58	353.1060	355.1131	238.0954	2.0071	1.0E+04	2	2	19
3592	20.65	697.3641	699.3705	463.3058	2.0064	1.2E+04	1	1	19
3599	20.74	667.2935	669.3055	433.2352	2.0119	1.0E+04	1	1	1
3600	20.74	697.3640	699.3693	463.3057	2.0052	3.7E+04	1	1	19
3605	20.79	333.1635	335.1698	99.1052	2.0063	7.9E+03	1	1	1
3611	20.85	383.1033	385.1118	298.0899	2.0085	9.1E+03	2	2	16
3617	20.90	757.3495	759.3560	523.2912	2.0065	9.1E+03	1	1	9
3620	20.91	383.6025	385.6093	299.0883	2.0068	1.4E+04	2	2	4
3622	20.91	564.2565	566.2602	330.1982	2.0037	3.5E+04	1	1	9
3626	20.93	765.1979	769.2149	298.0885	4.0171	6.9E+03	1	2	7
3627	20.93	382.6019	384.6088	297.0872	2.0069	5.9E+04	2	2	19
3630	20.96	697.3639	699.3707	463.3056	2.0068	1.2E+04	1	1	19
3631	20.96	674.2417	678.2558	207.1323	4.0142	8.4E+03	1	2	1
3635	20.98	454.2048	456.2111	220.1464	2.0064	6.8E+03	1	1	1
3636	20.99	631.2061	633.2072	397.1478	2.0011	3.4E+04	1	1	3
3638	21.01	412.1811	414.1865	356.2455	2.0054	6.0E+03	2	2	1
3639	21.03	980.4081	984.4266	513.2988	4.0185	6.5E+03	1	2	1
3641	21.05	759.3638	761.3704	525.3055	2.0066	1.4E+04	1	1	9

3645	21.08	588.2253	590.2320	354.1670	2.0067	6.5E+03	1	1	1
3647	21.10	741.3870	743.3956	507.3287	2.0086	8.3E+03	1	1	5
3653	21.16	697.3627	699.3706	463.3044	2.0079	2.0E+04	1	1	19
3655	21.22	368.1059	370.1100	134.0476	2.0041	1.2E+04	1	1	1
3657	21.27	697.3649	699.3709	463.3066	2.0059	2.2E+04	1	1	19
3660	21.39	317.5931	319.5997	167.0696	2.0066	1.9E+04	2	2	20
3669	22.02	736.1926	740.2063	269.0833	4.0136	5.2E+04	1	2	1
3677	22.14	711.1329	713.1343	477.0746	2.0014	3.7E+04	1	1	1
3679	22.24	662.0613	664.0658	428.0030	2.0045	1.1E+04	1	1	3
3681	22.29	349.1585	351.1640	115.1002	2.0055	2.1E+04	1	1	21
3684	22.31	360.1140	362.1195	252.1114	2.0055	1.1E+04	2	2	8
3688	22.34	759.3612	761.3696	525.3029	2.0084	1.5E+04	1	1	4
3691	22.34	697.3635	699.3701	463.3052	2.0066	9.1E+03	1	1	19
3694	22.36	699.3717	701.3766	465.3133	2.0049	1.4E+04	1	1	2
3695	22.36	335.1242	337.1308	202.1317	2.0066	6.7E+03	2	2	14
3697	22.38	711.1323	713.1334	477.0740	2.0011	1.7E+04	1	1	1
3699	22.41	518.2568	520.2620	284.1985	2.0052	6.4E+03	1	1	8
3705	22.45	697.3652	699.3715	463.3069	2.0063	1.3E+04	1	1	19
3708	22.47	557.2073	559.2148	323.1489	2.0075	1.5E+04	1	1	8
3711	22.49	450.1361	452.1402	216.0778	2.0041	1.3E+04	1	1	2
3713	22.49	311.0829	313.0895	154.0492	2.0066	8.9E+03	2	2	10
3726	22.53	355.0636	357.0696	242.0107	2.0060	2.2E+04	2	2	7
3727	22.53	654.1871	656.1859	420.1288	1.9988	5.4E+04	1	1	12
3728	22.53	651.1773	653.1816	417.1189	2.0044	6.4E+03	1	1	10
3731	22.53	650.1736	654.1863	183.0642	4.0127	8.4E+03	1	2	15
3732	22.54	699.3704	701.3785	465.3121	2.0081	1.3E+04	1	1	4
3733	22.54	711.1315	713.1317	477.0732	2.0003	6.3E+04	1	1	1
3736	22.56	759.3623	761.3696	525.3040	2.0073	2.1E+04	1	1	4
3739	22.58	354.0638	356.0706	120.0055	2.0068	1.1E+04	1	1	4
3741	22.62	431.1036	433.1096	197.0452	2.0061	9.4E+03	1	1	7
3747	22.68	697.3644	699.3713	463.3061	2.0069	1.1E+04	1	1	19
3748	22.69	757.3489	759.3613	523.2906	2.0124	8.5E+03	1	1	9
3750	22.70	359.0931	361.1006	250.0695	2.0076	7.8E+03	2	2	27
3751	22.70	352.0964	354.1069	118.0381	2.0105	7.0E+03	1	1	5
3756	22.73	699.3714	701.3806	465.3130	2.0093	1.2E+04	1	1	2
3757	22.75	611.2354	613.2375	377.1771	2.0020	1.5E+04	1	1	1
3768	22.78	318.0791	320.0861	84.0207	2.0070	8.8E+04	1	1	13
3769	22.78	348.0900	350.0966	114.0317	2.0066	5.1E+04	1	1	28
3776	22.80	717.1789	721.1923	250.0696	4.0134	4.0E+04	1	2	27
3780	22.81	697.3636	699.3705	463.3053	2.0069	1.9E+04	1	1	19
3781	22.82	311.0828	313.0893	154.0490	2.0065	1.8E+05	2	2	10
3782	22.82	718.1840	720.1854	484.1257	2.0014	1.5E+04	1	1	8
3783	22.82	388.1068	390.1132	154.0485	2.0064	4.5E+04	1	1	10
3787	22.84	759.3620	761.3686	525.3037	2.0066	1.9E+04	1	1	4
3788	22.87	757.3498	759.3623	523.2915	2.0125	7.7E+03	1	1	9
3791	22.91	697.3637	699.3697	463.3054	2.0060	8.8E+03	1	1	19
3792	22.91	761.3695	763.3702	527.3111	2.0008	1.1E+04	1	1	3
3801	23.01	761.3679	763.3717	527.3096	2.0038	8.0E+03	1	1	3

3804	23.03	373.1204	375.1284	278.1242	2.0080	1.0E+04	2	2	2
3805	23.03	699.3705	701.3795	465.3122	2.0090	1.4E+04	1	1	4
3808	23.04	431.1036	433.1088	197.0453	2.0052	6.6E+03	1	1	7
3810	23.05	392.1642	394.1708	158.1059	2.0066	1.3E+04	1	1	6
3818	23.07	697.3621	699.3697	463.3038	2.0076	9.3E+03	1	1	2
3824	23.08	725.4323	727.4347	491.3740	2.0023	1.1E+04	1	1	2
3825	23.08	747.4143	749.4155	513.3560	2.0012	6.5E+04	1	1	1
3835	23.11	640.2084	642.2102	406.1501	2.0017	1.3E+04	1	1	7
3837	23.11	639.1511	641.1533	405.0928	2.0022	1.2E+04	1	1	1
3844	23.12	603.1581	605.1580	369.0998	1.9999	1.9E+04	1	1	2
3846	23.13	697.3660	699.3715	463.3077	2.0055	1.0E+04	1	1	17
3847	23.13	484.2146	486.2213	250.1563	2.0067	6.2E+03	1	1	1
3852	23.13	301.0761	303.0821	134.0355	2.0060	1.3E+04	2	2	1
3858	23.15	761.3709	763.3753	527.3125	2.0044	7.4E+03	1	1	2
3861	23.17	757.3530	759.3592	523.2947	2.0062	6.8E+03	1	1	4
3868	23.19	699.3727	701.3813	465.3144	2.0086	1.1E+04	1	1	2
3871	23.19	359.1063	361.1123	250.0960	2.0060	1.3E+04	2	2	15
3872	23.20	328.1035	330.1122	188.0903	2.0087	1.4E+04	2	2	3
3879	23.22	699.3174	701.3196	465.2590	2.0022	1.2E+04	1	1	4
3884	23.24	697.3662	699.3719	463.3079	2.0057	1.2E+04	1	1	17
3886	23.25	753.3552	755.3573	519.2969	2.0021	9.0E+03	1	1	22
3888	23.25	721.2990	723.3005	487.2406	2.0015	1.4E+04	1	1	1
3889	23.26	437.1564	439.1605	203.0981	2.0041	1.2E+04	1	1	7
3891	23.26	759.3552	761.3601	525.2969	2.0050	6.9E+03	1	1	5
3893	23.27	759.3630	761.3705	525.3047	2.0075	2.1E+04	1	1	9
3894	23.28	652.1965	654.1997	418.1382	2.0032	8.5E+03	1	1	4
3900	23.32	397.1572	399.1634	163.0989	2.0062	9.8E+03	1	1	16
3903	23.34	761.3694	763.3727	527.3111	2.0033	9.4E+03	1	1	3
3904	23.35	699.3728	701.3817	465.3145	2.0089	1.0E+04	1	1	2
3906	23.35	466.2153	468.2205	232.1570	2.0051	1.3E+04	1	1	1
3911	23.38	759.3559	761.3557	525.2976	1.9998	6.8E+03	1	1	5
3917	23.43	759.3623	761.3707	525.3040	2.0084	1.1E+04	1	1	4
3918	23.43	761.3670	763.3680	527.3087	2.0010	1.1E+04	1	1	3
3925	23.47	360.1138	362.1198	252.1110	2.0060	1.1E+04	2	2	8
3926	23.47	697.3653	699.3728	463.3070	2.0076	8.6E+03	1	1	17
3929	23.48	757.3497	759.3606	523.2914	2.0109	7.5E+03	1	1	9
3931	23.51	761.3713	763.3731	527.3130	2.0018	1.1E+04	1	1	2
3934	23.52	313.0765	315.0836	158.0364	2.0071	1.1E+04	2	2	1
3939	23.53	759.3556	761.3599	525.2972	2.0044	6.6E+03	1	1	5
3941	23.55	387.0892	389.0953	153.0309	2.0061	2.3E+04	1	1	2
3946	23.58	757.3482	759.3611	523.2899	2.0130	6.2E+03	1	1	9
3949	23.62	699.3711	701.3780	465.3128	2.0069	1.3E+04	1	1	2
3952	23.66	699.3709	701.3823	465.3125	2.0114	1.1E+04	1	1	4
3954	23.68	306.0614	308.0680	144.0061	2.0067	1.2E+04	2	2	5
3955	23.69	374.1288	376.1366	140.0705	2.0078	9.8E+03	1	1	7
3957	23.69	458.1982	460.2058	224.1399	2.0076	9.5E+03	1	1	2
3958	23.71	761.3707	763.3720	527.3124	2.0013	1.1E+04	1	1	2
3960	23.72	697.3617	699.3709	463.3034	2.0092	1.0E+04	1	1	2

3961	23.72	759.3626	761.3696	525.3043	2.0070	1.6E+04	1	1	9
3962	23.73	428.0650	430.0698	194.0067	2.0048	9.4E+03	1	1	1
3963	23.74	374.1295	376.1344	280.1423	2.0049	1.5E+04	2	2	11
3965	23.78	699.3711	701.3808	465.3128	2.0096	9.0E+03	1	1	2
3969	23.85	699.3720	701.3845	465.3137	2.0125	1.1E+04	1	1	2
3974	23.86	432.1215	434.1273	198.0632	2.0058	2.6E+04	1	1	20
3975	23.86	507.1548	509.1581	273.0965	2.0033	1.3E+04	1	1	40
3979	23.89	639.3329	641.3371	405.2746	2.0042	1.7E+04	1	1	6
3981	23.91	697.3646	699.3715	463.3063	2.0069	9.9E+03	1	1	19
3985	23.95	759.3635	761.3702	525.3052	2.0067	1.1E+04	1	1	9
3986	23.96	363.1732	365.1799	129.1149	2.0067	3.9E+04	1	1	10
3987	23.97	988.4361	990.4440	754.3778	2.0079	1.5E+04	1	1	1
3990	23.99	567.2093	571.2228	100.0999	4.0136	2.0E+04	1	2	2
3991	23.99	431.1032	433.1083	197.0449	2.0051	4.5E+04	1	1	7
3992	23.99	349.1637	351.1715	230.2108	2.0078	1.7E+04	2	2	2
3993	24.00	488.6838	490.6934	509.2511	2.0096	7.2E+03	2	2	2
3994	24.01	276.0926	278.1015	84.0685	2.0089	7.0E+03	2	2	2
4000	24.02	695.3478	697.3597	461.2894	2.0119	1.1E+04	1	1	19
4002	24.04	348.0901	350.0976	114.0317	2.0075	9.1E+03	1	1	28
4009	24.08	431.1035	433.1098	197.0452	2.0062	4.2E+04	1	1	7
4010	24.08	717.1796	721.1924	250.0703	4.0127	7.0E+03	1	2	13
4014	24.11	699.3723	701.3817	465.3140	2.0093	8.8E+03	1	1	2
4016	24.12	474.1942	476.2041	480.2718	2.0099	1.5E+04	2	2	23
4021	24.13	625.2621	629.2762	158.1528	4.0141	1.2E+04	1	2	3
4023	24.14	695.3491	697.3584	461.2908	2.0093	1.6E+04	1	1	19
4026	24.16	332.5588	334.5643	197.0009	2.0055	2.6E+04	2	2	1
4027	24.16	944.4095	946.4196	710.3512	2.0101	1.2E+04	1	1	2
4028	24.16	759.3610	761.3708	525.3027	2.0097	8.0E+03	1	1	4
4030	24.17	363.1732	365.1795	129.1148	2.0063	1.6E+04	1	1	10
4031	24.17	331.5593	333.5659	195.0020	2.0066	2.1E+04	2	2	2
4039	24.23	729.3562	731.3610	495.2979	2.0047	6.4E+03	1	1	13
4041	24.25	567.2089	571.2223	100.0996	4.0133	9.9E+03	1	2	2
4042	24.25	653.1664	655.1682	419.1081	2.0017	1.3E+04	1	1	5
4044	24.26	759.3624	761.3708	525.3040	2.0084	7.8E+03	1	1	4
4046	24.28	697.3571	699.3720	463.2988	2.0149	7.3E+03	1	1	4
4047	24.28	329.0792	331.0856	190.0417	2.0064	1.4E+04	2	2	2
4051	24.30	388.1114	390.1157	154.0531	2.0043	8.4E+03	1	1	1
4052	24.30	388.1054	390.1134	154.0471	2.0080	6.3E+03	1	1	2
4061	24.34	620.1550	624.1634	153.0457	4.0083	9.8E+03	1	2	4
4062	24.35	699.3719	701.3820	465.3136	2.0101	6.8E+03	1	1	2
4064	24.37	900.3848	902.3917	666.3265	2.0069	6.5E+03	1	1	1
4065	24.40	441.1809	443.1874	207.1226	2.0065	1.9E+04	1	1	3
4069	24.46	556.1293	558.1344	322.0710	2.0051	4.4E+04	1	1	2
4073	24.47	695.3500	697.3591	461.2917	2.0091	7.9E+03	1	1	16
4076	24.49	759.3632	761.3711	525.3048	2.0079	1.2E+04	1	1	9
4080	24.52	697.3617	699.3726	463.3033	2.0110	7.7E+03	1	1	2
4084	24.54	428.0645	430.0702	194.0062	2.0056	7.0E+04	1	1	1
4085	24.55	450.1583	452.1631	216.1000	2.0048	1.2E+04	1	1	10

4087	24.57	474.1940	476.2035	480.2714	2.0094	1.1E+04	2	2	23
4091	24.61	431.1030	433.1091	197.0447	2.0062	3.2E+04	1	1	7
4092	24.61	759.3609	761.3714	525.3026	2.0105	6.2E+03	1	1	4
4093	24.62	695.3488	697.3610	461.2905	2.0122	9.1E+03	1	1	19
4098	24.70	357.1376	359.1435	123.0793	2.0059	1.6E+04	1	1	3
4099	24.70	590.1889	594.2022	123.0796	4.0133	9.4E+03	1	2	3
4101	24.70	759.3621	761.3703	525.3038	2.0083	9.4E+03	1	1	4
4104	24.71	530.6942	532.7033	593.2719	2.0091	7.7E+03	2	2	2
4110	24.72	485.1242	487.1329	251.0659	2.0087	1.1E+04	1	1	15
4111	24.73	356.5946	358.6011	245.0725	2.0066	3.0E+04	2	2	6
4112	24.73	357.5935	359.6003	247.0703	2.0068	8.1E+03	2	2	12
4116	24.75	810.3277	814.3363	343.2184	4.0086	7.2E+03	1	2	1
4121	24.76	451.1723	453.1773	217.1140	2.0050	1.2E+04	1	1	4
4122	24.77	601.1490	603.1590	367.0906	2.0100	8.8E+03	1	1	4
4127	24.79	984.3650	988.3839	517.2557	4.0189	1.6E+04	1	2	3
4130	24.79	473.1894	475.1999	239.1310	2.0105	2.4E+04	1	1	3
4133	24.80	472.1899	474.1960	238.1315	2.0062	6.0E+04	1	1	3
4136	24.81	355.1636	357.1700	242.2106	2.0063	1.1E+05	2	2	1
4148	24.83	349.1641	351.1701	230.2116	2.0060	1.1E+04	2	2	2
4160	24.91	697.3621	699.3704	463.3038	2.0083	9.9E+03	1	1	2
4171	25.00	759.3629	761.3695	525.3046	2.0065	8.8E+03	1	1	9
4175	25.02	416.6560	418.6623	365.1953	2.0063	6.9E+03	2	2	2
4176	25.03	677.1801	679.1877	443.1217	2.0077	8.1E+03	1	1	2
4177	25.04	680.1909	682.1922	446.1326	2.0013	1.5E+04	1	1	1
4182	25.07	611.2453	613.2486	377.1869	2.0034	9.6E+03	1	1	5
4190	25.12	579.1878	581.1903	345.1295	2.0025	1.3E+04	1	1	2
4193	25.12	300.0804	302.0840	132.0442	2.0036	7.7E+03	2	2	4
4196	25.13	557.2070	559.2110	323.1487	2.0040	1.5E+04	1	1	8
4197	25.13	570.2151	572.2189	336.1568	2.0038	6.2E+03	1	1	1
4200	25.13	452.1877	454.1944	218.1294	2.0066	8.4E+03	1	1	1
4202	25.13	611.2476	615.2587	144.1383	4.0111	1.1E+04	1	2	3
4203	25.14	644.2081	646.2106	410.1497	2.0025	9.6E+03	1	1	3
4204	25.14	323.1064	325.1127	178.0961	2.0064	3.4E+04	2	2	8
4207	25.15	759.3627	761.3720	525.3043	2.0094	8.3E+03	1	1	9
4208	25.15	277.1004	279.1086	86.0842	2.0082	3.0E+04	2	2	1
4209	25.16	647.2174	649.2189	413.1590	2.0015	2.7E+04	1	1	2
4212	25.18	424.1618	426.1701	380.2070	2.0083	9.5E+03	2	2	1
4215	25.21	431.1039	433.1099	197.0455	2.0060	1.0E+04	1	1	7
4218	25.22	664.2577	666.2636	430.1994	2.0059	1.4E+04	1	1	6
4223	25.27	452.1883	454.1942	218.1299	2.0060	2.2E+04	1	1	1
4225	25.29	530.1832	532.1921	296.1249	2.0089	9.0E+03	1	1	7
4227	25.30	759.3628	761.3706	525.3044	2.0078	8.0E+03	1	1	9
4229	25.31	287.5769	289.5831	107.0371	2.0063	8.2E+03	2	2	5
4230	25.32	467.1902	469.1993	233.1319	2.0091	6.8E+03	1	1	2
4231	25.33	431.1023	433.1097	197.0440	2.0074	9.2E+03	1	1	5
4233	25.36	466.1914	468.1957	464.2661	2.0043	9.3E+03	2	2	1
4242	25.37	359.1014	361.1092	250.0861	2.0078	6.9E+03	2	2	1
4245	25.38	388.1072	390.1137	154.0488	2.0065	6.4E+04	1	1	10

4250	25.39	318.0793	320.0866	84.0209	2.0074	1.1E+04	1	1	13
4254	25.39	473.6964	475.7060	479.2761	2.0096	2.2E+04	2	2	2
4255	25.39	969.3722	973.3923	502.2628	4.0201	8.1E+03	1	2	1
4257	25.39	605.1571	607.1610	371.0987	2.0039	2.0E+04	1	1	6
4280	25.41	753.2008	757.2142	286.0914	4.0134	7.4E+03	1	2	7
4294	25.43	464.2694	466.2757	230.2111	2.0063	8.3E+03	1	1	2
4295	25.44	933.3748	935.3870	699.3165	2.0122	6.2E+03	1	1	5
4299	25.46	349.1635	351.1700	230.2103	2.0065	7.0E+04	2	2	2
4302	25.47	466.1897	468.1962	232.1314	2.0065	3.7E+04	1	1	12
4303	25.47	467.1899	469.2002	466.2631	2.0104	1.8E+04	2	2	1
4304	25.48	599.1520	601.1532	365.0937	2.0012	2.5E+04	1	1	1
4305	25.48	661.2012	665.2152	194.0919	4.0139	8.8E+03	1	2	2
4313	25.51	946.3840	950.4040	479.2747	4.0200	6.5E+03	1	2	1
4314	25.51	431.1037	433.1099	197.0454	2.0062	1.2E+04	1	1	7
4315	25.52	759.3638	761.3701	525.3055	2.0064	8.9E+03	1	1	9
4316	25.53	487.1798	489.1857	253.1215	2.0059	4.2E+04	1	1	2
4317	25.54	442.2039	444.2107	208.1456	2.0068	9.8E+03	1	1	2
4322	25.58	352.6095	354.6162	237.1024	2.0067	7.0E+03	2	2	3
4326	25.59	460.2143	462.2207	226.1560	2.0064	6.7E+03	1	1	3
4327	25.59	697.3639	699.3688	463.3056	2.0049	2.3E+04	1	1	19
4330	25.62	663.2020	665.2100	429.1437	2.0080	1.3E+04	1	1	2
4334	25.63	530.1834	532.1901	296.1250	2.0068	2.8E+04	1	1	7
4335	25.64	664.2570	666.2625	430.1986	2.0055	1.6E+05	1	1	6
4336	25.65	720.2525	724.2645	253.1431	4.0120	8.4E+03	1	2	8
4338	25.66	460.1729	462.1827	452.2292	2.0098	1.0E+04	2	2	4
4340	25.68	717.4392	719.4406	483.3809	2.0014	1.4E+04	1	1	1
4343	25.73	967.4203	969.4304	733.3620	2.0101	2.0E+04	1	1	1
4347	25.80	431.1017	433.1090	197.0433	2.0074	1.0E+04	1	1	5
4348	25.80	697.3627	699.3707	463.3044	2.0079	2.9E+04	1	1	19
4351	25.81	620.2433	622.2500	386.1850	2.0067	1.2E+04	1	1	7
4354	25.82	759.3630	761.3711	525.3046	2.0081	6.9E+03	1	1	9
4358	25.86	664.2567	666.2623	430.1983	2.0057	1.4E+05	1	1	8
4363	25.88	595.1757	597.1820	361.1174	2.0062	6.5E+03	1	1	2
4364	25.89	652.2255	656.2406	185.1161	4.0152	7.2E+03	1	2	3
4365	25.90	345.5573	347.5633	222.9980	2.0060	1.3E+04	2	2	3
4366	25.90	431.1033	433.1095	197.0450	2.0062	8.7E+03	1	1	7
4367	25.91	530.1832	532.1890	296.1248	2.0058	8.6E+03	1	1	7
4373	25.96	358.0970	360.1040	124.0387	2.0070	8.2E+03	1	1	2
4377	25.98	316.0525	318.0591	163.9885	2.0066	1.3E+04	2	2	1
4385	26.02	667.1890	671.2023	200.0796	4.0133	1.2E+04	1	2	11
4389	26.02	518.2193	520.2266	284.1610	2.0074	8.7E+03	1	1	3
4393	26.03	530.1831	532.1896	296.1247	2.0066	9.2E+03	1	1	18
4395	26.05	431.1036	433.1102	197.0453	2.0066	8.5E+03	1	1	7
4397	26.05	664.2569	666.2627	430.1986	2.0057	5.1E+04	1	1	6
4399	26.05	759.3647	761.3743	525.3064	2.0096	8.1E+03	1	1	9
4400	26.07	613.1478	615.1498	379.0895	2.0020	1.7E+04	1	1	5
4402	26.09	668.2041	670.2052	434.1458	2.0011	1.4E+04	1	1	14
4403	26.09	635.1303	637.1332	401.0720	2.0028	1.4E+04	1	1	5

4405	26.09	671.2036	673.2057	437.1452	2.0021	9.9E+03	1	1	18
4411	26.11	697.3636	699.3708	463.3053	2.0072	9.1E+03	1	1	19
4412	26.11	671.2128	673.2165	437.1545	2.0037	1.2E+04	1	1	7
4414	26.12	706.3187	708.3224	472.2604	2.0037	1.0E+04	1	1	19
4415	26.12	678.3241	680.3298	444.2658	2.0057	9.0E+03	1	1	7
4422	26.14	380.1285	382.1348	292.1404	2.0063	1.0E+04	2	2	3
4423	26.14	803.3331	805.3398	569.2748	2.0067	1.3E+04	1	1	7
4426	26.15	431.1031	433.1099	197.0447	2.0068	9.9E+03	1	1	7
4430	26.21	610.2214	612.2291	376.1630	2.0077	6.9E+03	1	1	3
4445	26.31	664.2561	666.2629	430.1978	2.0068	2.3E+04	1	1	8
4448	26.33	648.2616	650.2680	414.2033	2.0064	1.7E+04	1	1	2
4450	26.34	431.1034	433.1092	197.0450	2.0058	1.0E+04	1	1	7
4451	26.37	697.3626	699.3699	463.3043	2.0073	1.0E+04	1	1	19
4461	26.43	668.3050	670.3078	434.2467	2.0028	9.1E+03	1	1	1
4464	26.45	309.0983	311.1040	150.0800	2.0057	6.9E+03	2	2	8
4466	26.50	393.1243	395.1308	318.1319	2.0065	7.2E+03	2	2	5
4468	26.51	689.1873	691.1884	455.1290	2.0011	1.6E+04	1	1	6
4470	26.51	779.3372	781.3420	545.2789	2.0048	4.4E+04	1	1	3
4473	26.55	723.3451	725.3509	489.2867	2.0058	1.8E+04	1	1	12
4474	26.55	431.1039	433.1106	197.0456	2.0067	1.2E+04	1	1	7
4480	26.55	684.2316	686.2325	450.1733	2.0009	1.2E+04	1	1	1
4485	26.58	697.3630	699.3720	463.3046	2.0091	8.7E+03	1	1	19
4490	26.58	725.3519	727.3549	491.2936	2.0030	9.9E+03	1	1	2
4501	26.65	424.1625	426.1720	380.2083	2.0095	3.1E+05	2	2	3
4508	26.68	283.1113	285.1176	49.0529	2.0064	5.6E+05	1	1	3
4511	26.70	423.1620	425.1686	189.1037	2.0066	8.9E+04	1	1	2
4514	26.72	639.1397	641.1404	405.0814	2.0006	1.1E+04	1	1	2
4516	26.72	662.2420	664.2496	428.1836	2.0076	9.0E+03	1	1	1
4517	26.73	639.1401	643.1529	172.0307	4.0128	1.1E+04	1	2	1
4520	26.74	421.1576	423.1609	187.0992	2.0033	5.1E+04	1	1	2
4522	26.75	620.2433	622.2499	386.1850	2.0066	7.6E+04	1	1	7
4523	26.76	689.1887	691.1908	455.1303	2.0021	1.3E+04	1	1	4
4528	26.79	589.1787	591.1801	355.1204	2.0013	1.1E+04	1	1	1
4529	26.79	567.1254	571.1394	100.0161	4.0140	1.0E+04	1	2	16
4530	26.80	431.1031	433.1095	197.0447	2.0064	1.8E+04	1	1	7
4531	26.81	611.1603	613.1616	377.1020	2.0013	1.2E+04	1	1	2
4532	26.81	500.2463	502.2524	266.1879	2.0062	8.2E+03	1	1	10
4539	26.85	697.3623	699.3704	463.3040	2.0080	1.1E+04	1	1	19
4550	26.88	738.2906	740.2960	504.2322	2.0054	1.5E+04	1	1	1
4551	26.88	835.3039	837.3094	601.2456	2.0055	1.9E+04	1	1	1
4555	26.89	815.3290	817.3291	581.2707	2.0002	5.2E+05	1	1	4
4557	26.89	712.3017	714.3036	478.2434	2.0019	1.1E+04	1	1	3
4561	26.90	847.3217	851.3387	380.2124	4.0169	6.6E+03	1	2	1
4563	26.91	837.3107	839.3114	603.2524	2.0007	2.5E+04	1	1	1
4570	26.91	751.2049	755.2176	284.0956	4.0127	7.8E+03	1	2	1
4571	26.92	706.3142	708.3170	472.2559	2.0028	8.6E+03	1	1	7
4572	26.93	363.2098	365.2166	129.1515	2.0068	2.7E+05	1	1	1
4574	26.93	724.3291	726.3350	490.2708	2.0058	7.5E+03	1	1	3

4576	26.95	652.2717	654.2805	418.2134	2.0088	9.0E+03	1	1	6
4580	26.97	514.1886	516.1955	280.1302	2.0069	7.0E+03	1	1	1
4581	26.97	986.2535	990.2725	519.1441	4.0190	7.1E+03	1	2	1
4582	26.98	664.2563	666.2626	430.1980	2.0063	3.8E+04	1	1	8
4584	26.99	712.1821	716.1958	245.0728	4.0137	1.0E+04	1	2	6
4585	26.99	735.3404	737.3493	501.2821	2.0089	1.5E+04	1	1	5
4586	26.99	813.3229	815.3272	579.2646	2.0043	4.0E+05	1	1	5
4587	26.99	711.3309	713.3421	477.2726	2.0112	1.0E+04	1	1	5
4590	27.00	703.2038	705.2051	469.1455	2.0013	1.8E+04	1	1	4
4591	27.00	946.2310	950.2500	479.1216	4.0190	8.3E+03	1	2	2
4595	27.02	452.1757	454.1847	436.2348	2.0090	6.2E+03	2	2	2
4598	27.04	711.1777	713.1801	477.1194	2.0024	1.9E+04	1	1	2
4607	27.06	485.1247	487.1318	251.0663	2.0071	1.4E+04	1	1	14
4613	27.07	485.6238	487.6334	503.1310	2.0096	2.7E+04	2	2	13
4614	27.07	611.2826	613.2884	377.2243	2.0058	1.3E+04	1	1	1
4616	27.08	572.1242	574.1281	338.0658	2.0039	4.6E+04	1	1	1
4623	27.10	486.1249	488.1348	252.0666	2.0100	7.9E+03	1	1	1
4628	27.14	416.1496	418.1535	182.0913	2.0038	7.5E+03	1	1	4
4629	27.14	353.1065	355.1144	238.0963	2.0079	1.0E+04	2	2	22
4631	27.16	703.2052	705.2043	469.1469	1.9990	3.0E+04	1	1	1
4632	27.16	828.5207	830.5227	594.4624	2.0020	6.6E+03	1	1	21
4635	27.16	275.5767	277.5833	83.0368	2.0065	8.0E+03	2	2	2
4638	27.18	352.1084	354.1146	236.1001	2.0062	1.4E+04	2	2	13
4640	27.19	697.3667	699.3697	463.3084	2.0030	1.3E+04	1	1	17
4641	27.19	446.1985	448.2049	212.1401	2.0065	1.6E+04	1	1	1
4642	27.19	601.1480	603.1494	367.0896	2.0014	9.9E+03	1	1	4
4651	27.23	807.4359	809.4372	573.3776	2.0012	1.3E+05	1	1	7
4654	27.24	470.1973	472.2044	236.1390	2.0070	1.8E+04	1	1	2
4666	27.28	431.1037	433.1088	197.0453	2.0051	1.9E+04	1	1	7
4667	27.28	757.3220	759.3251	523.2637	2.0031	2.8E+04	1	1	3
4668	27.30	567.1615	569.1582	333.1032	1.9966	1.3E+04	1	1	2
4669	27.30	891.2021	893.2011	657.1438	1.9990	1.2E+04	1	1	1
4672	27.30	755.3170	757.3226	521.2586	2.0057	2.7E+04	1	1	2
4674	27.31	697.3647	699.3731	463.3064	2.0084	1.0E+04	1	1	19
4677	27.34	523.1172	525.1228	289.0588	2.0057	6.3E+03	1	1	2
4679	27.34	575.2925	577.2919	341.2341	1.9994	5.0E+04	1	1	4
4680	27.35	784.4962	786.4971	550.4379	2.0009	6.6E+03	1	1	6
4681	27.35	432.1624	434.1656	198.1040	2.0033	8.3E+03	1	1	1
4684	27.36	614.2815	616.2838	380.2232	2.0023	2.4E+04	1	1	1
4693	27.41	809.3467	811.3487	575.2884	2.0020	1.0E+04	1	1	4
4700	27.43	611.1179	615.1307	144.0086	4.0128	8.7E+04	1	2	2
4704	27.45	717.2189	719.2177	483.1606	1.9988	1.8E+04	1	1	9
4706	27.47	611.2821	613.2886	377.2238	2.0065	1.7E+04	1	1	1
4710	27.48	875.2524	877.2557	641.1941	2.0033	1.1E+04	1	1	1
4713	27.50	438.1289	440.1422	408.1411	2.0134	1.7E+04	2	2	2
4714	27.51	263.0850	265.0913	29.0267	2.0063	1.2E+04	1	1	1
4718	27.51	436.1206	438.1305	404.1246	2.0099	2.6E+04	2	2	2
4721	27.52	697.3631	699.3706	463.3048	2.0075	1.4E+04	1	1	19

4724	27.53	431.1031	433.1103	197.0447	2.0072	1.5E+04	1	1	7
4730	27.54	514.1883	516.1953	280.1300	2.0069	1.6E+04	1	1	1
4731	27.55	695.3290	699.3451	228.2196	4.0161	2.8E+04	1	2	1
4732	27.56	665.2884	667.2946	431.2301	2.0062	2.9E+04	1	1	3
4733	27.57	756.3025	758.3070	522.2441	2.0046	1.3E+04	1	1	10
4734	27.57	562.7084	564.7199	657.3002	2.0115	8.3E+03	2	2	1
4747	27.60	462.3408	464.3539	228.2825	2.0130	9.8E+03	1	1	1
4755	27.61	485.1248	487.1315	251.0664	2.0067	2.3E+05	1	1	14
4759	27.61	318.0795	320.0860	84.0212	2.0065	3.8E+04	1	1	13
4761	27.61	736.1934	740.2065	269.0840	4.0131	5.4E+04	1	2	1
4762	27.62	291.0739	293.0806	114.0312	2.0067	1.4E+04	2	2	28
4763	27.62	752.8136	754.8231	1037.5105	2.0095	6.7E+03	2	2	3
4764	27.63	300.0801	302.0866	132.0435	2.0065	1.1E+06	2	2	4
4769	27.65	485.6250	487.6342	503.1333	2.0092	3.0E+05	2	2	13
4770	27.65	737.1915	739.2042	503.1332	2.0127	4.3E+04	1	1	13
4772	27.65	706.3162	708.3214	472.2579	2.0052	9.2E+03	1	1	26
4776	27.69	696.4398	698.4441	462.3814	2.0043	9.1E+03	1	1	2
4777	27.69	717.2212	719.2211	483.1629	1.9999	7.8E+03	1	1	2
4780	27.70	740.2224	744.2392	273.1130	4.0168	1.2E+04	1	2	1
4788	27.72	841.3164	843.3241	607.2581	2.0077	6.4E+03	1	1	14
4791	27.73	865.3060	867.3055	631.2477	1.9995	9.2E+03	1	1	1
4793	27.74	810.2412	812.2477	576.1829	2.0065	7.0E+03	1	1	3
4796	27.74	569.2692	571.2676	335.2109	1.9983	7.2E+04	1	1	2
4802	27.76	736.1931	738.1948	502.1348	2.0017	1.9E+04	1	1	3
4807	27.76	668.2277	670.2394	434.1694	2.0117	9.2E+03	1	1	1
4810	27.77	576.2221	578.2201	342.1638	1.9980	1.1E+04	1	1	1
4811	27.77	567.1589	569.1600	333.1006	2.0011	1.2E+04	1	1	8
4815	27.78	670.2414	672.2513	436.1831	2.0099	8.5E+03	1	1	1
4816	27.78	395.6332	397.6426	323.1497	2.0094	2.2E+04	2	2	8
4818	27.79	843.3248	845.3248	609.2665	1.9999	9.1E+04	1	1	1
4819	27.79	552.2067	554.2081	318.1484	2.0013	8.9E+03	1	1	1
4823	27.79	581.2705	583.2723	347.2122	2.0018	1.2E+04	1	1	5
4828	27.80	289.0761	291.0816	110.0355	2.0055	6.6E+03	2	2	1
4834	27.82	574.2183	576.2186	340.1600	2.0002	1.4E+04	1	1	2
4836	27.82	441.1447	443.1474	207.0863	2.0027	1.9E+04	1	1	1
4838	27.83	648.2603	650.2664	414.2020	2.0061	5.3E+04	1	1	4
4839	27.84	570.2194	572.2313	672.3222	2.0118	8.9E+03	2	2	6
4840	27.84	581.1763	583.1736	347.1180	1.9973	1.5E+04	1	1	1
4841	27.84	369.1024	371.1061	270.0881	2.0038	9.9E+03	2	2	9
4842	27.84	556.2738	558.2777	322.2155	2.0038	2.8E+04	1	1	52
4845	27.85	663.3923	665.3968	429.3340	2.0044	2.8E+04	1	1	1
4849	27.87	549.1733	551.1726	315.1150	1.9993	2.3E+04	1	1	1
4853	27.88	682.3619	684.3648	448.3036	2.0029	3.4E+04	1	1	4
4857	27.90	456.2200	458.2261	222.1617	2.0061	8.4E+03	1	1	2
4859	27.95	735.3403	737.3465	501.2820	2.0062	2.8E+04	1	1	5
4863	28.03	620.2323	622.2388	386.1740	2.0065	1.2E+05	1	1	2
4866	28.03	683.3552	685.3552	449.2969	2.0000	2.5E+04	1	1	1
4884	28.17	301.0786	303.0842	134.0406	2.0056	3.2E+04	2	2	3

4887	28.18	736.1934	740.2084	269.0841	4.0150	3.7E+04	1	2	1
4888	28.20	739.2032	741.2040	505.1448	2.0009	1.6E+04	1	1	4
4893	28.23	576.2205	578.2228	342.1621	2.0023	7.2E+03	1	1	2
4897	28.25	476.6190	478.6291	485.1213	2.0101	6.7E+03	2	2	3
4898	28.25	737.1913	739.2038	503.1329	2.0126	8.1E+03	1	1	13
4899	28.26	458.1980	460.2042	224.1397	2.0062	3.4E+04	1	1	2
4901	28.26	648.2578	650.2664	414.1995	2.0086	3.3E+04	1	1	5
4904	28.27	697.3632	699.3713	463.3049	2.0081	8.2E+03	1	1	19
4906	28.27	446.1988	448.2043	212.1405	2.0055	8.3E+03	1	1	8
4907	28.27	668.2284	670.2367	434.1700	2.0083	2.6E+04	1	1	1
4914	28.29	670.2358	672.2458	436.1775	2.0099	1.2E+04	1	1	3
4915	28.29	595.1920	597.1933	361.1337	2.0013	8.0E+03	1	1	2
4921	28.52	617.2095	619.2202	383.1512	2.0107	4.3E+04	1	1	2
4942	28.68	457.1538	459.1606	223.0955	2.0068	5.7E+04	1	1	19
4953	28.75	583.2857	585.2845	349.2274	1.9988	2.0E+04	1	1	1
4954	28.76	528.2440	530.2455	294.1857	2.0015	1.3E+04	1	1	1
4962	28.83	697.3615	699.3738	463.3031	2.0123	6.7E+03	1	1	2
4975	28.90	735.3402	737.3480	501.2818	2.0078	8.3E+03	1	1	5
4978	28.91	738.3506	740.3544	504.2923	2.0038	1.5E+04	1	1	3
4980	28.93	583.2856	585.2844	349.2273	1.9988	3.7E+04	1	1	1
4984	28.94	563.1890	565.1905	329.1307	2.0015	1.1E+04	1	1	1
4988	28.95	856.2100	860.2311	389.1007	4.0210	7.5E+03	1	2	4
4991	28.96	723.3794	725.3865	489.3210	2.0071	7.4E+03	1	1	7
4995	28.97	689.3487	691.3503	455.2904	2.0016	1.6E+04	1	1	7
4997	28.98	621.1579	625.1711	154.0486	4.0132	1.2E+04	1	2	10
4999	28.98	757.3261	759.3325	523.2678	2.0064	9.9E+03	1	1	6
5000	28.99	444.2192	446.2253	210.1609	2.0061	1.3E+05	1	1	1
5001	28.99	737.3474	739.3512	503.2891	2.0038	2.0E+04	1	1	4
5005	29.01	561.1304	563.1317	327.0721	2.0013	1.1E+04	1	1	5
5009	29.02	456.2192	458.2256	222.1609	2.0063	1.7E+05	1	1	2
5012	29.03	659.2478	661.2531	425.1895	2.0053	9.0E+03	1	1	10
5014	29.04	757.4131	759.4174	523.3548	2.0044	1.3E+04	1	1	2
5015	29.04	686.2869	688.2881	452.2286	2.0012	2.5E+04	1	1	2
5018	29.06	684.2801	686.2851	450.2218	2.0050	4.2E+04	1	1	1
5023	29.07	569.2201	571.2253	335.1617	2.0052	1.9E+04	1	1	2
5025	29.08	519.1376	521.1356	285.0793	1.9980	1.4E+04	1	1	6
5041	29.12	405.0630	407.0659	171.0047	2.0029	6.5E+04	1	1	1
5045	29.13	379.1470	381.1516	145.0887	2.0046	9.3E+04	1	1	5
5047	29.13	453.1909	455.2014	438.2651	2.0106	6.2E+03	2	2	5
5049	29.13	686.2860	688.2882	452.2276	2.0022	1.9E+04	1	1	3
5055	29.15	486.2299	488.2362	252.1716	2.0063	1.2E+04	1	1	2
5057	29.15	319.0648	321.0705	170.0130	2.0057	4.4E+05	2	2	2
5065	29.18	542.2599	544.2607	308.2015	2.0009	2.7E+04	1	1	1
5067	29.19	723.3793	725.3864	489.3210	2.0071	6.5E+03	1	1	7
5069	29.20	838.3399	842.3527	371.2306	4.0128	7.5E+03	1	2	3
5075	29.23	700.3463	702.3549	466.2880	2.0086	1.3E+04	1	1	2
5077	29.24	768.3826	770.3955	534.3242	2.0129	6.5E+03	1	1	1
5082	29.25	556.2756	558.2753	322.2173	1.9997	1.0E+05	1	1	1

5085	29.25	789.2989	791.3071	555.2406	2.0082	7.5E+03	1	1	1
5087	29.27	643.1344	645.1361	409.0761	2.0017	2.3E+04	1	1	4
5092	29.30	637.1225	641.1354	170.0132	4.0129	2.3E+04	1	2	2
5096	29.32	707.2506	709.2513	473.1923	2.0007	1.2E+04	1	1	3
5100	29.34	597.3020	599.3014	363.2437	1.9994	4.7E+04	1	1	3
5102	29.35	749.2285	751.2283	515.1702	1.9998	8.5E+03	1	1	2
5105	29.36	705.2520	707.2501	471.1937	1.9981	7.5E+04	1	1	1
5109	29.37	443.1352	445.1417	418.1538	2.0065	6.8E+03	2	2	5
5111	29.37	643.1338	645.1349	409.0755	2.0011	1.5E+04	1	1	4
5115	29.39	637.1225	641.1346	170.0131	4.0122	2.0E+04	1	2	2
5118	29.39	639.1221	643.1338	172.0127	4.0117	7.4E+03	1	2	1
5121	29.41	751.2274	753.2276	517.1691	2.0002	1.1E+04	1	1	6
5128	29.45	700.3464	702.3594	466.2881	2.0130	1.4E+04	1	1	2
5129	29.46	757.3232	759.3308	523.2649	2.0076	1.2E+04	1	1	4
5130	29.46	707.2508	709.2503	473.1925	1.9995	4.1E+04	1	1	3
5132	29.49	468.2211	470.2247	234.1627	2.0036	6.1E+03	1	1	3
5142	29.59	536.2241	538.2298	302.1658	2.0057	3.0E+04	1	1	3
5151	29.66	595.2867	597.2932	361.2284	2.0065	1.1E+05	1	1	6
5159	29.71	737.3477	739.3497	503.2894	2.0020	1.5E+04	1	1	6
5162	29.72	705.2518	707.2519	471.1935	2.0001	7.7E+03	1	1	1
5175	29.78	738.3600	742.3680	271.2507	4.0080	1.7E+04	1	2	14
5179	29.80	899.2880	903.2958	432.1786	4.0078	7.8E+03	1	2	1
5190	29.82	597.3001	599.3014	363.2417	2.0013	2.1E+04	1	1	13
5192	29.83	577.1937	581.2043	110.0844	4.0106	8.4E+03	1	2	3
5196	29.84	757.3227	759.3294	523.2643	2.0067	9.3E+03	1	1	3
5207	29.87	921.2664	925.2793	454.1570	4.0130	1.0E+04	1	2	2
5208	29.87	556.2757	558.2767	322.2174	2.0010	6.3E+04	1	1	1
5210	29.87	542.2597	544.2650	308.2014	2.0053	7.0E+03	1	1	1
5212	29.87	789.2996	791.3062	555.2413	2.0066	1.6E+04	1	1	1
5213	29.87	606.3458	608.3514	372.2875	2.0055	8.9E+03	1	1	15
5225	29.90	614.2807	616.2838	380.2224	2.0031	1.4E+04	1	1	1
5230	29.90	711.3332	713.3436	477.2749	2.0104	8.9E+03	1	1	10
5232	29.90	554.2219	556.2274	320.1635	2.0055	3.2E+04	1	1	4
5234	29.91	628.3269	630.3344	394.2686	2.0075	1.1E+04	1	1	1
5236	29.92	449.1573	451.1661	430.1979	2.0088	1.4E+04	2	2	8
5242	29.95	612.2742	614.2798	378.2159	2.0056	5.0E+04	1	1	7
5244	29.97	536.3411	538.3518	302.2827	2.0107	6.3E+03	1	1	5
5247	29.97	499.2250	501.2319	265.1667	2.0070	1.1E+04	1	1	2
5253	30.00	611.3177	613.3194	377.2593	2.0018	2.5E+04	1	1	2
5261	30.04	757.3217	759.3283	523.2634	2.0066	1.0E+04	1	1	3
5265	30.04	536.3545	538.3510	302.2962	1.9965	6.2E+03	1	1	1
5271	30.06	700.3465	702.3505	466.2881	2.0040	1.4E+04	1	1	2
5281	30.11	556.2750	558.2774	322.2167	2.0024	1.2E+04	1	1	1
5284	30.12	499.2257	501.2316	265.1673	2.0060	1.2E+04	1	1	2
5286	30.13	743.4553	745.4578	509.3970	2.0025	2.6E+04	1	1	3
5298	30.20	699.4145	701.4142	465.3562	1.9997	9.1E+03	1	1	1
5303	30.22	499.2248	501.2312	265.1665	2.0064	1.0E+04	1	1	2
5307	30.24	629.2650	631.2645	395.2066	1.9995	5.8E+04	1	1	8

5310	30.26	513.1230	515.1293	279.0646	2.0063	8.1E+03	1	1	1
5314	30.26	627.2588	629.2631	393.2005	2.0043	2.0E+05	1	1	5
5315	30.27	680.2668	682.2750	446.2085	2.0082	7.3E+03	1	1	6
5317	30.28	733.5170	735.5208	499.4587	2.0038	7.9E+03	1	1	2
5320	30.29	757.3235	759.3310	523.2652	2.0075	8.5E+03	1	1	4
5323	30.29	759.3308	761.3338	525.2725	2.0030	1.1E+04	1	1	1
5330	30.33	536.3546	538.3600	302.2963	2.0054	7.1E+03	1	1	1
5332	30.35	611.3176	613.3198	377.2593	2.0022	1.6E+04	1	1	2
5340	30.43	440.2244	442.2307	206.1661	2.0063	3.4E+06	1	1	1
5342	30.46	689.4907	691.4938	455.4324	2.0030	2.8E+04	1	1	1
5344	30.48	536.3563	538.3647	302.2980	2.0084	1.1E+04	1	1	1
5352	30.56	757.3244	759.3286	523.2661	2.0042	1.4E+04	1	1	4
5357	30.65	765.3017	767.3081	531.2433	2.0064	1.1E+04	1	1	1
5358	30.65	539.2600	541.2663	305.2017	2.0063	1.1E+04	1	1	1
5369	30.78	700.3467	702.3504	466.2884	2.0037	1.4E+04	1	1	2
5373	30.80	593.2706	595.2765	359.2123	2.0059	2.7E+04	1	1	2
5377	30.82	533.2819	535.2903	299.2235	2.0084	1.3E+04	1	1	7
5381	30.90	535.3204	537.3244	301.2621	2.0040	9.2E+03	1	1	2
5390	30.94	757.3236	759.3304	523.2653	2.0068	8.8E+03	1	1	4
5398	30.99	479.2685	481.2747	245.2102	2.0061	1.2E+04	1	1	3
5399	30.99	623.2815	625.2885	389.2232	2.0070	1.3E+04	1	1	6
5408	31.19	700.3464	702.3506	466.2881	2.0042	1.5E+04	1	1	2
5414	31.27	757.3226	759.3291	523.2643	2.0065	8.6E+03	1	1	3
5418	31.34	566.2627	568.2668	332.2044	2.0041	1.5E+04	1	1	1
5432	31.43	491.2935	493.3006	257.2351	2.0071	1.3E+04	1	1	4
5436	31.47	454.2403	456.2467	220.1820	2.0064	9.3E+03	1	1	3
5438	31.50	440.2250	442.2301	206.1667	2.0050	2.5E+04	1	1	1
5448	31.61	485.6245	487.6337	503.1324	2.0092	2.1E+04	2	2	13
5450	31.64	757.3246	759.3319	523.2662	2.0073	8.4E+03	1	1	4
5455	31.66	673.4975	675.5059	439.4392	2.0084	8.1E+03	1	1	3
5456	31.66	737.3464	739.3515	503.2880	2.0052	3.4E+04	1	1	4
5460	31.68	454.2407	456.2467	220.1824	2.0060	1.9E+04	1	1	3
5463	31.70	735.3405	737.3473	501.2822	2.0068	3.1E+04	1	1	5
5466	31.73	693.2838	695.2900	459.2254	2.0063	1.1E+04	1	1	1
5469	31.74	612.1782	614.1739	378.1199	1.9957	1.4E+04	1	1	11
5473	31.76	700.3454	702.3482	466.2870	2.0028	1.2E+04	1	1	2
5475	31.77	345.1389	347.1456	222.1611	2.0067	3.5E+04	2	2	2
5476	31.77	635.1478	637.1462	401.0894	1.9985	2.6E+04	1	1	4
5477	31.77	759.3326	761.3429	525.2742	2.0104	6.8E+03	1	1	5
5478	31.77	747.3234	749.3247	513.2651	2.0013	1.2E+04	1	1	1
5479	31.79	757.3220	759.3285	523.2636	2.0066	7.5E+03	1	1	3
5480	31.79	674.4816	676.4821	440.4232	2.0006	7.7E+03	1	1	1
5483	31.82	737.3488	739.3558	503.2904	2.0070	2.8E+04	1	1	6
5486	31.84	454.2410	456.2462	220.1827	2.0052	3.5E+04	1	1	3
5487	31.84	657.2588	659.2629	423.2005	2.0041	1.7E+04	1	1	5
5488	31.85	700.3463	702.3532	466.2880	2.0068	1.2E+04	1	1	2
5494	31.89	655.2533	657.2582	421.1949	2.0050	2.0E+04	1	1	6
5499	31.98	582.2371	584.2412	348.1787	2.0041	7.5E+03	1	1	6

5500	31.98	669.3328	671.3398	435.2744	2.0071	1.5E+04	1	1	11

Supplemental Table T4. List of significant metabolites.

ID	rt (min)	mz_light	mz	nCharge	nTag	VIP Score (large vs. negative)	Fold Change Medium vs. Negative	Fold Change Large vs. Negative	Identification	Source
28	2.05	581.1207	347.0623	1	1	1.951	0.64	0.51	Adenosine monophosphate	Dns-lib
129	2.92	424.1174	190.0591	1	1	2.384	0.94	0.62	Glycyl-Aspartate	HMDB
156	3.14	518.1337	284.0753	1	1	2.260	1.66	2.74		
164	3.22	422.2100	188.1517	1	1	2.391	1.57	2.58		
180	3.36	535.1729	301.1146	1	1	2.358	1.05	0.69		
919	6.51	627.3779	393.3195	1	1	1.951	0.81	0.55		
1120	6.77	516.3147	49.2054	1	2	2.348	0.91	0.79	Xanthosine	HMDB
1357	6.98	365.1165	131.0582	1	1	2.386	1.08	1.69	Unknown	
1438	7.03	611.0893	754.0620	2	2	2.175	0.69	0.41		
1472	7.06	591.3602	357.3019	1	1	2.080	1.00	0.51		
1631	7.82	452.1842	218.1259	1	1	1.565	0.86	0.54	Threoninyl-Valine	HMDB
1694	8.29	601.1473	367.0889	1	1	2.125	0.52	0.51		
1733	8.34	659.1510	425.0927	1	1	2.188	0.47	0.45		
1734	8.34	556.1243	322.0660	1	1	2.234	0.49	0.46		
1735	8.34	584.1187	350.0604	1	1	2.199	0.66	0.50		
1868	8.56	633.2566	798.3965	2	2	2.478	0.96	0.77		
1939	8.94	407.5895	347.0624	2	2	1.939	0.59	0.50		
2001	9.76	414.5979	361.0792	2	2	2.124	2.16	2.33		
2025	9.97	315.1086	162.1006	2	2	2.499	0.86	0.63	Unknown	
2027	9.98	792.2656	325.1563	1	2	2.395	1.00	0.66		
2029	9.99	396.1351	324.1536	2	2	2.516	1.01	0.64		
2032	10.02	397.1348	326.1529	2	2	2.463	1.03	0.68		
2096	10.56	697.3207	230.2114	1	2	2.330	1.11	3.04		
2106	10.68	419.1107	370.1047	2	2	2.328	1.14	2.26		
2108	10.68	420.1117	372.1067	2	2	2.432	1.16	2.38		
2130	10.86	382.6482	297.1798	2	2	1.718	1.27	2.81		
2145	11.00	418.5910	369.0653	2	2	1.742	1.97	4.07		
2149	11.02	419.0931	185.0348	1	1	1.759	1.88	3.98		
2153	11.03	419.5908	371.0650	2	2	1.791	1.94	3.62		
2174	11.20	379.2159	145.1576	1	1	2.642	2.04	3.08	Spermidine	Dns-lib
2324	11.70	346.0868	112.0285	1	1	1.897	1.88	2.91	Uracil	Dns-lib
2812	13.59	625.2693	158.1600	1	2	2.228	3.14	3.81		
3103	15.96	370.0567	271.9967	2	2	2.638	3.14	9.71		
3452	19.19	904.3773	437.2680	1	2	1.936	0.73	2.56		
3488	19.45	359.1018	250.0870	2	2	2.364	1.65	2.02		
3497	19.54	358.6008	249.0850	2	2	2.052	1.32	2.06		
3706	22.46	357.4276	123.3693	1	1	2.442	1.02	2.46		
3850	23.13	297.0848	126.0529	2	2	2.351	1.46	1.65		
3852	23.13	301.0761	134.0355	2	2	2.037	2.81	2.58		
4157	24.89	303.0776	138.0386	2	2	2.439	1.46	1.77		
4384	26.02	370.0961	136.0378	1	1	2.414	1.36	1.76		
4476	26.55	290.0715	112.0264	2	2	1.786	1.44	3.08		
4487	26.58	294.5652	121.0138	2	2	2.334	1.24	1.65		
4533	26.82	310.1231	76.0648	1	1	1.635	0.60	0.43		
4550	26.88	738.2906	504.2322	1	1	1.895	0.55	0.53		
4586	26.99	813.3229	579.2646	1	1	1.986	0.52	0.54		
4672	27.30	755.3170	521.2586	1	1	2.022	0.51	0.53		
4715	27.51	291.0831	57.0248	1	1	1.740	0.82	0.27		
4748	27.60	375.4752	141.4169	1	1	2.061	N.A.	0.43		
4982	28.94	545.4018	78.2925	1	2	1.773	0.44	0.50		
5039	29.12	280.8565	46.7982	1	1	2.091	0.39	0.31		
5212	29.87	789.2996	555.2413	1	1	1.946	0.60	0.55		

Supplemental Table T5. Classification results.

ID	Validation dataset	Case#	Date produced	Prediction results	Real classification result
37	VS_2	CA5927-4	Feb-14	no	no
38	VS_2	CA5927-7	Feb-14	yes	yes
39	VS_2	CA5938-6	Feb-14	no	no
40	VS_2	CA5938-7	Feb-14	yes	yes
41	VS_2	CA5940-3	Feb-14	yes	no
42	VS_2	CA5940-12	Feb-14	yes	yes
43	VS_2	CA5962-6	Apr-14	yes	yes
44	VS_2	CA5962-8	Apr-14	no	no
45	VS_2	CA5964-8	Apr-14	yes	yes
46	VS_2	CA5964-9	Apr-14	no	no
47	VS_2	CA5965-2	Apr-14	no	no
48	VS_2	CA5965-8	Apr-14	yes	yes
49	VS_2	CA6032-10	Jul-14	yes	yes
50	VS_2	CA6035-8	Jul-14	yes	yes
51	VS_2	CA6035-11	Jul-14	no	no
52	VS_2	CA6032-11	Jul-14	no	no
53	VS_2	CA6037-7	Jul-14	no	no
54	VS_2	CA6037-10	Jul-14	no	yes
55	VS_2	CA6038-9	Jul-14	yes	yes
56	VS_2	CA6038-10	Jul-14	yes	no
57	VS_2	CA6046-4	Jul-14	yes	yes
58	VS_2	CA6046-12	Jul-14	no	no
59	VS_2	CA6049-8	Jul-14	yes	yes
60	VS_2	CA6049-10	Jul-14	no	no

(yellow)	Not correctly predicted

Supplemental Table T6. List of metabolites used for group classification.

	ID	Name	rt	mz_light	Monoisotopic molecular mass	intensity	fold change (tumor vs. negative)	p value	AUC
1	28	Adenosine monophosphate	2.05	581.1207	347.0623	1.9E+04	0.53	4.6E-03	0.821
2	1357	Unknown ID1357*	6.98	365.1165	131.0582	1.3E+04	1.55	1.8E-03	0.881
3	2025	Unknown ID2025**	9.97	315.1086	162.1006	9.9E+03	0.68	3.5E-04	0.878
4	2174	Spermidine	11.20	379.2159	145.1576	1.6E+04	2.91	8.5E-03	0.849
5	2324	Uracil	11.70	346.0868	112.0285	8.1E+05	2.67	8.0E-03	0.913
6	2106	Ophthalmic acid +HPO3***	10.68	419.1107	370.1047	1.5E+04	2.00	3.1E-03	0.821
7	2149	2,3-Diaminopropionic acid +HPO3****	11.02	419.0931	185.0348	4.1E+04	3.50	1.7E-02	0.827

*confirmed to be not 4-hydroxyproline.
**confirmed to be not 5-hydroxylysine.
***based on mass match alone, it was proposed to be a phospholyated form of ophthalmic acid.
****based on mass match alone, it was proposed to be a phospholyated form of 2,3-diaminopropionic acid.

Supplemental Table T7. List of ratio values of 7 potential biomarkers. The ratios were preprocessed with auto scaling (i.e., ratio subtracted by the mean and then devided by standard deviation).

	ID	RT	mz_light	mz_heavy	Monoisotopic molecular mass	Distance	Intensity	nTag	nCharge
1	28	2.05	581.1207	583.1276	347.0623	2.0070	1.9E+04	1	1
2	1357	6.98	365.1165	367.1222	131.0582	2.0057	1.3E+04	1	1
3	2025	9.97	315.1086	317.1155	162.1006	2.0069	9.9E+03	2	2
4	2174	11.20	379.2159	381.2219	145.1576	2.0060	1.6E+04	1	1
5	2324	11.70	346.0868	348.0931	112.0285	2.0063	8.1E+05	1	1
6	2106	10.68	419.1107	421.1172	370.1047	2.0065	1.5E+04	2	2
7	2149	11.02	419.0931	421.0988	185.0348	2.0057	4.1E+04	1	1

	Training_1	Training_10	Training_11	Training_15	Training_16	Training_18	Training_20	Training_21	Training_22	Training_24	Training_3	Training_6	Training_9	Training_4	Training_5	Training_12	Training_13	Training_14	Training_17	Training_7	Training_8	Training_19	Training_2	Training_23	Training_25
	Tumor	Tumor	Tumor	Tumor	Tumor	Tumor	Tumor	Tumor	Tumor	Tumor	Tumor	Tumor	Tumor	Negative	Negative	Negative	Negative	Negative	Negative	Negative	Negative	Negative	Negative	Negative	Negative
1	-0.11	-0.18	-0.87	0.21	-1.39	-0.41	-1.05	-0.93	-0.64	-0.85	0.40	-0.27	-0.97	1.23	-0.60	0.50	1.14	1.68	2.53	1.33	-1.05	0.19	0.58	0.36	-0.85
2	1.70	0.62	0.90	-0.25	-0.32	0.93	3.08	1.28	0.58	0.20	-0.63	-0.70	-0.01	-0.46	-0.87	-1.19	-0.94	-0.87	-0.70	0.13	-0.18	-0.74	-1.02	0.03	-0.56
3	-1.44	-1.13	-1.24	0.41	-1.24	-0.68	-1.20	-0.19	0.04	-1.68	0.45	1.01	-1.13	0.98	0.26	1.50	0.75	1.24	0.41	1.39	1.05	0.37	0.45	0.56	-0.94
4	0.00	1.66	-0.05	2.12	0.00	0.00	0.00	0.00	0.37	0.44	0.00	0.00	0.12	-0.62	-1.22	0.00	0.00	-1.06	0.00	-0.43	0.00	-0.02	-1.06	-0.28	0.00
5	0.29	-0.23	1.08	-0.02	0.34	0.61	3.94	-0.08	-0.41	0.93	-0.48	-0.30	0.80	-0.83	-0.42	-0.39	-0.69	-0.76	-0.60	-0.42	-0.35	-0.89	-0.79	-0.66	0.34
6	0.69	-0.37	1.33	-0.51	2.84	1.40	1.35	1.36	-0.21	0.51	-0.76	-0.92	0.37	-0.41	-0.39	-0.69	-1.11	-0.79	-1.38	-0.03	-0.27	-0.37	-0.76	-0.26	-0.61
7	-0.43	1.21	-0.65	-0.19	3.60	0.06	2.04	0.61	-0.02	0.04	0.00	-0.52	0.05	0.16	0.20	-0.39	-0.72	-0.49	-0.67	-0.78	-0.75	-0.71	-0.61	-0.69	-0.34

	Testing_1	Testing_2	Testing_3	Testing_4	Testing_5	Testing_6	Testing_7	Testing_8	Testing_9	Testing_10	Testing_11	Testing_12	Testing_13	Testing_14	Testing_15	Testing_16	Testing_17	Testing_18	Testing_19	Testing_20	Testing_21	Testing_22	Testing_23	Testing_24	Testing_25	Testing_26	Testing_27	Testing_28	Testing_29	Testing_30	Testing_31	Testing_32	Testing_33	Testing_34	Testing_35	Testing_36
	Tumor	Negative	Negative	Tumor	Negative	Tumor	Tumor	Negative	Tumor	Negative	Tumor	Negative	Tumor	Negative	Tumor	Negative	Negative	Tumor	Tumor	Negative	Tumor	Negative	Tumor	Negative	Negative	Tumor	Tumor	Tumor	Tumor	Negative	Tumor	Negative	Tumor	Negative	Tumor	Negative
1	0.22	1.31	-2.05	-1.53	-1.74	-1.21	-1.07	-0.73	-1.38	-1.30	-1.05	-0.30	1.03	-0.84	1.39	0.82	-0.34	-0.22	-0.38	0.79	0.68	0.07	1.16	0.93	0.67	-0.79	-0.29	-0.63	1.72	2.06	1.56	0.48	-0.20	1.27	0.99	1.93
2	2.78	-1.50	-0.87	-0.10	-1.56	0.70	-0.03	-0.65	-0.03	-0.44	1.40	-0.54	2.38	-1.27	0.00	0.00	0.00	0.00	-0.96	-0.28	1.62	-0.36	-0.07	0.99	0.00	0.63	-0.76	0.00	0.00	0.01	0.63	-0.39	-0.80	-1.49	0.00	0.22
3	-0.10	1.14	-1.35	-0.99	-1.64	-1.29	0.55	0.61	-1.64	-0.76	-1.64	0.13	0.02	-2.05	-0.76	0.43	-0.70	-0.94	-0.81	-0.09	-0.81	-0.52	0.55	-0.64	1.02	0.31	0.19	2.92	0.31	1.44	-0.87	0.43	-0.28	0.90	0.02	1.20
4	-0.29	0.06	1.01	0.74	-1.04	-0.26	-1.05	-0.62	0.00	-0.64	-0.75	-0.10	-0.70	0.00	0.90	0.85	0.12	-0.91	2.35	2.05	-1.13	-0.45	0.20	-0.37	0.74	0.31	3.35	0.17	1.39	0.80	-0.56	-0.10	0.69	-1.00	-0.48	0.00
5	0.82	-0.89	0.74	1.80	-0.78	1.63	0.65	-0.07	-0.32	-0.32	0.19	-0.87	-0.76	-1.22	-0.13	-0.70	-0.51	-0.47	-0.81	-0.74	-0.59	-1.11	-1.02	-0.89	-0.62	0.72	2.18	3.51	-1.00	-0.76	-0.45	-0.74	0.15	-0.78	-0.36	-0.95
6	0.63	-0.50	-0.17	3.25	0.00	0.23	0.00	-0.75	-0.31	-0.71	-0.11	-0.66	0.35	-1.16	0.40	-0.63	-0.88	-0.57	-0.32	-0.03	-0.82	-0.79	-0.65	-0.64	-0.67	-0.07	3.41	1.71	-0.14	-0.04	-0.25	-0.71	0.23	-0.74	0.23	-0.92
7	0.65	-0.48	-0.18	3.20	0.00	0.22	0.00	-0.74	-0.29	-0.70	-0.10	-0.65	0.38	-1.12	0.42	-0.62	-0.86	-0.55	-0.30	0.01	-0.82	-0.77	-0.61	-0.63	-0.69	-0.10	3.40	1.69	-0.16	-0.07	-0.26	-0.70	0.20	-0.75	0.24	-0.91

	Validation_1	Validation_2	Validation_3	Validation_4	Validation_5	Validation_6	Validation_7	Validation_8	Validation_9	Validation_10	Validation_11	Validation_12	Validation_13	Validation_14	Validation_15	Validation_16	Validation_17	Validation_18	Validation_19	Validation_20	Validation_21	Validation_22	Validation_23	Validation_24
	Negative	Tumor	Negative	Tumor	Negative	Tumor	Tumor	Negative	Tumor	Negative	Negative	Tumor	Tumor	Tumor	Negative	Negative	Negative	Tumor	Tumor	Negative	Tumor	Negative	Tumor	Negative
1	1.41	0.89	0.63	0.19	0.51	-0.66	-1.01	-0.03	-0.34	0.32	-0.05	-1.19	-1.28	-0.32	0.46	-1.30	0.05	0.63	0.46	-1.36	-0.73	0.43	-0.02	-0.73
2	-0.14	0.12	-0.25	2.27	-0.58	-0.77	-0.36	0.30	1.76	0.77	-0.94	1.65	0.23	-0.43	-1.10	-0.67	-0.47	-0.28	0.77	-0.36	0.23	0.45	-0.67	-0.77
3	1.62	0.13	0.90	-0.52	0.19	0.55	-0.40	1.44	-1.23	1.26	2.09	0.25	0.25	-0.28	0.31	0.25	1.08	0.73	-0.90	-1.17	-1.11	0.49	-0.64	0.43
4	-0.02	-1.17	-0.67	-0.13	0.23	-0.73	2.02	0.52	-0.37	-1.22	1.37	-0.73	-1.24	0.23	-0.70	-1.08	-0.91	-0.13	1.04	1.10	-0.77	-0.77	-1.27	0.14
5	-0.53	1.06	-0.91	0.95	0.80	0.95	1.82	-0.23	1.80	0.17	0.89	0.00	1.52	-0.21	-0.53	0.15	-0.64	0.15	-0.81	-1.09	1.08	-0.21	-0.32	-0.36
6	-0.41	0.11	-0.40	0.35	-0.01	0.51	3.23	-0.79	0.61	-0.31	0.11	0.79	1.45	-0.45	-0.35	0.09	-1.00	0.69	-0.60	-0.89	1.61	-0.19	-0.87	-0.59
7	-0.39	0.09	-0.38	0.37	0.01	0.49	3.22	-0.81	0.59	-0.29	0.09	0.82	1.43	-0.43	-0.32	0.11	-0.96	0.69	-0.58	-0.91	1.59	-0.21	-0.90	-0.62
